# Metabolomic prediction of severe maternal and newborn complications in preeclampsia

**DOI:** 10.1007/s11306-024-02123-0

**Published:** 2024-05-18

**Authors:** Jay Idler, Onur Turkoglu, Ali Yilmaz, Nadia Ashrafi, Marta Szymanska, Ilyas Ustun, Kara Patek, Amy Whitten, Stewart F. Graham, Ray O. Bahado-Singh

**Affiliations:** 1 Drexel College of Medicine, Philadelphia, PA USA; 2 Department of Obstetrics and Gynecology, Allegheny Health Network, 4815 Liberty Ave., Pittsburgh, PA 15224 USA; 3 Department of Obstetrics and Gynecology, Beaumont Health System, Royal Oak, MI USA; 4 Oakland University School of Medicine, Rochester, MI USA; 5 Department of Obstetrics and Gynecology, Wayne State University-Detroit Medical Center, Detroit, MI USA; 6 Depaul University, Chicago, IL USA

**Keywords:** Metabolomics, Preeclampsia, Adverse outcomes, Nuclear magnetic resonance, Mass spectrometry

## Abstract

**Introduction:**

Preeclampsia (PreE) remains a major source of maternal and newborn complications. Prenatal prediction of these complications could significantly improve pregnancy management.

**Objectives:**

Using metabolomic analysis we investigated the prenatal prediction of maternal and newborn complications in early and late PreE and investigated the pathogenesis of such complications.

**Methods:**

Serum samples from 76 cases of PreE (36 early-onset and 40 late-onset), and 40 unaffected controls were collected. Direct Injection Liquid Chromatography–Mass Spectrometry combined with Nuclear Magnetic Resonance (NMR) spectroscopy was performed. Logistic regression analysis was used to generate models for prediction of adverse maternal and neonatal outcomes in patients with PreE. Metabolite set enrichment analysis (MSEA) was used to identify the most dysregulated metabolites and pathways in PreE.

**Results:**

Forty-three metabolites were significantly altered (*p* < 0.05) in PreE cases with maternal complications and 162 metabolites were altered in PreE cases with newborn adverse outcomes. The top metabolite prediction model achieved an area under the receiver operating characteristic curve (AUC) = 0.806 (0.660–0.952) for predicting adverse maternal outcomes in early-onset PreE, while the AUC for late-onset PreE was 0.843 (0.712–0.974). For the prediction of adverse newborn outcomes, regression models achieved an AUC = 0.828 (0.674–0.982) in early-onset PreE and 0.911 (0.828–0.994) in late-onset PreE. Profound alterations of lipid metabolism were associated with adverse outcomes.

**Conclusion:**

Prenatal metabolomic markers achieved robust prediction, superior to conventional markers for the prediction of adverse maternal and newborn outcomes in patients with PreE. We report for the first-time the prediction and metabolomic basis of adverse maternal and newborn outcomes in patients with PreE.

**Supplementary Information:**

The online version contains supplementary material available at 10.1007/s11306-024-02123-0.

## Introduction

Preeclampsia (PreE) remains a major obstetric challenge, occurring in 2–8% of pregnancies and globally accounting for approximately 63,000 deaths annually (Duley, 2009; Vigil-De Gracia, 2009). Despite advances in clinical diagnosis and management of PreE, decades of research have not definitively established its pathogenesis. More recently, the concept of PreE as two different disorders, admittedly with extensive overlap, has emerged (Steegers et al., 2010; Valensise et al., 2008). In this conceptualization, early-onset PreE, diagnosed prior to 34 weeks, has as its dominant pathogenic feature, failure of trophoblastic invasion (Brosens et al., 2011; Ogge et al., 2011). For late-onset PreE, diagnosed at ≥34 weeks, the pathogenesis is reportedly more heterogenous, linked to maternal metabolic disorders, inflammation, and underlying vascular disease (Bahado-Singh et al., 2012; Raymond & Peterson, 2011).

Metabolomics utilizes sophisticated analytic chemistry techniques to characterize the substrates and byproducts of cellular metabolism. It has advanced our understanding of both normal physiology and disease pathophysiology (Wishart, 2019) and is uniquely suited to help elucidate the mechanisms of complex disorders with multifactorial etiologies (Pite et al., 2020). Not surprisingly, there is now a growing literature on PreE and metabolomics (Benny et al., 2020; Kelly et al., 2017). The focus by us and others has been the identification of predictive biomarkers (Bahado-Singh et al., 2013; Nobakht, 2018), disease mechanisms (Bahado-Singh et al., 2017; Kuc et al., 2014), and distinguishing early from late-onset PreE (Kawasaki et al., 2019; Savasan et al., 2014). Metabolomic alterations have been noted prior to disease presentation which may persist throughout pregnancy (Pinto et al., 2014). Several models have been utilized for disease prediction with notably less success in late-compared to early-onset PreE (Bahado-Singh et al., 2015; Koster et al., 2015; Kuc et al., 2014). Despite clinical differences in their presentations, common metabolites in early- and late-onset PreE suggest overlapping biological mechanisms (Braekke et al., 2007; Leavey et al., 2016).

Both early- and late-onset PreE are associated with increased maternal, fetal, and newborn morbidity and mortality (Lisonkova & Joseph, 2013; Lisonkova et al., 2014). It is surprising that more attention has not been paid to the metabolomic prediction of neonatal and maternal adverse outcomes in affected pregnancies. Such information has the potential to be clinically impactful. Currently, classification of PreE severity relies heavily on a combination of subjective complaints and variable clinical findings. Great significance is placed on the gestational age at clinical presentation, more precisely the gestational age at initial diagnosis, which may have a tenuous relationship to underlying biology. An objective laboratory-based categorization of PreE severity would represent a significant advance over the current situation and better elucidate the disease pathogenesis with an eye to the future development of more targeted therapeutics. The objective of this study was to ascertain the feasibility of predicting severe maternal and newborn complications in both early- and late-onset PreE. While late-onset PreE is generally a milder disorder, it may be associated with significant morbidities (Lisonkova & Joseph, 2013; Lisonkova et al., 2014). As late-onset PreE is significantly more common than its early-onset counterpart, its cumulative clinical significance is further magnified. We aimed to investigate the metabolomic abnormalities that underly or are associated with pregnancy complications. We posited that the metabolomic perturbations are likely to be more pronounced at the time of diagnosis of the clinical disorder than in the first-trimester, remote from its clinical presentation and was thus a good point from which to start.

## Methods

### Study population and sample collection

This was a prospective study of metabolomic changes in the maternal blood in PreE. The William Beaumont Hospital, Royal Oak, Michigan Institutional review board approved this project (#2015-136). Study subjects provided an informed written consent. The recruitment of PreE cases were performed at or soon after the clinical diagnosis was made. PreE was defined based on standard ACOG criteria (2020). Diagnostic features of PreE were systolic blood pressure of 140 mmHg or more or diastolic blood pressure of 90 mmHg or more on two occasions at least 4 h apart after 20 weeks’ gestation in women who were previously normotensive. In addition to hypertension, the presence of proteinuria (300 mg or more per 24-h collection, or protein/creatinine ratio of 0.3 or more) or other pertinent laboratory abnormalities or new symptoms were utilized to support the diagnosis of PreE. Beyond proteinuria, other laboratory values included new onset of thrombocytopenia (platelet less than 100 × 10^9^/L), renal insufficiency (serum creatinine greater than 1.1 mg/dL or doubling of serum creatinine without underlying renal disease), impaired liver function (transaminases at least twice normal). Symptoms included pulmonary edema, new-onset headache (unresponsive to medication and not accounted for by any other diagnosis), or visual symptoms. Only cases which met the above criteria for PreE diagnosis were included in the study. We excluded cases with known or suspected fetal anomalies or multifetal gestation. Early-onset PreE was defined as a diagnosis prior to 34 weeks and late-onset was includes cases developed at 34 weeks’ gestation or later. Controls were recruited from among women in the mid-or third trimester who did not have a hypertensive disorder during the course of pregnancy or postpartum period. Maternal demographics, clinical data and neonatal outcomes were recorded following a prospective follow up of each pregnancy and infant after delivery.

### Sample collection and metabolomics analysis

Blood samples were collected from each participant following at least 4 h of fasting and allowed to sit for approximately 15 min. Samples were centrifuged at 3000 rpm for 10 min and the serum was aliquoted in 0.5 mL quantities and placed into cryovial screw cap tubes. The samples were then immediately stored in a −80 °C freezer and were not thawed until metabolomic analysis. Following the completion of prospective cohort, combined Direct Injection Liquid chromatography–Mass spectrometry (LC–MS) and Nuclear Magnetic resonance (NMR) spectroscopy were used to perform metabolomic analysis on serum samples as detailed in the supplemental methods section.

### Statistical analysis

MetaboAnalyst (v 5.0) was used to compare metabolite concentrations between PreE cases and controls (Pang et al., 2021). Student’s *t*-test was used to compare means between groups for normally distributed data. Mann–Whitney *U* test was utilized for non-normally distributed data. Kolmogorov–Smirnov test was used to evaluate normality of distribution of values. Between group comparison was performed using chi-squared for categorical variables. Significance was defined as a *p*-value < 0.05. To simplify metabolite values, Gaussian distribution was achieved by data normalization for conventional statistics as previously described (Bahado-Singh et al., 2022). Data was normalized to the median and auto scaling was performed. Partial least squares discriminant analysis (PLS-DA) plots were performed to visually represent the discrimination of the study groups based on metabolomic profiles of each group (i.e. early- and late-onset PreE vs. controls) (Bahado-Singh et al., 2022). PLS-DA plots were cross-validated using permutation testing (2000 iterations) to determine if any observed separation in the representative scores’ plots achieved statistical significance.

Clinical variables and metabolites were used to develop logistic regression models for the prediction of severe adverse neonatal and maternal outcomes. Predictive models were built using all PreE cases, and within each subgroup of late- and early-onset PreE cases, respectively. Outcome measures of adverse maternal and newborn outcomes seen with PreE were defined based on standardized criteria (2020; Venkatesh et al., 2020; von Dadelszen et al., 2011; Zhang et al., 2001). Adverse maternal outcomes included placental abruption, venous thromboembolic events, pulmonary edema, liver problems (transaminitis), renal failure, HELLP, postpartum hemorrhage, and eclampsia. Adverse neonatal outcomes included neonatal intensive care unit admissions >29 days, intraventricular hemorrhage, periventricular leukomalacia, necrotizing enterocolitis, retinopathy of prematurity, respiratory distress syndrome, and bronchopulmonary dysplasia (2020; Venkatesh et al., 2020; von Dadelszen et al., 2011). Regression models were generated based on clinical factors conventionally used to identify women at elevated risk for PreE and combined clinical and metabolomic models. A stepwise variable selection method was used to optimize metabolite models. A tenfold cross-validation (CV) technique, achieved by repeated randomly division of the entire sample data into ten equal-sized subsets, was used to ensure the generalizability of our logistic regression models. Model performance was determined based on the calculation of the area under the receiver operating characteristics curve (AUC) and the associated, 95% confidence interval (CI), and sensitivity and specificity values.

### Metabolite set enrichment analysis

Metabolite set enrichment analysis (MSEA), determines if a group of functionally related metabolites in different biochemical pathways are altered. MSEA was used to identify biologically meaningful patterns in metabolite concentrations as previously described (Bahado-Singh et al., 2022). *Homo sapiens* (human) pathway library was chosen and all the compounds in the selected pathways were used when referencing the specific metabolome. MSEA eliminates the preselection of compounds in which significance is based on arbitrary cutoff thresholds. This allows us to identify smaller, but persistent biochemical changes that would escape recognition with the use of more standard approaches. The fold enrichment and *p*-values were used to visually represent the results of the MSEA.

## Results

Seventy-six cases of PreE and 40 unaffected controls were included in the study. Among the PreE group, 36 patients were diagnosed with early-onset and 40 patients had late-onset PreE. Table 1 compared maternal demographics and clinical variables between PreE cases (early + late) and controls. When all PreE cases were compared to controls, there was an increased frequency of risk factors and co-morbidities including nulliparity, higher body mass index (BMI), chronic hypertension, pregestational diabetes, and history of cesarean section. There was a statistically significantly (*p* ≤ 0.02) increased frequency of perinatal adverse outcomes including fetal growth restriction (estimated fetal weight or abdominal circumference less than the 10th percentile), cesarean birth, small for gestational age newborns and a delivery at an earlier gestational age.

**Table 1:** Comparison of demographics and clinical factors between PreE cases (early + late) vs. controls

Parameter	PreE (early + late)	Controls	*p*-Value
Number of patients	76	40	N/A
Age, years, mean (SD)	30.48 (5.21)	30.50 (6.21)	0.134^t^
Race	Caucasian (*N* = 56) Black (*N* = 14) Asian (*N* = 4) Hispanic (*N* = 0) Other (*N* = 2)	Caucasian (*N* = 33) Black (*N* = 5) Asian (*N* = 1) Hispanic (*N* = 0) Other (*N* = 1)	0.182^c^
Nulliparous, *n* (%)	48 (63.1%)	12 (30.0%)	**<0.01**^**c**^
BMI, mean (SD)	35.07 (7.87)	31.24 (5.88)	**0.017**^**u**^
Gestational age at the time of collection (SD)	33.41 (4.03)	36.20 (3.28)	**<0.001**^**u**^
Gestational age at delivery, mean (SD)	34.81 (3.66)	39.11 (1.09)	**<0.001**
GDM	7 (9.2%)	0 (0.0%)	0.113^c^
cHTN	24 (31.6%)	0 (0.0%)	**<0.001**^**c**^
Pregestational diabetes	11 (14.5%)	0 (0.0%)	**0.01**^**c**^
Fetal growth restriction	23 (30.3%)	0 (0.0%)	**<0.001**^**c**^
History of PreE	13 (17.1%)	1 (2.5%)	**0.022**^**c**^
IVF	9 (11.8%)	1 (2.5%)	0.088^c^
Mode of delivery (normal spontaneous vaginal delivery/cesarean section)	SVD 32 (42.1%) C/S 44 (57.9%)	SVD 26 (65.0%) C/S 14 (35.0%)	**0.019**^**c**^
Postpartum hemorrhage	5 (6.6%)	2 (5.0%)	0.734^c^
Newborn weight (g), mean (SD)	2378.20 (982.0)	3364.9 (320.84)	**<0.001**^**u**^
SGA at birth	30 (40.0%)	1 (2.5%)	**<0.001**^**c**^
Apgar’s 5 min, mean (SD)	8.63 (0.65)	8.88 (0.56)	**0.003**^**u**^

All statistically significant parameters were bolded

^t^Independent sample *t*-test

^c^Pearson chi-squared

^u^Mann–Whitney *U* test

The comparison of these risk factors and adverse outcomes between early- and late-onset PreE and controls are presented in supplementary section (Supplemental Tables S1, S2 and S3). There were higher rates of risk factors for PreE and complications and earlier gestational age at delivery in early- vs. late-onset PreE (Table S1). Not surprisingly, there were increased rates of perinatal complications in early-onset PreE compared to the control group (Table S2). Interestingly, there were increased rates of adverse perinatal outcomes in late-onset PreE compared to controls (Table S3). Severe neonatal adverse outcome occurred in 20 (55.6%) early-onset and 7 (17.5%) late-onset PreE cases Table 2. Composite maternal adverse outcome occurred in 13 (36.1%) early-onset and 7 (17.5%) late-onset PreE cases.

**Table 2:** Frequency of adverse outcomes in controls, early- and late-onset PreE patients

Parameter	Early-onset PreE	Late-onset PreE
Number of patients	36	40
Composite maternal adverse outcomes	13 (36.1%)	7 (17.5%)
• Venous thromboembolism	0 (0.0%)	0 (0.0%)
• Placental abruption	2 (5.6%)	0 (0.0%)
• Pulmonary edema	1 (2.8%)	1 (2.5%)
• Transaminitis	7 (19.4%)	4 (10.0%)
• Renal failure	3 (8.3%)	2 (5.0%)
• HELLP	1 (2.8%)	1 (2.5%)
• Postpartum hemorrhage	2 (5.6%)	3 (7.5%)
• Eclampsia	0 (0.0%)	0 (0.0%)
Severe composite neonatal outcomes	20 (55.6%)	7 (17.5%)
• NICU admission >29 days	18 (50.0%)	0 (0.0%)
• Intraventricular hemorrhage	4 (11.1%)	0 (0.0%)
• Periventricular leukomalacia	0 (0.0%)	0 (0.0%)
• Necrotizing enterocolitis	0 (0.0%)	0 (0.0%)
• Retinopathy of prematurity	1 (2.8%)	0 (0.0%)
• Respiratory distress syndrome	17 (47.2%)	7 (17.5%)
• Bronchopulmonary dysplasia	0 (0.0%)	1 (2.5%)

*HELLP* hemolysis, elevated liver function tests, and low platelets

**Fig. 1:**
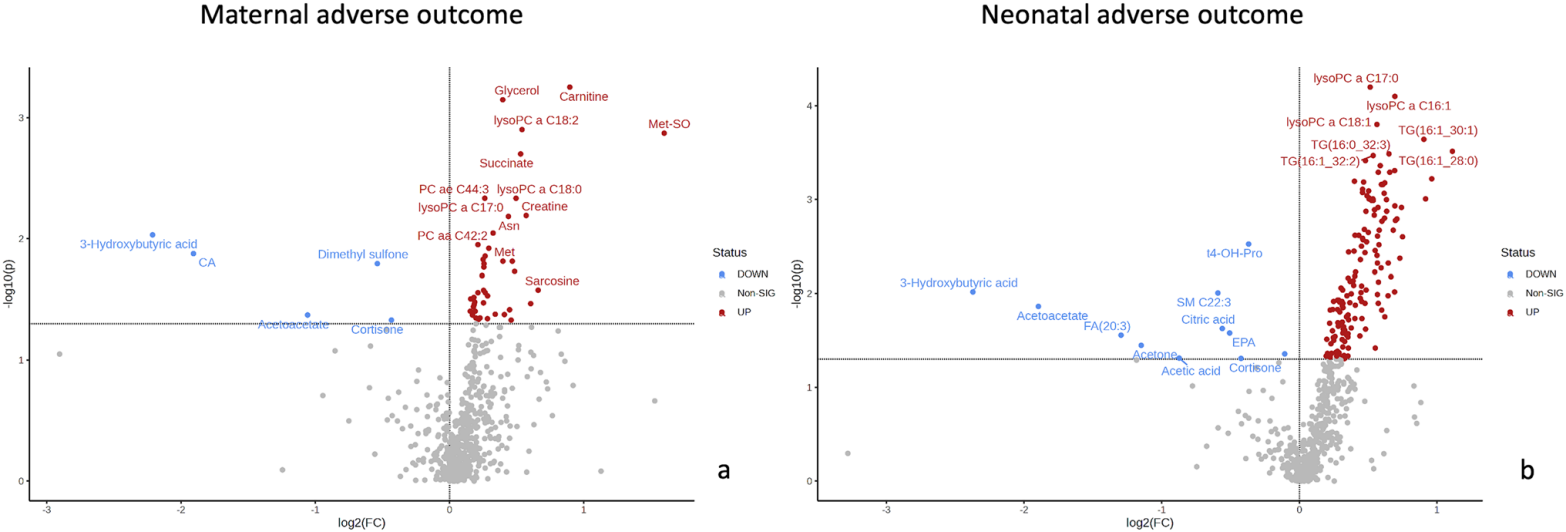
Volcano plots in distinguishing PreE cases with maternal and neonatal adverse outcome

Six hundred and fourteen metabolites were biochemically profiled by combining LC–MS Q500 and ^1^H-NMR allowing deep targeted metabolomic analysis. Supplemental Table S4 demonstrates each compound based on their discovery by LC–MS vs. NMR. Concentration values of the 17 overlapping metabolites were identified between platforms and averaged out. Following exclusion of metabolites with >20% zero values, a total of 521 metabolites remained and were analyzed. Volcano plots (Fig. 1a, b) illustrate the number of variables significantly altered in PreE patients with maternal and neonatal adverse outcomes, respectively. There were 43 prenatal metabolites whose concentrations were significantly altered (*p* < 0.05) in PreE cases with maternal adverse outcome (Supplemental Table S5) compared with those without adverse outcomes. A total of 162 metabolites were significantly altered prenatally (*p* < 0.05) in PreE cases with neonatal adverse outcomes (Supplemental Table S6) compared to PreE without adverse neonatal outcomes. PLS-DA plots demonstrated good visual separation in distinguishing the PreE cases (early and late-onset) with maternal adverse outcomes (Fig. S1a) as well as those with adverse neonatal outcomes (Fig. S1b). While the PLS-DA plots indicated good separation on visual inspection, permutation testing with 2000 repeats did not reach statistical significance (*p* > 0.05). This was likely due to the relatively small number of cases in the study.

In addition to the analysis of adverse outcome prediction, we performed a comparison between the metabolomic profiles of early- vs. late-onset PreE cases vs. controls. There were 235 metabolites with significant concentration differences in early-onset PreE compared to controls (*p*-value < 0.05) (Supplemental Table S7). In contrast, 19 metabolites differed significantly between late-onset PreE compared with controls (*p*-value < 0.05) (Supplemental Table S8) suggesting that late-onset PreE is a ‘milder’ metabolomic disorder. Metabolomic profiles of early- and late-onset PreE groups also significantly differed, with a total of 100 metabolites with *p* < 0.05 (Supplemental Table S9). This finding confirms that clinical classification of early- vs. late-onset PreE does indeed have biological justification.

Logistic regression models for detection of adverse maternal and neonatal outcomes based on metabolites only were developed and compared to clinical based (fetal growth restriction (FGR), Maternal age, Race, Nulliparity, BMI, IVF, Pregestational DM, cHTN) prediction models. Table 3 presents the clinical and metabolite models for the prediction of maternal and neonatal adverse outcomes. Clinical variables alone poorly predicted maternal adverse outcomes in PreE overall AUC = 0.511 (0.361–0.661) and in early-onset AUC = 0.562 (0.357–0.767), where the performance was moderate for late-onset PreE AUC = 0.787 (0.661–0.913). In contrast, the metabolite only models achieved strong predictive performances (AUC > 0.8) for the prediction of adverse maternal outcomes in the overall, early- and late-onset PreE groups. Clinical variables also performed poorly for the prediction of newborn adverse outcomes in all cases AUC = 0.587 (0.447–0.728) early- 0.559 (0.357–0.761) and the late-onset 0.446 (0.285–0.606) PreE groups. In contrast, the metabolite-only prediction models for adverse newborn outcomes were robust. For overall PreE group this was AUC (95% CI) = 0.823 (0.722–0.924), where early-onset PreE = AUC (95% CI) of 0.828 (0.674–0.982) and late-onset PreE group an AUC (95% CI) of 0.828 (0.674–0.982) = 0.911 (0.828–0.994). Incorporation of clinical variables into the metabolite/lipid regression models did not improve the predictive ability, hence, it was not included in the Table 3.

**Table 3:** Clinical and metabolite algorithms in predicting composite severe maternal adverse outcome at birth in PreE cases following tenfold Cross Validation

Group	Clinical and metabolite algorithms	AUC (95% CI)	Sensitivity (%)	Specificity (%)
Composite severe maternal adverse outcome prediction^a^				
All PreE	Clinical variables^b^	0.511 (0.361–0.661)	55.0	57.1%
	TG(22:5_34:1) + lysoPC a C18:0 + lysoPC a C18:2 + Met-SO	0.810 (0.699–0.921)	85.0	69.6
Early-onset PreE	Clinical variables^b^	0.562 (0.357–0.767)	53.8	60.9
	lysoPC a C18:0 + TG(22:5_34:1) + lysoPC a C18:2	0.806 (0.657–0.955)	84.6	69.6
Late-onset PreE	Clinical variables^b^	0.787 (0.661–0.913)	76.5	72.7
	TG(18:1_33:3) + TG(20:3_34:0)	0.843 (0.712–0.974)	94.1	84.8
Severe adverse neonatal outcome prediction^c^				
All PreE	Clinical variables^d^	0.587 (0.447–0.728)	55.6	63.3
	t4-OH-Pro + Cer(d18:0/24:1) + PC aa C34:2	0.823 (0.722–0.924)	81.5	77.6
Early-onset PreE	Clinical variables^d^	0.559 (0.357–0.761)	65.0	75.0
	3-Hydroxybutyric acid, t4-OH-Pro, Hex2Cer(d18:1/26:0)	0.828 (0.674–0.982)	85.0	81.2
Late-onset PreE	Clinical variables^d^	0.446 (0.285–0.606)	58.8	48.5
	TG(16:1_33:1) + TG(20:4_32:1) + TG(16:1_30:1)	0.911 (0.828–0.994)	82.4	84.8

^a^Maternal adverse outcomes: placental abruption, venous thromboembolic events (VTE), pulmonary edema, Transaminitis, Renal failure, HELLP, postpartum hemorrhage (PPH), eclampsia

^b^Clinical variables used for maternal adverse outcome prediction: Maternal age + Race + Nulliparity + BMI + IVF + Pregestational DM + cHTN

^c^Newborn adverse outcomes: NICU admit >29 days, IVH, PVL, NEC, ROP, RDS, BPD

^d^Clinical variables used for severe neonatal adverse outcome prediction: FGR + Maternal age + Race + Nulliparity + BMI + IVF + Pregestational DM + cHTN

**Fig. 2:**
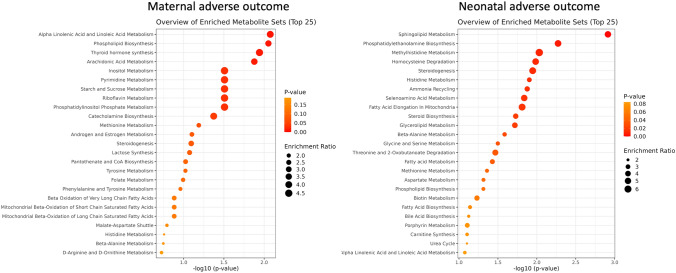
Metabolite set enrichment analysis for the altered pathways in PreE cases with maternal and neonatal adverse outcome

MSEA identified multiple metabolic pathways altered in the serum of women who experienced adverse maternal and neonatal outcomes in early-onset and late-onset PreE. Altered pathways were evaluated with the number of metabolite ‘hits’, enrichment impact, and *p*-values (<0.05) for the significance. “Hits” indicate the number of metabolites in the pathway found to have significant concentration changes in the serum of PreE patients with adverse outcomes. Pathways most significantly dysregulated in patients with adverse outcomes in PreE are those with the highest number of “hits” along with significant *p*-values (Supplemental Table S10). There were ten significantly altered metabolic pathways (*p* < 0.05) in PreE cases who developed adverse maternal outcome (Fig. 2a). The top two significant pathways (*p* < 0.01) included alpha linolenic acid and linoleic acid metabolism and phospholipid biosynthesis. In comparison, there were 18 significantly altered metabolic pathways (*p* < 0.05) in PreE cases who developed severe neonatal adverse outcome (Fig. 2b). The top dysregulated pathways included sphingolipid metabolism, phosphatidylethanolamine biosynthesis, and methylhistidine metabolism (*p* < 0.01).

## Discussion

We report accurate metabolomic markers for the prediction of subsequent severe maternal and newborn complications in PreE. In addition, metabolites appeared superior to conventional clinical/demographic factor for prediction of these outcomes. We found significant dysregulation of multiple biochemical pathways in PreE cases compared with PreE cases that did not develop these complications. To the authors’ knowledge, this is the first metabolomic study of the prediction maternal and neonatal adverse outcome in PreE. Despite the consensus guidelines defining the severe features of PreE and indications for delivery, there is currently no one symptom, sign, or laboratory test accurately predicting adverse maternal and neonatal outcomes (Mirkovic et al., 2020). The ability to predict newborn and maternal outcomes in PreE has potential future clinical benefits. These could include parental counseling, adjusting frequency of monitoring, clinical decision-making with regards to transfer to tertiary level institution, and management decisions such as timing of hospitalization and delivery.

Metabolomic profiles were able to distinguish the PreE cases from controls and those with maternal and neonatal adverse outcomes. Volcano plot provides visual representation of significantly different metabolites based on their *p*-value (<0.05) and fold change, and thus potential candidate biomarkers for the prediction of adverse outcomes (Fig. 1) in PreE. Using the top significant metabolite markers, robust prediction (AUC > 0.80) of maternal adverse outcome was achieved for both early- and late-onset PreE with good diagnostic performance i.e. the prediction of severe newborn outcome was also robust in the early- and late-onset PreE group, with generally higher predictive accuracy in the late-onset group AUC (95%) = 0.828 (0.674–0.982), and 0.911 (0.828–0.994) respectively. Several metabolite markers identified herein have been previously demonstrated in the metabolomics literature for the detection of early- and late-onset preeclampsia (Al-Maiahy et al., 2021; Xue et al., 2021; Yao et al., 2022)., hence, supporting the reproducibility of our results.

When compared with a model based on widely used clinical risk factors only, metabolite models demonstrated superior accuracy across all subgroups. Additionally, the metabolite biomarkers achieved robust predictive accuracies when compared with other conventional markers reported in the literature. A meta-analysis evaluated angiogenic and antiangiogenic factors, including soluble fms-like tyrosine kinase-1 (sFlt-1) and placental growth factor (PlGF) for the prediction of adverse outcomes in PreE (Lim et al., 2021). Despite the promising diagnostic potential of sFlt-1, and PlGF in PreE, the accuracy for adverse outcome prediction remained moderate, with AUCs ranging between 0.68 and 0.79. Additionally, metabolite markers remained accurate in predicting maternal and severe neonatal adverse outcome even in late-onset PreE cases (AUC = 0.911) which is widely recognized to be a milder form of the disease. Conventional markers including sFlt-1, and PlGF are known to have moderate to low accuracy in predicting adverse outcomes in late-onset PreE cases (Lim et al., 2021). Although early-onset PreE has higher rates of maternal and neonatal adverse outcome, late-onset PreE is significantly more prevalent than the early-onset form, hence the prediction of adverse outcomes in this group has more cumulative effect (Wojtowicz et al., 2019). Additionally, late PreE is associated with long term consequences such as increased risk of microvascular disorders (Kenneth et al., 2010) similar to its early-onset counterpart (Stekkinger et al., 2009; Veerbeek et al., 2015).

In addition to the predictive markers, metabolomics also has the advantage of interrogating the metabolic basis of complications developing in mothers and newborns whose pregnancies are affected by PreE. There was an overall pronounced increase in lipid concentrations in cases with adverse outcomes. Similar to significant alterations in metabolites during an uncomplicated pregnancy (Orczyk-Pawilowicz et al., 2016), there are also complex changes in lipids, and lipid abnormalities which are well-established risk factor for the development of PreE (Mitro et al., 2021; Yang et al., 2022). Alterations in maternal blood and placental lipidome have been implicated in the pathogenesis of severe PreE and adverse outcomes (Baig et al., 2013; He et al., 2021). Consistent with other studies, most of the altered lipids in PreE included triglycerides (TG), lysophospholipids and phosphatidylcholines were predictive of both adverse maternal and newborn outcomes (Bahado-Singh et al., 2017; Clausen et al., 2001). A two- to threefold increased risk for the development of PreE has been reported when TG levels are elevated early in pregnancy or persistently elevated throughout gestation (Al-Maiahy et al., 2021; Vrijkotte et al., 2012; Xue et al., 2021).

Linoleic acid metabolism and phospholipid biosynthesis were altered in PreE cases with maternal adverse outcomes in our study. Alpha linolenic acid and linoleic acid are omega 3 fatty acids. Deficiencies of omega-3 fatty acids have been studied with mixed results with respect to their roles in the development of PreE and other obstetric morbidities (Meher et al., 2016; Middleton et al., 2018). An imbalance of fatty acids may predispose patients to PreE and preterm birth, which are leading causes of maternal and neonatal morbidity (Irwinda et al., 2021). Further, oxidative stress in PreE results in increased phospholipid metabolism (He et al., 2021). As noted previously, phospholipid alterations have been described at the level of the placenta as well, with an increased phospholipid concentration in the placenta of patients with PreE (Huang et al., 2013). Aspirin has been demonstrated to reduce the risk of the more severe, early-onset PreE. This in turn would be expected to result in a reduction of associated maternal and consequent neonatal morbidity (Henderson et al., 2021; Rolnik et al., 2017). Nonpregnant patients with hyperlipidemia are commonly treated with statins. As a result, in recent years statins have in turn been studied for the prevention and treatment of PreE, with promising safety profiles and outcomes (Costantine et al., 2016, 2021; Dobert et al., 2021; Smith & Costantine, 2020).

The most significantly altered pathways in PreE cases with adverse neonatal outcomes included sphingolipid and methylhistidine metabolisms, and phosphatidylethanolamine biosynthesis. Sphingolipids are emerging as key regulators of vascular function and are altered in metabolic disorders and cardiovascular diseases. Previous studies identified sphingomyelins and ceramides as markers for PreE and endothelial cell dysfunction, (Dobierzewska et al., 2017) itself a suspected pathogenic mechanism of the disorder. These alterations in sphingolipids are likely attributable to inflammatory processes through induction of the COX-2 pathway (Nixon, 2009). Recent data also showed alterations of sphingolipid metabolism in the feto-placental vasculature in cases with PreE where the sphingolipid biosynthesis is shifted towards sphingomyelin production (Del Gaudio et al., 2020). Increased phosphatidylethanolamine levels have been linked to other inflammatory conditions such as obesity, metabolic syndrome, heart disease, amongst others (Calzada et al., 2016). Lastly, we found altered methylhistidine levels in those PreE cases with adverse neonatal outcomes. Increased levels of histidine have been hypothesized to reflect high oxidative stress in PreE (Youssef et al., 2020, 2022). Methylhistidine through its role in oxidative stress, has been implicated in perinatal asphyxia and hypoxic ischemic encephalopathy (Valerio et al., 2022). Most of the severe neonatal complications in PreE are linked to prematurity which is itself associated with placental inflammation and vascular dysfunction (Kim et al., 2015).

Our study has several strengths. We address an important clinical need, the prenatal prediction of cases that are likely to experience severe complications. This may facilitate patient counseling, closer surveillance and optimization of care as noted previously. The prospect of comprehensive laboratory categorization of patients into risk groups rather than current heavy reliance on a combination of subjective symptoms and variable clinical findings is a potentially promising one. Further, better understanding of the molecular basis of PreE is important to the development of targeted therapy. For example, we found substantial evidence of lipid disturbance in the development of adverse outcome in PreE. This appears to justify recent experimental focus on the use of anti-lipid agents to mitigate PreE severity even if disease prevention cannot be achieved. There are additional important clinical implications of our study. Late-onset PreE is a significantly more common, but milder than the early-onset group. There is however some overlap between these two groups. The separation of the groups based on a 34 weeks gestation threshold for presentation is largely arbitrary as cases may develop PreE before 34 weeks which is not recognized until an office visit after that gestational threshold. By identifying late-onset cases that are at risk for severe complications, metabolomics has the potential to segregate those cases that warrant closer surveillance and more aggressive therapy. In this study, we performed deep metabolomic analysis generating a large number of metabolites for analysis and yielding increased resolution of the metabolomic processes involved in maternal and newborn complications. Finally, the prospective nature of our recruitment helped to attenuate study bias.

This study is not without limitations. Our sample size was modest. We were unable to evaluate the performance of these algorithms in an independent test group. To counter the sample size limitations and enhance the robustness and generalizability of our results tenfold CV technique was employed to validate regression models. Validation of the metabolite models reported herein is warranted for these markers to become a clinically available tool. The fasting times prior to sampling was at least 4 h, however, individual participant data for the duration was not available. Our study did not compare currently available laboratory markers of disease severity (hematocrit, platelet count, transaminase levels, creatinine) to metabolomic markers. First, these laboratory drawings were not all performed on the same day of metabolomics sampling. Secondly, most patients with adverse outcome do not develop clinical laboratory changes (Cantu et al., 2014). Hence, the predictive value of such markers may fail to identify many cases with adverse outcomes. In addition, we were not able to directly compare angiogenesis markers such as sFlt-1 and PlGF to metabolites for prediction of complications as these are not yet widely available in the USA.

In conclusion, we report the investigation of the metabolomic basis of severe maternal and newborn complications of PreE and developed metabolomic models for the prediction of such outcomes. We found that the dysregulation of major lipid pathways are associated with the development of adverse outcomes in PreE. Our metabolomic models demonstrated good predictive accuracy for adverse clinical outcomes. Our findings could potentially help to address an important clinical need for objective biomarkers for PreE severity. For the future, understanding the pathogenesis of maternal and newborn complications of PreE could facilitate the development targeted therapeutics to attenuate or obviate these outcomes. Larger studies with an independent test or validation group are now warranted based on our findings.

### Supplementary Information

Below is the link to the electronic supplementary material.

Supplementary file1 (DOCX 160 KB)

Supplementary file2 (DOCX 221 KB)

Supplementary file3 (DOCX 27 KB)

## Data Availability

The datasets generated during the current study are available from the corresponding author on reasonable request.
