# Supplementary material for: Metabolomic prediction of severe maternal and newborn complications in preeclampsia
Source: Metabolomics. 2024 May 18;20(3):56. doi: 10.1007/s11306-024-02123-0 (PMC11102370; doi:10.1007/s11306-024-02123-0)
Supplement: Supplementary file 1 — Supplementary file1 (DOCX 160 KB) [file 11306_2024_2123_MOESM1_ESM.docx]

**Supplementary Results**

**Supplemental table S1. Comparison of demographic and clinical characteristics: Early-onset preeclampsia vs Late-onset preeclampsia**

| **Parameter** | **Early-onset PreE** | **Late-onset PreE** | ***p*-value** |
| --- | --- | --- | --- |
| **Number of patients** | 36 | 40 | N/A |
| **Age, years, mean (SD)** | 30.4 (5.04) | 30.38 (5.35) | 0.899^t^ |
| **Race** | Caucasian (N=23)  Black (N=8)  Asian (N=4)  Hispanic (N=0)  Other (N=1) | Caucasian (N=29)  Black (N=8)  Asian (N=2)  Hispanic (N=1)  Other (N=0) | 0.532^c^ |
| **Nulliparous, n (%)** | 19 (52.8%) | 29 (72.5%) | 0.075^c^ |
| **BMI, mean (SD)** | 35.97 (9.25) | 34.28 (6.45) | 0.072^t^ |
| **Gestational age at the time of collection** | 30.49 (2.59) | 35.96 (3.26) | 0.692^t^ |
| **Gestational age at delivery, mean (SD)** | 31.78 (2.79) | 37.45 (1.77) | **<0.001^u^** |
| **GDM** | 4 (11.1%) | 3 (7.5%) | 0.587^c^ |
| **cHTN** | 14 (38.9%) | 10 (25.0%) | 0.193^c^ |
| **Pregestational Diabetes** | 3 (8.3%) | 8 (20.0%) | 0.149^c^ |
| **Fetal Growth Restriction** | 17 (47.2%) | 6 (15.0%) | **0.002^c^** |
| **History of Preeclampsia** | 7 (19.4%) | 6 (15.0%) | 0.607^c^ |
| **IVF** | 4 (11.1%) | 5 (12.5%) | 0.852^c^ |
| **Mode of delivery (Normal spontaneous vaginal delivery / Cesarean section)** | SVD 8 (22.2%)  C/S 28 (77.8%) | SVD 24 (60.0%)  C/S 16 (40.0%) | **<0.001^c^** |
| **Postpartum Hemorrhage** | 2 (5.6%) | 3 (7.5%) | 0.733^c^ |
| **Newborn weight (g), mean (SD)** | 1627.66 (693.6) | 3034.93  (680.04) | 0.425^t^ |
| **SGA at birth** | 22 (62.9%) | 8 (20.0%) | **<0.001^t^** |
| **Apgar’s 5 min, mean (SD)** | 8.49 (0.78) | 8.75 (0.56) | 0.083^u^ |

t= Independent sample t-test

c= Pearson chi-squared

u= Mann. Whitney U test**Supplemental table S2. Comparison of demographic and clinical characteristics: Early-onset Preeclampsia vs controls**

| **Parameter** | **Early-Onset Preeclampsia** | **Controls** | **p-value** |
| --- | --- | --- | --- |
| **Number of patients** | 36 | 40 | N/A |
| **Age, years, mean (SD)** | 30.4 (5.04) | 30.5 (6.21) | 0.925 ^u^ |
| **Race** | Caucasian (N=23)  Black (N=8)  Asian (N=4)  Hispanic (N=0)  Other (N=1) | Caucasian (N=33)  Black (N=5)  Asian (N=1)  Hispanic (N=0)  Other (N=1) | 0.121 ^c^ |
| **Nulliparous, n (%)** | 19 (52.8%) | 12 (30.0%) | **0.044 ^c^** |
| **BMI, mean (SD)** | 35.97 (9.25) | 31.24 (5.88) | **0.017 ^t^** |
| **Gestational age at the time of collection** | 30.49 (2.59) | 36.20 (3.28) | **<0.001^u^** |
| **Gestational age at delivery, mean (SD)** | 31.78 (2.79) | 39.11 (1.09) | **<0.001^u^** |
| **GDM** | 4 (11.1%) | 0 (0.0%) | 0.30^c^ |
| **cHTN** | 14 (38.9%) | 0 (0.0%) | **<0.001^c^** |
| **Pregestational Diabetes** | 3 (8.3%) | 0 (0.0%) | 0.062^c^ |
| **Fetal Growth Restriction** | 17 (47.2%) | 0 (0.0%) | **<0.001^c^** |
| **History of Preeclampsia** | 7 (19.4%) | 1 (2.5%) | **0.016^c^** |
| **IVF** | 4 (11.1%) | 1 (2.5%) | 0.131^c^ |
| **Mode of delivery (Normal spontaneous vaginal delivery / Cesarean section)** | SVD 8 (22.2%)  C/S 28 (77.8%) | SVD 26 (65.0%)  C/S 14 (35.0%) | **<0.001^c^** |
| **Postpartum Hemorrhage** | 2 (5.6%) | 2 (5.0%) | 0.914^c^ |
| **Newborn weight (g), mean (SD)** | 1627.66 (693.6) | 3364.90 (320.85) | **<0.001^u^** |
| **SGA at birth** | 22 (62.9%) | 1 (2.5%) | **< 0.001^c^** |
| **Apgar’s 5 min, mean (SD)** | 8.49 (0.78) | 8.88 (0.56) | **<0.001^u^** |

t= Independent sample t-test

c= Pearson chi-squared

u= Mann. Whitney U test

**Supplemental table S3. Comparison of demographic and clinical characteristics: Late-onset preeclampsia vs controls**

| **Parameter** | **Late-Onset Preeclampsia** | **Controls** | **p-value** |
| --- | --- | --- | --- |
| **Number of patients** | 40 | 40 | N/A |
| **Age, years, mean (SD)** | 30.38 (5.35) | 30.5 (6.21) | 0.198^t^ |
| **Race** | Caucasian (N=29)  Black (N=8)  Asian (N=2)  Hispanic (N=1)  Other (N=0) | Caucasian (N=33)  Black (N=5)  Asian (N=1)  Hispanic (N=0)  Other (N=1) | 0.311^c^ |
| **Nulliparous, n (%)** | 29 (72.5%) | 12 (30.0%) | **<0.001^c^** |
| **BMI, mean (SD)** | 34.28 (6.45) | 31.24 (5.88) | 0.416^t^ |
| **Gestational age at the time of collection** | 35.96 (3.26) | 36.20 (3.28) | 0.973^u^ |
| **Gestational age at delivery, mean (SD)** | 37.45 (1.77) | 39.11 (1.09) | **<0.001^u^** |
| **GDM** | 3 (7.5%) | 0 (0.0%) | 0.077^c^ |
| **cHTN** | 10 (25.0%) | 0 (0.0%) | **<0.001^c^** |
| **Pregestational Diabetes** | 8 (20.0%) | 0 (0.0%) | **0.003^c^** |
| **Fetal Growth Restriction (n)** | 6 (15.0%) | 0 (0.0%) | **0.011^c^** |
| **History of Preeclampsia (n)** | 6 (15.0%) | 1 (2.5%) | **0.048^c^** |
| **IVF** | 5 (12.5%) | 1 (2.5%) | 0.090^c^ |
| **Mode of delivery (Normal spontaneous vaginal delivery / Cesarean section)** | SVD 24 (60.0%)  C/S 16 (40.0%) | SVD 26 (65.0%)  C/S 14 (35.0%) | 0.644^c^ |
| **Postpartum Hemorrhage (n)** | 3 (7.5%) | 2 (5.0%) | 0.644^c^ |
| **Newborn weight (g), mean (SD)** | 3034.93  (680.04) | 3364.90 (320.85) | **<0.001^t^** |
| **SGA at birth (n)** | 8 (20.0%) | 1 (2.5%) | **0.013^c^** |
| **Apgar’s 5 min, mean (SD)** | 8.75 (0.56) | 8.88 (0.56) | **0.034^u^** |

t= Independent sample t-test

c= Pearson chi-squared

u= Mann. Whitney U test

**Supplemental table S4. Metabolites / Lipids identified by LC-MS and NMR**

| **LC-MS** | | | | | | **NMR** |
| --- | --- | --- | --- | --- | --- | --- |
| C0 Carnitine | DG(16:0_20:3) | PC aa C42:6 | SM C26:0 | TG(18:0_32:0) | TG(20:1_34:2) | 1-Methylhistidine |
| C2 Acetylcarnitine | DG(16:0_20:4) | PC ae C30:0 | SM C26:1 | TG(18:0_32:1) | TG(20:1_34:3) | 2-Hydroxybutyric acid |
| C3 Propionylcarnitine | DG(16:1_18:0) | PC ae C30:1 | H1 Hexoses | TG(18:0_32:2) | TG(20:2_32:0) | Acetic acid |
| C3-DC (C4-OH) Malonylcarnitine (Hydroxybutyrylcarnitine) | DG(16:1_18:1) | PC ae C30:2 | TG(14:0_32:2) | TG(18:0_34:2) | TG(20:2_32:1) | Betaine |
| C3-OH Hydroxypropionylcarnitine | DG(16:1_18:2) | PC ae C32:1 | TG(14:0_34:0) | TG(18:0_34:3) | TG(20:2_34:1) | Acetoacetate |
| C3:1 Propenoylcarnitine | DG(16:1_20:0) | PC ae C32:2 | TG(14:0_34:1) | TG(18:0_36:1) | TG(20:2_34:2) | Carnitine |
| C4 Butyrylcarnitine | DG(17:0_17:1) | PC ae C34:0 | TG(14:0_34:2) | TG(18:0_36:2) | TG(20:2_34:3) | Creatine |
| C4:1 Butenylcarnitine | DG(17:0_18:1) | PC ae C34:1 | TG(14:0_34:3) | TG(18:0_36:3) | TG(20:2_34:4) | Citric acid |
| C5 Valerylcarnitine | DG(18:0_20:4) | PC ae C34:2 | TG(14:0_35:1) | TG(18:0_36:4) | TG(20:2_36:5) | Choline |
| C5-DC (C6-OH) Glutarylcarnitine (Hydroxyhexanoylcarnitine) | DG(18:1_18:1) | PC ae C34:3 | TG(14:0_35:2) | TG(18:0_36:5) | TG(20:3_32:0) | Ethanol |
| C5-M-DC Methylglutarylcarnitine | DG(18:1_18:2) | PC ae C36:0 | TG(14:0_36:1) | TG(18:0_38:6) | TG(20:3_32:1) | D-Glucose |
| C5-OH (C3-DC-M) Hydroxyvalerylcarnitine (Methylmalonylcarnitine) | DG(18:1_18:3) | PC ae C36:1 | TG(14:0_36:2) | TG(18:0_38:7) | TG(20:3_32:2) | Gly |
| C5:1 Tiglylcarnitine | DG(18:1_18:4) | PC ae C36:2 | TG(14:0_36:3) | TG(18:1_26:0) | TG(20:3_34:0) | Glycerol |
| C5:1-DC Glutaconylcarnitine | DG(18:1_20:0) | PC ae C36:3 | TG(14:0_36:4) | TG(18:1_28:1) | TG(20:3_34:1) | Formate |
| C6 (C4:1-DC) Hexanoylcarnitine (Fumarylcarnitine) | DG(18:1_20:1) | PC ae C36:4 | TG(14:0_38:4) | TG(18:1_30:0) | TG(20:3_34:2) | Hypoxanthine |
| C6:1 Hexenoylcarnitine | DG(18:1_20:2) | PC ae C36:5 | TG(14:0_38:5) | TG(18:1_30:1) | TG(20:3_34:3) | L-Phenylalanine |
| C7-DC Pimeloylcarnitine | DG(18:1_20:3) | PC ae C38:0 | TG(16:0_28:1) | TG(18:1_30:2) | TG(20:3_36:3) | Ala |
| C8 Octanoylcarnitine | DG(18:1_20:4) | PC ae C38:1 | TG(16:0_28:2) | TG(18:1_31:0) | TG(20:3_36:4) | Ile |
| C9 Nonaylcarnitine | DG(18:1_22:5) | PC ae C38:2 | TG(16:0_30:2) | TG(18:1_32:0) | TG(20:3_36:5) | Lys |
| C10 Decanoylcarnitine | DG(18:1_22:6) | PC ae C38:3 | TG(16:0_32:0) | TG(18:1_32:1) | TG(20:4_30:0) | L-Lactic acid |
| C10:1 Decenoylcarnitine | DG(18:2_18:2) | PC ae C38:4 | TG(16:0_32:1) | TG(18:1_32:2) | TG(20:4_32:0) | Asp |
| C10:2 Decadienoylcarnitine | DG(18:2_18:3) | PC ae C38:5 | TG(16:0_32:2) | TG(18:1_32:3) | TG(20:4_32:1) | Pyruvic acid |
| C12 Dodecanoylcarnitine | DG(18:2_18:4) | PC ae C38:6 | TG(16:0_32:3) | TG(18:1_33:0) | TG(20:4_32:2) | Succinate |
| C12-DC Dodecanedioylcarnitine | DG(18:2_20:0) | PC ae C40:1 | TG(16:0_33:1) | TG(18:1_33:1) | TG(20:4_33:2) | Urea |
| C12:1 Dodecenoylcarnitine | DG(18:2_20:4) | PC ae C40:2 | TG(16:0_33:2) | TG(18:1_33:2) | TG(20:4_34:0) | 3-Hydroxybutyric acid |
| C14 Tetradecanoylcarnitine | DG(18:3_18:3) | PC ae C40:3 | TG(16:0_34:0) | TG(18:1_33:3) | TG(20:4_34:1) | Creatinine |
| C14:1 Tetradecenoylcarnitine | DG(22:1_22:2) | PC ae C40:4 | TG(16:0_34:1) | TG(18:1_34:1) | TG(20:4_34:2) | Malonate |
| C14:1-OH Hydroxytetradecenoylcarnitine | DG-O(16:0_18:1) | PC ae C40:5 | TG(16:0_34:2) | TG(18:1_34:2) | TG(20:4_34:3) | Methionine |
| C14:2 Tetradecadienoylcarnitine | DG-O(16:0_20:4) | PC ae C40:6 | TG(16:0_34:3) | TG(18:1_34:3) | TG(20:4_35:3) | Isopropyl alcohol |
| C14:2-OH Hydroxytetradecadienoylcarnitine | Cer(d18:0/18:0(OH)) | PC ae C42:1 | TG(16:0_34:4) | TG(18:1_34:4) | TG(20:4_36:2) | Val |
| C16 Hexadecanoylcarnitine | Cer(d18:0/18:0) | PC ae C42:2 | TG(16:0_35:1) | TG(18:1_35:2) | TG(20:4_36:3) | Acetone |
| C16-OH Hydroxyhexadecanoylcarnitine | Cer(d18:0/20:0) | PC ae C42:3 | TG(16:0_35:2) | TG(18:1_35:3) | TG(20:4_36:4) | Isobutyric acid |
| C16:1 Hexadecenoylcarnitine | Cer(d18:0/22:0) | PC ae C42:4 | TG(16:0_35:3) | TG(18:1_36:0) | TG(20:4_36:5) | Methanol |
| C16:1-OH Hydroxyhexadecenoylcarnitine | Cer(d18:0/24:0) | PC ae C42:5 | TG(16:0_36:2) | TG(18:1_36:1) | TG(20:5_34:0) | Propylene glycol |
| C16:2 Hexadecadienoylcarnitine | Cer(d18:0/24:1) | PC ae C44:3 | TG(16:0_36:3) | TG(18:1_36:2) | TG(20:5_34:1) | Dimethyl sulfone |
| C16:2-OH Hydroxyhexadecadienoylcarnitine | Cer(d18:0/26:1(OH)) | PC ae C44:4 | TG(16:0_36:4) | TG(18:1_36:3) | TG(20:5_34:2) | Trigonelline |
| C18 Octadecanoylcarnitine | Cer(d18:0/26:1) | PC ae C44:5 | TG(16:0_36:5) | TG(18:1_36:4) | TG(20:5_36:2) | TMAO |
| C18:1 Octadecenoylcarnitine | AA | PC ae C44:6 | TG(16:0_36:6) | TG(18:1_36:5) | TG(20:5_36:3) | Arg |
| C18:1-OH Hydroxyoctadecenoylcarnitine | DHA | Hex2Cer(d18:1/14:0) | TG(16:0_37:3) | TG(18:1_36:6) | TG(22:0_32:4) | Asn |
| C18:2 Octadecadienylcarnitine | EPA | Hex2Cer(d18:1/16:0) | TG(16:0_38:1) | TG(18:1_38:5) | TG(22:1_32:5) | Cys |
| Cer(d18:1/14:0) | FA(18:1) | Hex2Cer(d18:1/18:0) | TG(16:0_38:2) | TG(18:1_38:6) | TG(22:2_32:4) | Gln |
| Cer(d18:1/16:0) | FA(18:2) | Hex2Cer(d18:1/20:0) | TG(16:0_38:3) | TG(18:1_38:7) | TG(22:3_30:2) | Glu |
| Cer(d18:1/18:0(OH)) | FA(20:1) | Hex2Cer(d18:1/22:0) | TG(16:0_38:4) | TG(18:2_28:0) | TG(22:4_32:0) | His |
| Cer(d18:1/18:0) | FA(20:2) | Hex2Cer(d18:1/24:0) | TG(16:0_38:5) | TG(18:2_30:0) | TG(22:4_32:2) | Leu |
| Cer(d18:1/18:1) | FA(20:3) | Hex2Cer(d18:1/24:1) | TG(16:0_38:6) | TG(18:2_30:1) | TG(22:4_34:2) | Met |
| Cer(d18:1/20:0(OH)) | lysoPC a C16:0 | Hex2Cer(d18:1/26:0) | TG(16:0_38:7) | TG(18:2_31:0) | TG(22:5_32:0) | Pro |
| Cer(d18:1/20:0) | lysoPC a C16:1 | Hex2Cer(d18:1/26:1) | TG(16:0_40:6) | TG(18:2_32:0) | TG(22:5_32:1) | Ser |
| Cer(d18:1/22:0) | lysoPC a C17:0 | Hex3Cer(d18:1/16:0) | TG(16:0_40:7) | TG(18:2_32:1) | TG(22:5_34:1) | Thr |
| Cer(d18:1/23:0) | lysoPC a C18:0 | Hex3Cer(d18:1/18:0) | TG(16:0_40:8) | TG(18:2_32:2) | TG(22:5_34:2) | Trp |
| Cer(d18:1/24:0) | lysoPC a C18:1 | Hex3Cer(d18:1/24:1) | TG(16:1_28:0) | TG(18:2_33:0) | TG(22:5_34:3) | Tyr |
| Cer(d18:1/24:1) | lysoPC a C18:2 | Hex3Cer(d18:1/26:1) | TG(16:1_30:1) | TG(18:2_33:1) | TG(22:6_32:0) | 1-Met-His |
| Cer(d18:1/25:0) | lysoPC a C20:3 | Hex3Cer(d18:1_20:0) | TG(16:1_32:0) | TG(18:2_33:2) | TG(22:6_32:1) | 3-Met-His |
| Cer(d18:1/26:0) | lysoPC a C20:4 | Hex3Cer(d18:1_22:0) | TG(16:1_32:1) | TG(18:2_34:0) | TG(22:6_34:1) | AABA |
| Cer(d18:1/26:1) | lysoPC a C24:0 | HexCer(d16:1/22:0) | TG(16:1_32:2) | TG(18:2_34:1) | TG(22:6_34:2) | ADMA |
| Cer(d18:2/14:0) | lysoPC a C26:0 | HexCer(d16:1/24:0) | TG(16:1_33:1) | TG(18:2_34:2) | TG(22:6_34:3) | alpha-AAA |
| Cer(d18:2/16:0) | lysoPC a C26:1 | HexCer(d18:1/14:0) | TG(16:1_34:0) | TG(18:2_34:3) | Choline | BABA |
| Cer(d18:2/18:0) | lysoPC a C28:0 | HexCer(d18:1/16:0) | TG(16:1_34:1) | TG(18:2_34:4) |  | Betaine |
| Cer(d18:2/18:1) | lysoPC a C28:1 | HexCer(d18:1/18:0) | TG(16:1_34:2) | TG(18:2_35:1) |  | Cit |
| Cer(d18:2/20:0) | PC aa C28:1 | HexCer(d18:1/18:1) | TG(16:1_34:3) | TG(18:2_35:2) |  | Creatinine |
| Cer(d18:2/22:0) | PC aa C30:0 | HexCer(d18:1/20:0) | TG(16:1_36:1) | TG(18:2_35:3) |  | HArg |
| Cer(d18:2/23:0) | PC aa C30:2 | HexCer(d18:1/22:0) | TG(16:1_36:2) | TG(18:2_36:0) |  | HCys |
| Cer(d18:2/24:0) | PC aa C32:0 | HexCer(d18:1/23:0) | TG(16:1_36:3) | TG(18:2_36:1) |  | Kynurenine |
| Cer(d18:2/24:1) | PC aa C32:1 | HexCer(d18:1/24:0) | TG(16:1_36:4) | TG(18:2_36:2) |  | Met-SO |
| CE(14:0) | PC aa C32:2 | HexCer(d18:1/24:1) | TG(16:1_36:5) | TG(18:2_36:3) |  | Orn |
| CE(14:1) | PC aa C32:3 | HexCer(d18:1/26:0) | TG(16:1_38:3) | TG(18:2_36:4) |  | ProBetaine |
| CE(15:0) | PC aa C34:1 | HexCer(d18:1/26:1) | TG(16:1_38:4) | TG(18:2_36:5) |  | Sarcosine |
| CE(15:1) | PC aa C34:2 | HexCer(d18:2/16:0) | TG(16:1_38:5) | TG(18:2_38:4) |  | SDMA |
| CE(16:0) | PC aa C34:3 | HexCer(d18:2/18:0) | TG(17:0_32:1) | TG(18:2_38:5) |  | t4-OH-Pro |
| CE(16:1) | PC aa C34:4 | HexCer(d18:2/20:0) | TG(17:0_34:1) | TG(18:2_38:6) |  | Taurine |
| CE(17:0) | PC aa C36:0 | HexCer(d18:2/22:0) | TG(17:0_34:2) | TG(18:3_30:0) |  | TrpBetaine |
| CE(17:1) | PC aa C36:1 | HexCer(d18:2/23:0) | TG(17:0_34:3) | TG(18:3_32:0) |  | CA |
| CE(18:0) | PC aa C36:2 | HexCer(d18:2/24:0) | TG(17:0_36:3) | TG(18:3_32:1) |  | CDCA |
| CE(18:1) | PC aa C36:3 | Cortisol | TG(17:0_36:4) | TG(18:3_33:2) |  | DCA |
| CE(18:2) | PC aa C36:4 | Cortisone | TG(17:1_32:1) | TG(18:3_34:0) |  | GCA |
| CE(18:3) | PC aa C36:5 | DHEAS | TG(17:1_34:1) | TG(18:3_34:1) |  | GCDCA |
| CE(20:0) | PC aa C36:6 | 3-IAA | TG(17:1_34:2) | TG(18:3_34:2) |  | GDCA |
| CE(20:1) | PC aa C38:0 | 3-IPA | TG(17:1_34:3) | TG(18:3_34:3) |  | GLCA |
| CE(20:3) | PC aa C38:1 | Ind-SO4 | TG(17:1_36:3) | TG(18:3_35:2) |  | GLCAS |
| CE(20:4) | PC aa C38:3 | Hypoxanthine | TG(17:1_36:4) | TG(18:3_36:1) |  | GUDCA |
| CE(20:5) | PC aa C38:4 | Xanthine | TG(17:1_36:5) | TG(18:3_36:2) |  | TCA |
| CE(22:0) | PC aa C38:5 | SM (OH) C14:1 | TG(17:1_38:5) | TG(18:3_36:3) |  | TCDCA |
| CE(22:1) | PC aa C38:6 | SM (OH) C16:1 | TG(17:1_38:6) | TG(18:3_36:4) |  | TDCA |
| CE(22:2) | PC aa C40:1 | SM (OH) C22:1 | TG(17:1_38:7) | TG(18:3_38:5) |  | TLCA |
| CE(22:5) | PC aa C40:2 | SM (OH) C22:2 | TG(17:2_34:2) | TG(18:3_38:6) |  | TMCA |
| CE(22:6) | PC aa C40:3 | SM (OH) C24:1 | TG(17:2_34:3) | TG(20:0_32:3) |  | beta-Ala |
| p-Cresol-SO4 | PC aa C40:4 | SM C16:0 | TG(17:2_36:2) | TG(20:0_32:4) |  | GABA |
| DG(14:0_20:0) | PC aa C40:5 | SM C16:1 | TG(17:2_36:3) | TG(20:0_34:1) |  | Histamine |
| DG(14:1_18:1) | PC aa C40:6 | SM C18:0 | TG(17:2_36:4) | TG(20:1_30:1) |  | Serotonin |
| DG(14:1_20:2) | PC aa C42:0 | SM C18:1 | TG(17:2_38:5) | TG(20:1_32:1) |  | AconAcid |
| DG(16:0_16:1) | PC aa C42:1 | SM C20:2 | TG(17:2_38:6) | TG(20:1_32:2) |  | DiCA(12:0) |
| DG(16:0_18:1) | PC aa C42:2 | SM C22:3 | TG(17:2_38:7) | TG(20:1_32:3) |  | DiCA(14:0) |
| DG(16:0_18:2) | PC aa C42:4 | SM C24:0 | TG(18:0_30:0) | TG(20:1_34:0) |  | HipAcid |
| DG(16:0_20:0) | PC aa C42:5 | SM C24:1 | TG(18:0_30:1) | TG(20:1_34:1) |  | Lac |

**Supplemental table S5. Significantly different metabolites and lipids between preeclampsia cases with and without maternal adverse outcome (p<0.05)**

| Name | Mean (SD) of Controls | Mean (SD) of Cases | Fold Change | Cases vs Controls | **p-value** |
| --- | --- | --- | --- | --- | --- |
| Carnitine | 9.080 (7.820) | 16.895 (15.078) | 1.86 | Up | **0.00056** |
| Glycerol | 133.621 (42.363) | 175.860 (44.779) | 1.32 | Up | **0.00071** |
| lysoPC a C18:2 | 9.539 (4.612) | 13.868 (5.658) | 1.45 | Up | **0.00125** |
| Met-SO | 0.195 (0.229) | 0.589 (0.684) | 3.03 | Up | **0.00134** |
| lysoPC a C17:0 | 0.575 (0.217) | 0.779 (0.283) | 1.35 | Up | **0.00137** |
| Succinate | 12.146 (5.920) | 17.530 (8.018) | 1.44 | Up | **0.00199** |
| lysoPC a C18:0 | 9.481 (3.584) | 13.362 (5.426) | 1.41 | Up | **0.00464** |
| Creatine | 23.541 (13.123) | 34.960 (16.663) | 1.49 | Up | **0.00643** |
| Methionine | 14.354 (6.091) | 18.905 (7.419) | 1.32 | Up | **0.00846** |
| Asn | 46.374 (16.003) | 58.055 (17.924) | 1.25 | Up | **0.00898** |
| 3-Hydroxybutyric acid | 188.061 (409.657) | 40.600 (78.070) | -4.63 | Down | **0.00929** |
| Met | 22.051 (7.166) | 27.000 (8.648) | 1.22 | Up | **0.01198** |
| HexCer(d18:1/26:1) | 0.099 (0.029) | 0.118 (0.028) | 1.19 | Up | **0.01235** |
| CA | 0.123 (0.178) | 0.033 (0.040) | -3.75 | Down | **0.01328** |
| PC ae C44:3 | 0.145 (0.047) | 0.174 (0.034) | 1.2 | Up | **0.01476** |
| lysoPC a C16:0 | 43.422 (16.128) | 60.030 (26.246) | 1.38 | Up | **0.01534** |
| Hex3Cer(d18:1_20:0) | 0.082 (0.025) | 0.100 (0.031) | 1.21 | Up | **0.01603** |
| Dimethyl sulfone | 6.998 (27.440) | 4.820 (4.034) | -1.45 | Down | **0.01607** |
| Hex2Cer(d18:1/22:0) | 0.181 (0.053) | 0.214 (0.049) | 1.18 | Up | **0.01777** |
| lysoPC a C18:1 | 6.004 (2.595) | 8.399 (3.911) | 1.4 | Up | **0.0186** |
| PC ae C44:6 | 1.428 (0.463) | 1.706 (0.423) | 1.19 | Up | **0.02102** |
| Lys | 158.439 (49.062) | 190.485 (60.796) | 1.2 | Up | **0.02139** |
| PC aa C42:2 | 0.196 (0.054) | 0.226 (0.040) | 1.16 | Up | **0.02183** |
| Hex3Cer(d18:1_22:0) | 0.237 (0.079) | 0.288 (0.098) | 1.22 | Up | **0.0232** |
| PC aa C42:1 | 0.332 (0.119) | 0.399 (0.094) | 1.2 | Up | **0.02615** |
| Sarcosine | 0.552 (0.538) | 0.873 (0.960) | 1.58 | Up | **0.02649** |
| HCys | 5.049 (2.615) | 6.019 (2.323) | 1.19 | Up | **0.02657** |
| PC aa C42:0 | 0.645 (0.220) | 0.767 (0.171) | 1.19 | Up | **0.02774** |
| lysoPC a C16:1 | 0.918 (0.463) | 1.396 (0.916) | 1.52 | Up | **0.03421** |
| SM C26:1 | 0.243 (0.074) | 0.276 (0.065) | 1.14 | Up | **0.03471** |
| L-Phenylalanine | 33.639 (13.266) | 40.930 (12.507) | 1.22 | Up | **0.03561** |
| Cer(d18:1/14:0) | 0.076 (0.027) | 0.091 (0.025) | 1.19 | Up | **0.0372** |
| Pyruvic acid | 85.559 (51.125) | 116.645 (83.655) | 1.36 | Up | **0.03843** |
| CE(20:1) | 1.408 (1.105) | 1.567 (0.499) | 1.11 | Up | **0.03952** |
| Creatinine | 28.163 (11.513) | 35.655 (13.476) | 1.27 | Up | **0.04185** |
| C18 | 0.029 (0.023) | 0.038 (0.022) | 1.33 | Up | **0.04211** |
| Acetoacetate | 64.071 (115.499) | 30.795 (46.695) | -2.08 | Down | **0.04246** |
| CE(16:0) | 305.036 (73.137) | 344.450 (76.371) | 1.13 | Up | **0.04439** |
| Gly | 167.331 (53.669) | 195.800 (57.192) | 1.17 | Up | **0.04489** |
| PC ae C42:1 | 0.393 (0.106) | 0.445 (0.076) | 1.13 | Up | **0.04517** |
| HexCer(d16:1/22:0) | 0.224 (0.097) | 0.260 (0.084) | 1.16 | Up | **0.04619** |
| Choline | 3.685 (1.598) | 5.065 (2.915) | 1.37 | Up | **0.04677** |
| Cortisone | 0.098 (0.057) | 0.073 (0.070) | -1.35 | Down | **0.04677** |

**Supplemental table S6. Significantly different metabolites and lipids between preeclampsia cases with and without neonatal adverse outcome (p<0.05)**

| Name | Mean (SD) of Controls | Mean (SD) of Cases | Fold Change | Cases vs Controls | **p-value** |
| --- | --- | --- | --- | --- | --- |
| lysoPC a C17:0 | 0.545 (0.214) | 0.779 (0.246) | 1.43 | Up | **0.00006** |
| lysoPC a C16:1 | 0.856 (0.480) | 1.385 (0.765) | 1.62 | Up | **0.00008** |
| lysoPC a C18:1 | 5.672 (2.390) | 8.382 (3.634) | 1.48 | Up | **0.00016** |
| TG(16:1_30:1) | 3.038 (3.351) | 5.687 (4.211) | 1.87 | Up | **0.00023** |
| TG(16:1_28:0) | 1.817 (1.939) | 3.928 (3.054) | 2.16 | Up | **0.00031** |
| TG(16:0_32:3) | 6.957 (4.746) | 10.923 (4.912) | 1.57 | Up | **0.00033** |
| TG(16:1_32:2) | 7.802 (7.459) | 11.313 (5.375) | 1.45 | Up | **0.00034** |
| lysoPC a C16:0 | 41.913 (16.834) | 58.463 (22.436) | 1.39 | Up | **0.00039** |
| lysoPC a C20:3 | 0.814 (0.433) | 1.223 (0.580) | 1.5 | Up | **0.00044** |
| TG(18:3_30:0) | 3.345 (2.797) | 5.403 (2.852) | 1.62 | Up | **0.00049** |
| TG(16:1_33:1) | 3.320 (2.596) | 4.940 (2.242) | 1.49 | Up | **0.00051** |
| TG(17:0_32:1) | 2.356 (1.625) | 3.714 (1.918) | 1.58 | Up | **0.00051** |
| TG(16:0_28:1) | 5.045 (5.889) | 9.826 (8.047) | 1.95 | Up | **0.0006** |
| TG(17:0_34:3) | 1.410 (0.760) | 1.861 (0.570) | 1.32 | Up | **0.00064** |
| TG(18:2_31:0) | 4.650 (2.455) | 6.425 (2.138) | 1.38 | Up | **0.00065** |
| TG(16:0_32:2) | 54.306 (36.778) | 83.474 (37.970) | 1.54 | Up | **0.00067** |
| TG(14:0_35:1) | 1.427 (1.213) | 2.157 (1.077) | 1.51 | Up | **0.00069** |
| TG(14:0_38:5) | 1.129 (0.710) | 1.719 (0.777) | 1.52 | Up | **0.00069** |
| PC aa C36:6 | 0.997 (0.500) | 1.478 (0.682) | 1.48 | Up | **0.00075** |
| TG(16:0_33:2) | 7.318 (4.240) | 10.061 (3.225) | 1.37 | Up | **0.00078** |
| TG(20:3_32:2) | 1.052 (0.658) | 1.491 (0.596) | 1.42 | Up | **0.00081** |
| lysoPC a C18:0 | 9.270 (3.776) | 12.740 (4.778) | 1.37 | Up | **0.00085** |
| TG(20:5_34:2) | 1.776 (1.043) | 2.721 (1.263) | 1.53 | Up | **0.00086** |
| TG(18:2_30:0) | 38.865 (24.494) | 54.630 (20.498) | 1.41 | Up | **0.00091** |
| lysoPC a C18:2 | 9.291 (4.168) | 13.196 (6.061) | 1.42 | Up | **0.00097** |
| TG(16:0_33:1) | 14.760 (8.879) | 21.370 (9.296) | 1.45 | Up | **0.00099** |
| TG(16:0_34:4) | 7.506 (4.399) | 10.663 (3.750) | 1.42 | Up | **0.00099** |
| TG(18:1_31:0) | 6.323 (3.370) | 8.979 (3.561) | 1.42 | Up | **0.00099** |
| TG(20:4_30:0) | 2.498 (1.867) | 4.716 (3.309) | 1.89 | Up | **0.00099** |
| TG(20:5_34:0) | 0.462 (0.283) | 0.717 (0.366) | 1.55 | Up | **0.00101** |
| TG(14:0_34:2) | 40.958 (26.362) | 59.407 (23.423) | 1.45 | Up | **0.00103** |
| TG(14:0_34:3) | 7.572 (5.552) | 10.893 (4.188) | 1.44 | Up | **0.00103** |
| TG(14:0_32:2) | 5.173 (4.800) | 8.366 (4.485) | 1.62 | Up | **0.00117** |
| TG(20:3_32:1) | 3.621 (2.593) | 5.380 (2.737) | 1.49 | Up | **0.00122** |
| TG(20:4_32:1) | 6.917 (4.738) | 11.564 (7.221) | 1.67 | Up | **0.00122** |
| TG(18:1_30:1) | 26.473 (20.491) | 38.651 (18.896) | 1.46 | Up | **0.00129** |
| TG(18:3_32:1) | 9.331 (5.918) | 13.046 (4.780) | 1.4 | Up | **0.00134** |
| TG(20:4_32:2) | 2.141 (1.289) | 3.323 (1.596) | 1.55 | Up | **0.00134** |
| TG(17:1_32:1) | 2.299 (1.711) | 3.351 (1.484) | 1.46 | Up | **0.00147** |
| TG(20:5_34:1) | 2.279 (1.411) | 3.499 (1.746) | 1.54 | Up | **0.00159** |
| TG(16:1_32:1) | 29.399 (30.780) | 48.047 (32.824) | 1.63 | Up | **0.00162** |
| TG(16:0_32:1) | 129.415 (88.347) | 209.911 (126.891) | 1.62 | Up | **0.00168** |
| TG(14:0_34:1) | 61.127 (38.861) | 92.630 (46.656) | 1.52 | Up | **0.00171** |
| TG(16:0_38:7) | 1.592 (0.925) | 2.208 (0.848) | 1.39 | Up | **0.00209** |
| TG(16:0_30:2) | 6.361 (4.604) | 10.202 (5.639) | 1.6 | Up | **0.00213** |
| TG(18:1_30:0) | 70.963 (45.795) | 106.056 (52.799) | 1.49 | Up | **0.00213** |
| TG(18:1_28:1) | 3.355 (3.673) | 4.747 (2.829) | 1.41 | Up | **0.00225** |
| CE(15:0) | 8.834 (3.567) | 11.691 (3.874) | 1.32 | Up | **0.00241** |
| TG(14:0_35:2) | 1.460 (0.917) | 1.969 (0.711) | 1.35 | Up | **0.00242** |
| TG(16:1_32:0) | 32.434 (24.792) | 54.515 (36.840) | 1.68 | Up | **0.0025** |
| lysoPC a C28:1 | 0.461 (0.187) | 0.634 (0.262) | 1.37 | Up | **0.0026** |
| TG(18:2_30:1) | 13.478 (8.814) | 18.567 (7.493) | 1.38 | Up | **0.00269** |
| TG(14:0_38:4) | 1.515 (0.835) | 2.125 (0.855) | 1.4 | Up | **0.00284** |
| t4-OH-Pro | 11.609 (4.859) | 8.990 (3.057) | -1.29 | Down | **0.00299** |
| TG(14:0_34:0) | 8.581 (6.110) | 12.809 (6.824) | 1.49 | Up | **0.00305** |
| TG(16:0_36:6) | 1.773 (1.019) | 2.423 (1.034) | 1.37 | Up | **0.00315** |
| CE(17:0) | 5.967 (1.921) | 7.393 (2.061) | 1.24 | Up | **0.00348** |
| TG(22:6_32:1) | 3.793 (2.932) | 5.554 (2.858) | 1.46 | Up | **0.0035** |
| DG(14:1_18:1) | 0.127 (0.064) | 0.167 (0.052) | 1.32 | Up | **0.00356** |
| TG(16:1_34:3) | 13.452 (10.200) | 17.237 (6.357) | 1.28 | Up | **0.00363** |
| DG(16:0_16:1) | 1.186 (0.692) | 1.650 (0.798) | 1.39 | Up | **0.00369** |
| TG(18:2_28:0) | 7.401 (6.189) | 10.941 (5.858) | 1.48 | Up | **0.00396** |
| TG(16:0_28:2) | 1.978 (2.129) | 3.280 (2.265) | 1.66 | Up | **0.00424** |
| TG(18:3_32:0) | 13.472 (7.636) | 18.321 (7.213) | 1.36 | Up | **0.00438** |
| TG(18:0_30:1) | 2.159 (2.286) | 3.377 (2.607) | 1.56 | Up | **0.00477** |
| TG(18:0_32:1) | 11.661 (7.870) | 17.271 (10.547) | 1.48 | Up | **0.00477** |
| TG(18:1_26:0) | 3.812 (6.052) | 5.753 (4.960) | 1.51 | Up | **0.00537** |
| PC aa C36:5 | 15.164 (7.868) | 22.163 (11.790) | 1.46 | Up | **0.00593** |
| TG(16:0_35:1) | 11.922 (6.429) | 15.822 (6.671) | 1.33 | Up | **0.00593** |
| TG(14:0_36:1) | 9.395 (5.886) | 12.424 (5.707) | 1.32 | Up | **0.00655** |
| TG(20:4_32:0) | 11.409 (6.553) | 18.093 (10.826) | 1.59 | Up | **0.00665** |
| PC aa C34:3 | 27.815 (9.708) | 35.563 (12.691) | 1.28 | Up | **0.00688** |
| TG(18:0_32:2) | 4.354 (2.851) | 5.707 (2.464) | 1.31 | Up | **0.00734** |
| TG(18:1_33:0) | 10.503 (5.864) | 13.567 (5.404) | 1.29 | Up | **0.00746** |
| TG(18:0_30:0) | 4.801 (4.782) | 7.149 (4.793) | 1.49 | Up | **0.00759** |
| TG(16:1_38:5) | 2.070 (1.199) | 2.721 (1.073) | 1.31 | Up | **0.00822** |
| TG(16:1_34:0) | 16.979 (11.451) | 23.227 (11.728) | 1.37 | Up | **0.00835** |
| TG(16:0_34:3) | 102.693 (61.954) | 126.674 (37.457) | 1.23 | Up | **0.00876** |
| TG(18:1_32:3) | 5.912 (3.225) | 7.353 (2.718) | 1.24 | Up | **0.00919** |
| TG(22:6_32:0) | 6.112 (3.913) | 8.554 (4.198) | 1.4 | Up | **0.00919** |
| CE(14:1) | 1.167 (0.672) | 1.885 (1.380) | 1.62 | Up | **0.00963** |
| 3-Hydroxybutyric acid | 209.222 (434.447) | 40.426 (65.210) | -5.18 | Down | **0.00964** |
| TG(20:4_33:2) | 0.392 (0.195) | 0.535 (0.223) | 1.36 | Up | **0.00979** |
| SM C22:3 | 1.153 (0.592) | 0.765 (0.680) | -1.51 | Down | **0.00988** |
| CE(17:1) | 7.975 (2.543) | 9.769 (3.287) | 1.22 | Up | **0.00991** |
| TG(16:1_34:1) | 134.348 (97.097) | 174.470 (77.938) | 1.3 | Up | **0.0101** |
| TG(20:3_32:0) | 5.199 (2.871) | 7.701 (4.359) | 1.48 | Up | **0.01026** |
| TG(16:0_38:6) | 9.091 (3.884) | 11.618 (4.255) | 1.28 | Up | **0.01056** |
| CE(20:5) | 26.836 (14.260) | 41.948 (26.186) | 1.56 | Up | **0.01058** |
| TG(17:0_34:1) | 8.678 (4.448) | 11.029 (4.445) | 1.27 | Up | **0.01059** |
| PC ae C30:0 | 0.387 (0.140) | 0.482 (0.171) | 1.24 | Up | **0.01098** |
| lysoPC a C20:4 | 2.100 (1.042) | 2.854 (1.414) | 1.36 | Up | **0.01126** |
| TG(18:1_33:1) | 24.419 (14.677) | 29.774 (10.980) | 1.22 | Up | **0.0118** |
| TG(20:4_34:3) | 3.319 (1.521) | 4.280 (1.623) | 1.29 | Up | **0.01205** |
| TG(18:2_32:1) | 120.259 (70.424) | 142.922 (40.979) | 1.19 | Up | **0.01216** |
| PC aa C30:0 | 5.798 (2.110) | 7.931 (3.646) | 1.37 | Up | **0.01217** |
| TG(16:1_34:2) | 87.089 (59.937) | 106.693 (35.991) | 1.23 | Up | **0.01236** |
| PC aa C32:2 | 8.074 (3.335) | 10.318 (4.202) | 1.28 | Up | **0.01261** |
| PC ae C38:0 | 2.048 (0.714) | 2.483 (0.698) | 1.21 | Up | **0.01261** |
| CE(14:0) | 26.188 (11.362) | 36.441 (18.836) | 1.39 | Up | **0.01274** |
| PC aa C32:1 | 27.883 (16.170) | 41.478 (24.661) | 1.49 | Up | **0.01274** |
| TG(16:0_35:2) | 15.839 (8.474) | 19.727 (6.892) | 1.25 | Up | **0.01274** |
| PC ae C36:1 | 8.661 (2.237) | 10.088 (2.509) | 1.16 | Up | **0.01288** |
| TG(20:5_36:2) | 1.076 (0.572) | 1.427 (0.639) | 1.33 | Up | **0.01334** |
| TG(17:0_34:2) | 7.230 (3.517) | 8.976 (3.070) | 1.24 | Up | **0.01354** |
| Acetoacetate | 74.727 (123.572) | 20.085 (15.825) | -3.72 | Down | **0.01375** |
| TG(18:1_32:1) | 231.390 (156.060) | 288.259 (112.989) | 1.25 | Up | **0.01416** |
| Cer(d16:1/22:0) | 0.326 (0.132) | 0.395 (0.144) | 1.21 | Up | **0.01482** |
| TG(18:1_32:2) | 61.783 (35.369) | 73.059 (24.130) | 1.18 | Up | **0.01482** |
| Kynurenine | 1.392 (0.342) | 1.626 (0.385) | 1.17 | Up | **0.01502** |
| TG(22:4_32:0) | 2.046 (1.201) | 3.097 (1.918) | 1.51 | Up | **0.01505** |
| TG(17:1_34:3) | 1.066 (0.596) | 1.307 (0.418) | 1.23 | Up | **0.01574** |
| PC aa C40:6 | 27.284 (11.262) | 33.874 (11.186) | 1.24 | Up | **0.01677** |
| TrpBetaine | 0.408 (0.582) | 0.628 (0.599) | 1.54 | Up | **0.01772** |
| TG(18:3_38:6) | 0.452 (0.243) | 0.573 (0.228) | 1.27 | Up | **0.01772** |
| CE(18:3) | 81.719 (32.635) | 111.970 (60.155) | 1.37 | Up | **0.01851** |
| TG(22:5_32:1) | 2.768 (1.935) | 3.660 (1.953) | 1.32 | Up | **0.01851** |
| TG(18:1_30:2) | 5.977 (4.160) | 7.657 (3.995) | 1.28 | Up | **0.01906** |
| TG(16:0_34:2) | 461.208 (252.687) | 559.963 (194.730) | 1.21 | Up | **0.02049** |
| TG(18:2_33:0) | 6.701 (3.366) | 8.166 (3.063) | 1.22 | Up | **0.0217** |
| TG(14:0_36:2) | 45.243 (24.939) | 55.059 (20.086) | 1.22 | Up | **0.02233** |
| DG(18:2_20:0) | 0.085 (0.029) | 0.102 (0.033) | 1.2 | Up | **0.02263** |
| TG(20:3_34:3) | 1.623 (0.741) | 2.029 (0.746) | 1.25 | Up | **0.02264** |
| PC aa C28:1 | 3.975 (1.020) | 4.757 (1.540) | 1.2 | Up | **0.0229** |
| TG(18:3_33:2) | 0.633 (0.360) | 0.822 (0.396) | 1.3 | Up | **0.02331** |
| Citric acid | 71.024 (51.263) | 48.181 (22.713) | -1.47 | Down | **0.02364** |
| PC aa C34:4 | 2.522 (1.165) | 3.519 (1.834) | 1.4 | Up | **0.02397** |
| TG(17:1_34:1) | 10.393 (5.875) | 12.961 (5.066) | 1.25 | Up | **0.02397** |
| TG(16:1_38:4) | 2.499 (1.477) | 3.142 (1.243) | 1.26 | Up | **0.02466** |
| DG(16:1_18:2) | 1.527 (0.921) | 1.804 (0.585) | 1.18 | Up | **0.025** |
| EPA | 0.143 (0.230) | 0.101 (0.287) | -1.42 | Down | **0.02638** |
| PC aa C32:3 | 0.921 (0.241) | 1.093 (0.344) | 1.19 | Up | **0.02649** |
| FA(20:3) | 0.832 (1.111) | 0.339 (0.637) | -2.45 | Down | **0.02782** |
| TG(20:1_30:1) | 0.249 (0.179) | 0.316 (0.151) | 1.27 | Up | **0.0279** |
| TG(16:0_32:0) | 121.658 (68.357) | 164.993 (73.830) | 1.36 | Up | **0.02834** |
| TG(22:4_32:2) | 0.142 (0.082) | 0.181 (0.083) | 1.28 | Up | **0.02913** |
| PC ae C34:1 | 13.598 (3.492) | 15.617 (3.626) | 1.15 | Up | **0.03076** |
| DG(16:0_20:0) | 0.668 (0.291) | 0.828 (0.277) | 1.24 | Up | **0.03095** |
| SM (OH) C14:1 | 7.449 (1.937) | 8.584 (2.515) | 1.15 | Up | **0.03135** |
| TG(18:1_38:7) | 1.178 (0.496) | 1.439 (0.499) | 1.22 | Up | **0.03149** |
| Cer(d16:1/20:0) | 0.066 (0.023) | 0.078 (0.025) | 1.19 | Up | **0.03158** |
| Cer(d16:1/24:0) | 0.167 (0.077) | 0.210 (0.092) | 1.26 | Up | **0.0325** |
| Methanol | 350.178 (453.825) | 410.630 (219.385) | 1.17 | Up | **0.03431** |
| Acetone | 23.557 (35.109) | 10.622 (9.689) | -2.22 | Down | **0.03569** |
| Cer(d16:1/23:0) | 0.120 (0.047) | 0.151 (0.065) | 1.25 | Up | **0.03673** |
| TG(16:0_38:5) | 17.555 (7.620) | 21.852 (8.589) | 1.24 | Up | **0.03716** |
| 3-IAA | 1.259 (0.672) | 1.845 (1.440) | 1.47 | Up | **0.03816** |
| TG(20:4_36:5) | 1.033 (0.636) | 1.322 (0.678) | 1.28 | Up | **0.03817** |
| PC ae C34:0 | 1.761 (0.519) | 2.038 (0.607) | 1.16 | Up | **0.03969** |
| TG(18:3_38:5) | 0.713 (0.330) | 0.864 (0.317) | 1.21 | Up | **0.0413** |
| PC ae C30:2 | 0.109 (0.028) | 0.124 (0.033) | 1.13 | Up | **0.04201** |
| DG(16:0_18:1) | 8.814 (4.459) | 10.370 (3.610) | 1.18 | Up | **0.0435** |
| TG(17:1_34:2) | 7.252 (3.927) | 8.523 (2.842) | 1.18 | Up | **0.04351** |
| SM C18:1 | 14.383 (3.519) | 13.360 (3.721) | -1.08 | Down | **0.04402** |
| TG(20:4_34:2) | 26.848 (11.449) | 32.796 (13.409) | 1.22 | Up | **0.04508** |
| TG(20:5_36:3) | 1.018 (0.548) | 1.275 (0.573) | 1.25 | Up | **0.04582** |
| TG(20:3_34:2) | 10.838 (4.661) | 13.081 (5.091) | 1.21 | Up | **0.04639** |
| CE(16:0) | 299.980 (67.979) | 343.407 (81.634) | 1.14 | Up | **0.0464** |
| TG(20:3_34:0) | 1.879 (0.835) | 2.400 (1.031) | 1.28 | Up | **0.0464** |
| TG(17:2_34:3) | 0.778 (0.385) | 0.909 (0.323) | 1.17 | Up | **0.04761** |
| Acetic acid | 19.133 (32.018) | 10.448 (5.615) | -1.83 | Down | **0.04882** |
| Cortisone | 0.101 (0.065) | 0.075 (0.051) | -1.34 | Down | **0.0492** |

**Supplementary table S10. Metabolite Set Enrichment Analysis for altered pathways in PreE cases with maternal and neonatal adverse outcome**

| **Maternal adverse outcome** | | | | |
| --- | --- | --- | --- | --- |
| **Metabolite Set** | **Total** | **Hits** | **Impact** | **P value** |
| Alpha Linolenic Acid and Linoleic Acid Metabolism | 19 | 3 | 5.4238 | 0.0084 |
| Phospholipid Biosynthesis | 29 | 4 | 4.5495 | 0.00886 |
| Thyroid hormone synthesis | 13 | 2 | 5.7917 | 0.011433 |
| Arachidonic Acid Metabolism | 69 | 3 | 4.8299 | 0.013245 |
| Inositol Metabolism | 33 | 1 | 6.1295 | 0.031063 |
| Pyrimidine Metabolism | 59 | 1 | 6.1295 | 0.031063 |
| Starch and Sucrose Metabolism | 31 | 1 | 6.1295 | 0.031063 |
| Riboflavin Metabolism | 20 | 1 | 6.1295 | 0.031063 |
| Phosphatidylinositol Phosphate Metabolism | 17 | 1 | 6.1295 | 0.031063 |
| Catecholamine Biosynthesis | 20 | 1 | 5.454 | 0.04232 |
| **Neonatal adverse outcome** | | | | |
| **Metabolite Set** | **Total** | **Hits** | **Impact** | **P value** |
| Spinhoglipid metabolism | 40 | 4 | 6.7792 | 0.00122 |
| Phosphatidylethanolamine Biosynthesis | 12 | 2 | 7.1521 | 0.005338 |
| Methylhistidine Metabolism | 4 | 1 | 8.7918 | 0.0093 |
| Homocysteine Degradation | 9 | 2 | 6.9912 | 0.010357 |
| Steroidogenesis | 43 | 2 | 7.5215 | 0.011301 |
| Histidine Metabolism | 43 | 5 | 4.1041 | 0.012478 |
| Ammonia Recycling | 32 | 7 | 4.7361 | 0.013284 |
| Selenoamino Acid Metabolism | 28 | 2 | 6.789 | 0.014519 |
| Fatty Acid Elongation In Mitochondria | 35 | 1 | 7.6678 | 0.015453 |
| Steroid Biosynthesis | 48 | 3 | 4.9431 | 0.018674 |
| Glycerolipid Metabolism | 25 | 2 | 5.338 | 0.019142 |
| Beta-Alanine Metabolism | 34 | 4 | 3.6642 | 0.025946 |
| Glycine and Serine Metabolism | 59 | 12 | 3.516 | 0.031726 |
| Threonine and 2-Oxobutanoate Degradation | 20 | 1 | 5.9219 | 0.034152 |
| Fatty acid Metabolism | 43 | 2 | 4.3309 | 0.037281 |
| Methionine Metabolism | 43 | 8 | 3.2877 | 0.043707 |
| Aspartate Metabolism | 35 | 6 | 3.2539 | 0.048496 |
| Phospholipid Biosynthesis | 29 | 4 | 3.1953 | 0.048636 |

**Figure S1. PLS-DA plots : preeclampsia cases with vs without adverse outcomes)**

**
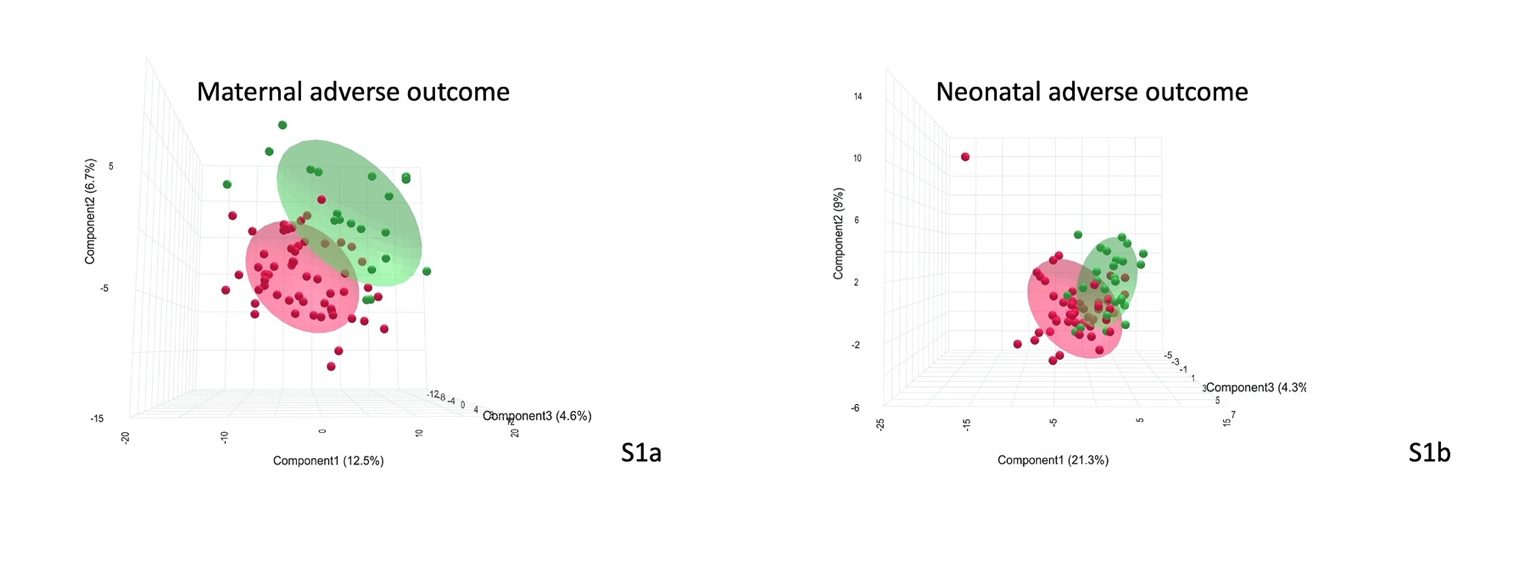
**
