# Supplementary material for: Metabolomic prediction of severe maternal and newborn complications in preeclampsia
Source: Metabolomics. 2024 May 18;20(3):56. doi: 10.1007/s11306-024-02123-0 (PMC11102370; doi:10.1007/s11306-024-02123-0)
Supplement: Supplementary file 2 — Supplementary file2 (DOCX 221 KB) [file 11306_2024_2123_MOESM2_ESM.docx]

**METABOLOMIC COMPARISION BETWEEN EARLY VS LATE ONSET PREECALMPSIA VS CONTROLS**

**Supplementary Table S7. Univariate analysis of metabolites and lipids, early vs controls**

| Name | Mean (SD) of Controls | Mean (SD) of Early Pre | Fold Change | Early Pre vs Controls | **p-value** |
| --- | --- | --- | --- | --- | --- |
| DG(18:1_20:3) | 0.129 (0.101) | 0.245 (0.086) | 1.9 | Up | **< 0.0001 (W)** |
| DG(16:0_20:0) | 0.612 (0.277) | 0.804 (0.258) | 1.32 | Up | **< 0.0001 (W)** |
| Cer(d16:1/20:0) | 0.054 (0.022) | 0.075 (0.023) | 1.39 | Up | **0.0001** |
| C5-DC (C6-OH) | 0.040 (0.036) | 0.070 (0.025) | 1.76 | Up | **0.0003 (W)** |
| TG(22:4_32:0) | 1.793 (1.501) | 2.908 (1.705) | 1.62 | Up | **0.0005 (W)** |
| Histamine | 0.010 (0.011) | 0.003 (0.004) | -3.36 | Down | **0.0007 (W)** |
| TG(20:4_34:2) | 23.263 (14.404) | 32.825 (12.317) | 1.41 | Up | **0.0008 (W)** |
| TG(18:2_31:0) | 3.931 (2.471) | 5.821 (2.258) | 1.48 | Up | **0.0009** |
| TG(20:4_32:2) | 1.982 (1.645) | 3.006 (1.548) | 1.52 | Up | **0.0009 (W)** |
| TG(20:4_30:0) | 2.587 (3.304) | 3.913 (2.860) | 1.51 | Up | **0.0009 (W)** |
| TG(20:4_32:0) | 10.771 (9.932) | 16.337 (9.504) | 1.52 | Up | **0.0010 (W)** |
| TG(20:4_34:3) | 3.005 (2.016) | 4.104 (1.544) | 1.37 | Up | **0.0010 (W)** |
| TG(22:4_34:2) | 3.417 (2.050) | 4.890 (2.017) | 1.43 | Up | **0.0011 (W)** |
| C5-OH (C3-DC-M) | 0.069 (0.046) | 0.101 (0.035) | 1.46 | Up | **0.0012 (W)** |
| EPA | 0.142 (0.202) | 0.066 (0.217) | -2.16 | Down | **0.0014 (W)** |
| TG(20:4_32:1) | 6.765 (6.944) | 10.477 (6.741) | 1.55 | Up | **0.0015 (W)** |
| TG(20:4_33:2) | 0.365 (0.227) | 0.511 (0.210) | 1.4 | Up | **0.0016 (W)** |
| pcPC a C16:0 | 43.059 (24.195) | 56.931 (21.160) | 1.32 | Up | **0.0017 (W)** |
| Cer(d18:1/26:1) | 0.024 (0.009) | 0.031 (0.010) | 1.31 | Up | **0.0017** |
| lysoPC a C16:1 | 0.876 (0.522) | 1.276 (0.728) | 1.46 | Up | **0.0018 (W)** |
| TG(20:4_34:1) | 30.713 (17.134) | 44.147 (18.859) | 1.44 | Up | **0.0018 (W)** |
| DG(18:2_20:4) | 0.383 (0.175) | 0.498 (0.153) | 1.3 | Up | **0.0020 (W)** |
| TG(18:1_31:0) | 5.633 (3.127) | 8.113 (3.647) | 1.44 | Up | **0.0021 (W)** |
| Cer(d18:0/18:0) | 0.021 (0.019) | 0.036 (0.023) | 1.76 | Up | **0.0023 (W)** |
| TG(20:4_36:2) | 12.920 (5.836) | 17.286 (6.309) | 1.34 | Up | **0.0025** |
| lysoPC a C20:4 | 2.191 (1.641) | 2.851 (1.267) | 1.3 | Up | **0.0025 (W)** |
| DG(18:1_20:4) | 0.438 (0.185) | 0.558 (0.138) | 1.27 | Up | **0.0026 (W)** |
| TG(22:4_32:2) | 0.125 (0.081) | 0.179 (0.084) | 1.43 | Up | **0.0029 (W)** |
| C16-OH | 0.020 (0.019) | 0.033 (0.016) | 1.66 | Up | **0.0029 (W)** |
| Acetic acid | 14.670 (6.580) | 10.764 (4.799) | -1.36 | Down | **0.0031 (W)** |
| TG(16:0_38:5) | 15.778 (8.602) | 21.508 (8.197) | 1.36 | Up | **0.0031 (W)** |
| lysoPC a C18:1 | 6.120 (3.686) | 7.820 (3.200) | 1.28 | Up | **0.0032 (W)** |
| TG(20:4_34:0) | 4.480 (2.515) | 6.319 (2.717) | 1.41 | Up | **0.0036 (W)** |
| TG(16:0_28:2) | 1.732 (1.974) | 2.607 (1.951) | 1.51 | Up | **0.0040 (W)** |
| CE(16:0) | 270.230 (88.907) | 333.722 (73.295) | 1.23 | Up | **0.0042 (W)** |
| TG(16:0_38:6) | 8.554 (5.705) | 11.123 (4.084) | 1.3 | Up | **0.0044 (W)** |
| TG(20:3_34:0) | 1.697 (0.838) | 2.374 (1.031) | 1.4 | Up | **0.0044 (W)** |
| TG(20:3_32:1) | 3.315 (2.338) | 5.155 (3.232) | 1.55 | Up | **0.0044 (W)** |
| TLCA | 0.002 (0.004) | 0.001 (0.003) | -2.2 | Down | **0.0044 (W)** |
| TG(20:3_34:2) | 9.539 (4.667) | 13.036 (5.050) | 1.37 | Up | **0.0044 (W)** |
| C6:1 | 0.024 (0.020) | 0.038 (0.016) | 1.61 | Up | **0.0046 (W)** |
| FA(20:3) | 0.807 (1.086) | 0.431 (0.954) | -1.87 | Down | **0.0048 (W)** |
| TG(14:0_38:5) | 1.080 (0.834) | 1.523 (0.779) | 1.41 | Up | **0.0049 (W)** |
| TG(18:1_28:1) | 2.721 (2.104) | 4.119 (2.547) | 1.51 | Up | **0.0051 (W)** |
| Cer(d16:1/22:0) | 0.282 (0.110) | 0.365 (0.141) | 1.29 | Up | **0.0053** |
| TG(16:0_28:1) | 4.932 (5.790) | 7.682 (7.019) | 1.56 | Up | **0.0053 (W)** |
| lysoPC a C20:3 | 0.856 (0.604) | 1.134 (0.573) | 1.32 | Up | **0.0054 (W)** |
| C18:1 | 0.041 (0.052) | 0.066 (0.043) | 1.63 | Up | **0.0059 (W)** |
| CE(20:4) | 310.895 (135.089) | 404.806 (154.000) | 1.3 | Up | **0.006** |
| C5:1-DC | 0.030 (0.027) | 0.046 (0.019) | 1.55 | Up | **0.0062 (W)** |
| TG(22:5_34:1 | 9.752 (6.452) | 12.266 (4.714) | 1.26 | Up | **0.0062 (W)** |
| DG(16:1_20:0) | 0.222 (0.473) | 0.390 (0.494) | 1.76 | Up | **0.0062 (W)** |
| TG(16:0_40:6) | 5.671 (3.159) | 7.555 (3.035) | 1.33 | Up | **0.0063 (W)** |
| DG(17:0_18:1) | 0.655 (0.244) | 0.820 (0.268) | 1.25 | Up | **0.0064** |
| TG(22:5_34:2) | 8.182 (4.491) | 11.087 (4.549) | 1.35 | Up | **0.0066** |
| TG(20:3_32:0) | 4.895 (3.490) | 7.131 (4.203) | 1.46 | Up | **0.0067 (W)** |
| C5-M-DC | 0.040 (0.029) | 0.056 (0.021) | 1.41 | Up | **0.0068 (W)** |
| TG(20:3_34:3) | 1.493 (0.777) | 1.982 (0.768) | 1.33 | Up | **0.0068 (W)** |
| TG(22:5_32:1) | 2.512 (1.979) | 3.657 (2.272) | 1.46 | Up | **0.0068 (W)** |
| TG(16:0_32:3) | 6.652 (5.401) | 9.297 (4.565) | 1.4 | Up | **0.0069 (W)** |
| TG(22:5_32:0) | 3.999 (2.917) | 5.706 (3.168) | 1.43 | Up | **0.0069 (W)** |
| TG(22:5_34:3) | 1.291 (0.741) | 1.762 (0.701) | 1.36 | Up | **0.0070 (W)** |
| TG(18:1_26:0) | 2.932 (3.662) | 4.479 (4.351) | 1.53 | Up | **0.0074 (W)** |
| TG(16:1_38:5) | 1.904 (1.140) | 2.677 (1.328) | 1.41 | Up | **0.0077 (W)** |
| DiCA(14:0) | 0.056 (0.059) | 0.028 (0.026) | -2 | Down | **0.0078 (W)** |
| CE(20:3) | 37.911 (15.089) | 50.425 (18.898) | 1.33 | Up | **0.0079 (W)** |
| CE(22:5) | 4.235 (2.081) | 5.549 (2.138) | 1.31 | Up | **0.0083** |
| TG(20:3_32:2) | 0.975 (0.594) | 1.407 (0.750) | 1.44 | Up | **0.0085 (W)** |
| TG(20:4_36:3) | 10.311 (5.216) | 13.322 (4.407) | 1.29 | Up | **0.0085** |
| C4:1 | 0.034 (0.015) | 0.044 (0.015) | 1.27 | Up | **0.0086** |
| TG(16:1_30:1) | 3.011 (3.265) | 5.110 (4.742) | 1.7 | Up | **0.0086 (W)** |
| TG(18:0_30:0) | 4.114 (3.657) | 6.048 (4.312) | 1.47 | Up | **0.0092 (W)** |
| C16:1-OH | 0.012 (0.011) | 0.020 (0.012) | 1.58 | Up | **0.0092 (W)** |
| TG(18:0_32:0) | 13.389 (8.903) | 17.948 (7.715) | 1.34 | Up | **0.0093 (W)** |
| TG(17:0_34:3) | 1.302 (0.686) | 1.736 (0.734) | 1.33 | Up | **0.0094** |
| TG(20:4_35:3) | 0.140 (0.075) | 0.182 (0.069) | 1.3 | Up | **0.0096 (W)** |
| TG(16:0_34:4) | 7.283 (5.229) | 9.509 (4.035) | 1.31 | Up | **0.0099 (W)** |
| ProBetaine | 3.834 (5.678) | 8.110 (11.546) | 2.12 | Up | **0.0102 (W)** |
| lysoPC a C17:0 | 0.583 (0.319) | 0.732 (0.255) | 1.26 | Up | **0.0102 (W)** |
| TG(16:0_30:2) | 6.116 (5.780) | 8.660 (5.428) | 1.42 | Up | **0.0102 (W)** |
| TG(18:2_32:1) | 103.550 (53.540) | 137.044 (57.156) | 1.32 | Up | **0.0102** |
| TG(16:1_28:0) | 2.046 (2.596) | 3.142 (2.759) | 1.54 | Up | **0.0103 (W)** |
| TG(18:0_32:2) | 3.756 (2.632) | 5.200 (2.586) | 1.38 | Up | **0.0103 (W)** |
| TG(18:0_38:7) | 0.310 (0.187) | 0.402 (0.149) | 1.3 | Up | **0.0103 (W)** |
| TG(18:3_30:0) | 3.249 (3.132) | 4.342 (2.483) | 1.34 | Up | **0.0106 (W)** |
| lysoPC a C28:1 | 0.416 (0.163) | 0.567 (0.243) | 1.36 | Up | **0.0110 (W)** |
| TG(14:0_34:3) | 6.800 (5.018) | 9.690 (4.969) | 1.42 | Up | **0.0110 (W)** |
| DG(16:0_16:1) | 1.110 (0.735) | 1.572 (0.878) | 1.42 | Up | **0.0111 (W)** |
| C7-DC | 0.034 (0.028) | 0.054 (0.019) | 1.56 | Up | **0.0111 (W)** |
| DiCA(12:0) | 0.151 (0.154) | 0.080 (0.055) | -1.89 | Down | **0.0113 (W)** |
| TG(16:0_32:2) | 51.458 (39.895) | 74.089 (41.257) | 1.44 | Up | **0.0115 (W)** |
| TG(14:0_34:2) | 37.518 (27.378) | 52.856 (25.979) | 1.41 | Up | **0.0118 (W)** |
| PC aa C40:4 | 3.929 (1.461) | 4.885 (1.768) | 1.24 | Up | **0.0118** |
| TG(18:3_32:0) | 12.814 (9.024) | 16.149 (7.031) | 1.26 | Up | **0.0120 (W)** |
| C10:2 | 0.106 (0.085) | 0.165 (0.071) | 1.55 | Up | **0.0120 (W)** |
| C14 | 0.086 (0.074) | 0.131 (0.064) | 1.52 | Up | **0.0124 (W)** |
| TG(18:2_30:1) | 11.922 (8.281) | 16.581 (8.588) | 1.39 | Up | **0.0127 (W)** |
| TG(18:2_28:0) | 6.660 (6.578) | 9.071 (5.623) | 1.36 | Up | **0.0129 (W)** |
| TG(16:1_32:2) | 6.899 (5.202) | 10.835 (8.329) | 1.57 | Up | **0.0131 (W)** |
| TG(18:2_30:0) | 35.458 (25.942) | 47.669 (21.490) | 1.34 | Up | **0.0131 (W)** |
| TG(18:3_38:5) | 0.687 (0.420) | 0.817 (0.283) | 1.19 | Up | **0.0131 (W)** |
| TG(22:0_32:4) | 0.146 (0.090) | 0.186 (0.058) | 1.28 | Up | **0.0132 (W)** |
| TG(16:0_38:7) | 1.629 (1.317) | 1.968 (0.877) | 1.21 | Up | **0.0135 (W)** |
| CE(14:1) | 1.111 (0.764) | 1.585 (1.145) | 1.43 | Up | **0.0135 (W)** |
| TG(17:1_38:7) | 0.034 (0.022) | 0.044 (0.018) | 1.32 | Up | **0.0138 (W)** |
| TG(16:0_33:2) | 6.768 (4.275) | 9.014 (3.893) | 1.33 | Up | **0.0141 (W)** |
| TG(20:4_36:4) | 4.513 (2.881) | 5.740 (2.265) | 1.27 | Up | **0.0141 (W)** |
| TG(17:2_38:7) | 0.168 (0.138) | 0.223 (0.096) | 1.33 | Up | **0.0147 (W)** |
| TG(18:2_38:5) | 5.769 (2.916) | 7.288 (2.657) | 1.26 | Up | **0.0149 (W)** |
| TG(16:0_34:2) | 405.767 (228.496) | 536.028 (226.482) | 1.32 | Up | **0.0149** |
| TG(18:0_38:6) | 1.620 (0.930) | 2.137 (1.053) | 1.32 | Up | **0.0158 (W)** |
| TG(16:0_34:3) | 90.712 (53.617) | 120.086 (50.158) | 1.32 | Up | **0.0163** |
| TG(14:0_32:2) | 4.813 (4.555) | 7.138 (4.815) | 1.48 | Up | **0.0165 (W)** |
| TG(17:1_34:2) | 6.388 (3.152) | 8.271 (3.560) | 1.29 | Up | **0.0168** |
| TG(16:1_36:1) | 11.502 (5.683) | 15.503 (8.149) | 1.35 | Up | **0.0169** |
| TG(16:1_34:2) | 75.213 (43.464) | 104.500 (54.169) | 1.39 | Up | **0.0170 (W)** |
| TG(18:2_38:4) | 5.547 (2.898) | 7.055 (2.895) | 1.27 | Up | **0.0170 (W)** |
| TG(20:3_34:1) | 12.903 (6.277) | 17.590 (7.987) | 1.36 | Up | **0.0170 (W)** |
| CE(17:0) | 5.682 (2.249) | 6.873 (1.994) | 1.21 | Up | **0.0175** |
| DG-O(16:0_18:1) | 0.094 (0.061) | 0.136 (0.053) | 1.44 | Up | **0.0179 (W)** |
| TG(16:1_32:1) | 28.739 (29.147) | 45.072 (40.258) | 1.57 | Up | **0.0182 (W)** |
| TG(17:0_34:2) | 6.465 (3.483) | 8.383 (3.431) | 1.3 | Up | **0.0182** |
| TG(16:1_33:1) | 3.079 (2.062) | 4.595 (2.979) | 1.49 | Up | **0.0185 (W)** |
| TG(16:0_32:1) | 127.844 (110.908) | 183.089 (123.621) | 1.43 | Up | **0.0185 (W)** |
| TG(18:3_32:1) | 8.919 (6.251) | 11.530 (5.205) | 1.29 | Up | **0.0185 (W)** |
| TG(18:1_30:0) | 67.103 (49.869) | 91.994 (52.686) | 1.37 | Up | **0.0187 (W)** |
| DG(14:1_20:2) | 0.037 (0.038) | 0.056 (0.035) | 1.54 | Up | **0.0188 (W)** |
| TG(14:0_34:1) | 58.513 (45.516) | 80.400 (45.961) | 1.37 | Up | **0.0192 (W)** |
| TG(16:1_32:0) | 33.602 (34.208) | 47.931 (36.036) | 1.43 | Up | **0.0192 (W)** |
| TG(16:1_38:4) | 2.229 (1.118) | 3.156 (1.633) | 1.42 | Up | **0.0192 (W)** |
| TG(17:2_34:3) | 0.684 (0.348) | 0.880 (0.368) | 1.29 | Up | **0.0193** |
| TG(14:0_38:4) | 1.405 (0.885) | 1.949 (0.916) | 1.39 | Up | **0.0195 (W)** |
| Cer(d16:1/18:0) | 0.081 (0.033) | 0.096 (0.024) | 1.19 | Up | **0.0196** |
| TG(18:0_34:3) | 6.538 (3.845) | 8.357 (3.406) | 1.28 | Up | **0.0198 (W)** |
| TG(17:0_32:1) | 2.261 (1.693) | 3.198 (1.926) | 1.41 | Up | **0.0201 (W)** |
| TG(20:2_32:1) | 3.253 (1.737) | 4.491 (2.259) | 1.38 | Up | **0.0201 (W)** |
| TG(17:1_34:3) | 0.966 (0.470) | 1.251 (0.577) | 1.3 | Up | **0.0201** |
| TG(16:0_38:4) | 18.127 (9.159) | 23.531 (9.231) | 1.3 | Up | **0.0206 (W)** |
| TG(20:5_34:0) | 0.467 (0.338) | 0.620 (0.321) | 1.33 | Up | **0.0212 (W)** |
| TG(16:0_35:1) | 11.010 (6.539) | 14.617 (7.135) | 1.33 | Up | **0.0218 (W)** |
| TG(18:1_30:1) | 23.919 (16.771) | 34.941 (22.270) | 1.46 | Up | **0.0218 (W)** |
| TG(20:2_34:4) | 0.119 (0.079) | 0.143 (0.055) | 1.2 | Up | **0.0221 (W)** |
| TG(16:0_34:0) | 66.587 (40.363) | 88.594 (41.805) | 1.33 | Up | **0.0223** |
| TG(18:0_30:1) | 1.904 (1.613) | 2.804 (2.358) | 1.47 | Up | **0.0224 (W)** |
| TG(18:1_38:5) | 9.198 (3.949) | 11.968 (4.675) | 1.3 | Up | **0.0224 (W)** |
| TG(22:5_34:1) | 9.739 (5.232) | 13.123 (6.007) | 1.35 | Up | **0.0224 (W)** |
| TG(20:4_36:5) | 1.014 (0.841) | 1.273 (0.677) | 1.25 | Up | **0.0227 (W)** |
| TG(16:0_32:0) | 112.463 (84.301) | 152.497 (77.512) | 1.36 | Up | **0.0230 (W)** |
| TG(16:0_33:1) | 14.155 (9.539) | 18.772 (9.814) | 1.33 | Up | **0.0230 (W)** |
| 3-Hydroxybutyric acid | 117.022 (159.912) | 85.039 (174.525) | -1.38 | Down | **0.0237 (W)** |
| TG(18:0_36:1) | 7.190 (4.604) | 9.805 (5.356) | 1.36 | Up | **0.0236 (W)** |
| TG(18:1_38:7) | 1.111 (0.575) | 1.393 (0.478) | 1.25 | Up | **0.0237** |
| C3-OH | 0.034 (0.022) | 0.046 (0.018) | 1.34 | Up | **0.0238 (W)** |
| Malonate | 73.623 (324.584) | 4.450 (4.464) | -16.54 | Down | **0.0239 (W)** |
| CE(17:1) | 7.567 (3.130) | 9.193 (3.005) | 1.21 | Up | **0.024** |
| TG(18:3_33:2) | 0.612 (0.464) | 0.733 (0.317) | 1.2 | Up | **0.0243 (W)** |
| TG(20:5_34:2) | 1.862 (1.509) | 2.356 (1.180) | 1.27 | Up | **0.0243 (W)** |
| TG(20:3_36:4) | 1.420 (0.756) | 1.744 (0.625) | 1.23 | Up | **0.0246 (W)** |
| TG(17:2_36:2) | 0.564 (0.260) | 0.706 (0.278) | 1.25 | Up | **0.0247** |
| TG(14:0_34:0) | 7.915 (6.186) | 10.999 (6.575) | 1.39 | Up | **0.0250 (W)** |
| TG(16:1_34:0) | 15.268 (10.354) | 21.963 (13.384) | 1.44 | Up | **0.0250 (W)** |
| TG(18:0_32:1) | 10.665 (7.614) | 15.297 (10.098) | 1.43 | Up | **0.0260 (W)** |
| TG(18:2_33:0) | 6.011 (3.315) | 7.591 (3.269) | 1.26 | Up | **0.0260 (W)** |
| TG(18:3_38:6) | 0.455 (0.321) | 0.540 (0.231) | 1.19 | Up | **0.0263 (W)** |
| TG(20:5_34:1) | 2.448 (1.949) | 3.023 (1.493) | 1.24 | Up | **0.0263 (W)** |
| TG(18:0_34:2) | 35.454 (21.202) | 46.036 (19.329) | 1.3 | Up | **0.0264** |
| TG(22:6_32:1) | 4.252 (4.741) | 4.938 (3.098) | 1.16 | Up | **0.0271 (W)** |
| TG(16:1_34:3) | 11.749 (7.133) | 16.703 (9.815) | 1.42 | Up | **0.0274 (W)** |
| lysoPC a C24:0 | 0.118 (0.049) | 0.143 (0.046) | 1.21 | Up | **0.0278 (W)** |
| CE(18:3) | 76.145 (33.321) | 99.811 (49.326) | 1.31 | Up | **0.0278 (W)** |
| TG(14:0_35:2) | 1.348 (0.873) | 1.768 (0.841) | 1.31 | Up | **0.0281 (W)** |
| SM (OH) C14:1 | 6.745 (2.363) | 7.943 (2.307) | 1.18 | Up | **0.0286** |
| Creatine | 23.855 (12.739) | 31.553 (16.376) | 1.32 | Up | **0.0289 (W)** |
| DG(22:1_22:2) | 0.021 (0.021) | 0.032 (0.022) | 1.58 | Up | **0.0289 (W)** |
| DG(16:1_18:2) | 1.349 (0.734) | 1.729 (0.751) | 1.28 | Up | **0.0289** |
| CE(15:1) | 0.509 (0.245) | 0.625 (0.207) | 1.23 | Up | **0.0289** |
| CE(20:1) | 1.179 (0.406) | 1.601 (1.347) | 1.36 | Up | **0.0297 (W)** |
| Choline.1 | 7.983 (2.941) | 9.516 (3.092) | 1.19 | Up | **0.0298** |
| C6 (C4:1-DC) | 0.103 (0.086) | 0.158 (0.068) | 1.54 | Up | **0.0307 (W)** |
| PC aa C28:1 | 3.630 (1.313) | 4.315 (1.401) | 1.19 | Up | **0.031** |
| Hex3Cer(d18:1/26:1) | 0.226 (0.094) | 0.274 (0.096) | 1.21 | Up | **0.0312** |
| TG(18:1_30:2) | 5.162 (3.581) | 6.884 (3.996) | 1.33 | Up | **0.0313 (W)** |
| Dimethyl sulfone | 17.453 (60.262) | 3.839 (3.998) | -4.55 | Down | **0.0316 (W)** |
| CE(18:1) | 491.980 (203.674) | 585.917 (166.949) | 1.19 | Up | **0.0321** |
| CE(18:0) | 17.749 (6.553) | 20.719 (5.156) | 1.17 | Up | **0.0325** |
| lysoPC a C18:2 | 10.092 (7.108) | 12.222 (5.575) | 1.21 | Up | **0.0325 (W)** |
| SM C24:1 | 42.047 (14.450) | 49.094 (11.097) | 1.17 | Up | **0.0325 (W)** |
| TG(14:0_36:1) | 8.392 (5.199) | 11.180 (5.788) | 1.33 | Up | **0.0325 (W)** |
| TG(14:0_36:2) | 40.390 (22.089) | 52.117 (24.882) | 1.29 | Up | **0.0327** |
| TG(16:0_35:2) | 14.653 (7.813) | 18.679 (8.317) | 1.27 | Up | **0.0328** |
| TG(14:0_35:1) | 1.385 (1.072) | 1.842 (1.075) | 1.33 | Up | **0.0329 (W)** |
| TG(17:1_34:1) | 9.584 (5.248) | 12.374 (5.951) | 1.29 | Up | **0.0331** |
| C8 | 0.200 (0.172) | 0.300 (0.137) | 1.5 | Up | **0.0337 (W)** |
| TG(20:2_32:0) | 4.725 (2.905) | 6.168 (2.798) | 1.31 | Up | **0.0338 (W)** |
| SM C22:3 | 0.666 (0.478) | 0.960 (0.679) | 1.44 | Up | **0.0345 (W)** |
| TG(18:1_32:3) | 5.395 (2.975) | 6.854 (2.919) | 1.27 | Up | **0.0345** |
| TG(18:2_33:1) | 12.485 (6.228) | 15.758 (7.045) | 1.26 | Up | **0.0349** |
| Propylene glycol | 0.248 (0.260) | 0.432 (0.410) | 1.74 | Up | **0.0354 (W)** |
| SM C20:2 | 0.643 (0.265) | 0.792 (0.341) | 1.23 | Up | **0.0355** |
| SM (OH) C22:2 | 8.876 (2.995) | 10.461 (2.852) | 1.18 | Up | **0.0356 (W)** |
| DG(18:1_20:1) | 0.215 (0.083) | 0.257 (0.076) | 1.19 | Up | **0.0365 (W)** |
| TG(22:6_32:0) | 7.042 (7.677) | 7.552 (4.341) | 1.07 | Up | **0.0365 (W)** |
| Cer(d18:1/14:0) | 0.069 (0.030) | 0.079 (0.021) | 1.15 | Up | **0.0370 (W)** |
| DG(16:0_18:1) | 8.020 (3.851) | 9.946 (4.048) | 1.24 | Up | **0.037** |
| TG(18:1_33:0) | 9.613 (5.472) | 12.525 (6.078) | 1.3 | Up | **0.0370 (W)** |
| TG(17:1_32:1) | 2.180 (1.565) | 3.065 (1.914) | 1.41 | Up | **0.0370 (W)** |
| TG(18:2_36:0) | 5.907 (3.901) | 7.125 (3.048) | 1.21 | Up | **0.0370 (W)** |
| CE(15:0) | 8.426 (4.036) | 10.284 (3.568) | 1.22 | Up | **0.0377** |
| TG(16:0_38:1) | 2.617 (1.344) | 3.445 (1.551) | 1.32 | Up | **0.0379 (W)** |
| DG(16:0_18:2) | 5.938 (2.895) | 7.318 (2.787) | 1.23 | Up | **0.038** |
| TG(18:2_32:0) | 169.937 (97.348) | 217.003 (96.656) | 1.28 | Up | **0.0381** |
| DG(14:1_18:1) | 0.125 (0.079) | 0.151 (0.067) | 1.21 | Up | **0.0383 (W)** |
| DG(18:2_20:0) | 0.080 (0.034) | 0.096 (0.034) | 1.21 | Up | **0.0392** |
| C14:2 | 0.042 (0.034) | 0.060 (0.031) | 1.42 | Up | **0.0404 (W)** |
| TG(16:1_34:1) | 120.669 (77.725) | 168.006 (99.934) | 1.39 | Up | **0.0404 (W)** |
| TG(16:0_34:1) | 508.144 (306.170) | 658.778 (324.073) | 1.3 | Up | **0.0407** |
| Isobutyric acid | 4.910 (2.537) | 5.978 (2.481) | 1.22 | Up | **0.0414 (W)** |
| TG(16:0_35:3) | 6.314 (3.221) | 7.818 (3.107) | 1.24 | Up | **0.0423** |
| TG(20:1_32:1) | 2.596 (1.490) | 3.575 (2.009) | 1.38 | Up | **0.0425 (W)** |
| Acetone | 17.363 (21.698) | 14.758 (18.086) | -1.18 | Down | **0.0430 (W)** |
| TG(20:3_36:3) | 3.266 (1.485) | 4.061 (1.461) | 1.24 | Up | **0.0430 (W)** |
| C3:1 | 0.021 (0.012) | 0.027 (0.009) | 1.27 | Up | **0.0430 (W)** |
| CE(16:1) | 97.307 (60.396) | 129.414 (70.618) | 1.33 | Up | **0.0435 (W)** |
| TG(20:1_32:2) | 1.184 (0.587) | 1.549 (0.684) | 1.31 | Up | **0.0436 (W)** |
| TG(16:0_36:6) | 1.919 (1.661) | 2.087 (0.846) | 1.09 | Up | **0.0436 (W)** |
| TG(18:1_32:1) | 205.231 (117.947) | 279.019 (154.101) | 1.36 | Up | **0.0447 (W)** |
| Pro | 133.436 (47.550) | 155.256 (45.322) | 1.16 | Up | **0.0447** |
| Cortisone | 0.099 (0.059) | 0.075 (0.048) | -1.33 | Down | **0.0449 (W)** |
| TG(18:1_32:2) | 53.219 (26.077) | 70.186 (33.543) | 1.32 | Up | **0.0463 (W)** |
| TG(17:0_34:1) | 8.067 (4.625) | 10.189 (4.798) | 1.26 | Up | **0.0469 (W)** |
| TG(16:0_37:3) | 2.227 (1.119) | 2.723 (1.121) | 1.22 | Up | **0.0475 (W)** |
| PC ae C30:2 | 0.102 (0.036) | 0.118 (0.033) | 1.16 | Up | **0.0486 (W)** |
| TG(22:2_32:4) | 0.163 (0.151) | 0.177 (0.082) | 1.09 | Up | **0.0493 (W)** |
| TG(18:1_36:0) | 8.198 (4.266) | 10.519 (4.950) | 1.28 | Up | **0.0499 (W)** |
| PC aa C34:3 | 26.636 (10.201) | 31.419 (10.712) | 1.18 | Up | **0.0499** |
| L-Phenylalanine | 32.790 (13.510) | 39.056 (13.895) | 1.19 | Up | 0.0501 |
| Cer(d18:0/18:0(OH)) | 0.306 (0.260) | 0.434 (0.269) | 1.42 | Up | 0.0502 (W) |
| TG(18:3_34:0) | 6.716 (3.893) | 7.999 (2.961) | 1.19 | Up | 0.0511 (W) |
| PC aa C30:0 | 5.463 (2.677) | 6.874 (3.197) | 1.26 | Up | 0.0517 (W) |
| CE(14:0) | 25.084 (13.228) | 31.986 (16.140) | 1.28 | Up | 0.0523 (W) |
| PC aa C38:3 | 54.514 (19.872) | 64.083 (16.969) | 1.18 | Up | 0.0523 (W) |
| TG(18:1_32:0) | 310.768 (188.842) | 398.714 (200.129) | 1.28 | Up | 0.0525 |
| DG(16:1_18:0) | 0.239 (0.121) | 0.292 (0.113) | 1.22 | Up | 0.053 |
| TG(20:2_34:1) | 10.123 (4.973) | 12.410 (5.175) | 1.23 | Up | 0.0534 |
| TG(20:1_34:0) | 1.434 (0.751) | 1.773 (0.752) | 1.24 | Up | 0.0534 |
| GDCA | 0.530 (0.663) | 0.734 (0.678) | 1.38 | Up | 0.0542 (W) |
| PC aa C38:5 | 51.166 (18.299) | 62.761 (20.589) | 1.23 | Up | 0.0543 (W) |
| Cer(d18:1/20:0(OH)) | 1.492 (0.627) | 1.794 (0.725) | 1.2 | Up | 0.0557 |
| TG(20:2_36:5) | 0.213 (0.154) | 0.241 (0.100) | 1.13 | Up | 0.0563 (W) |
| HexCer(d16:1/24:0) | 0.093 (0.043) | 0.113 (0.050) | 1.22 | Up | 0.0565 |
| C16 | 0.089 (0.035) | 0.099 (0.024) | 1.11 | Up | 0.0576 (W) |
| HCys | 4.672 (2.967) | 5.900 (3.044) | 1.26 | Up | 0.0576 (W) |
| HexCer(d18:1/26:1) | 0.091 (0.033) | 0.105 (0.026) | 1.15 | Up | 0.0578 |
| TG(20:1_34:3) | 1.291 (0.680) | 1.592 (0.685) | 1.23 | Up | 0.0581 |
| PC aa C38:4 | 110.013 (39.328) | 132.944 (45.914) | 1.21 | Up | 0.0583 (W) |
| C14:2-OH | 0.021 (0.013) | 0.027 (0.010) | 1.25 | Up | 0.0588 (W) |
| TG(16:0_40:7) | 6.079 (4.381) | 7.084 (3.177) | 1.17 | Up | 0.0590 (W) |
| TG(20:2_34:2) | 7.608 (4.165) | 9.171 (3.655) | 1.21 | Up | 0.0590 (W) |
| SM C26:1 | 0.217 (0.078) | 0.259 (0.079) | 1.19 | Up | 0.0597 (W) |
| HexCer(d18:2/16:0) | 0.073 (0.031) | 0.086 (0.025) | 1.17 | Up | 0.0599 |
| GABA | 0.192 (0.074) | 0.223 (0.069) | 1.16 | Up | 0.0618 |
| TG(18:1_33:3) | 0.897 (0.439) | 1.103 (0.436) | 1.23 | Up | 0.0633 (W) |
| FA(20:1) | 1.706 (2.057) | 1.183 (2.054) | -1.44 | Down | 0.0638 (W) |
| TG(20:3_36:5) | 0.323 (0.190) | 0.367 (0.134) | 1.14 | Up | 0.0648 (W) |
| TG(18:2_34:0) | 78.459 (40.824) | 95.900 (40.277) | 1.22 | Up | 0.0652 |
| TG(16:0_38:3) | 17.157 (8.601) | 20.837 (8.543) | 1.21 | Up | 0.0657 |
| DG(16:0_20:3) | 0.068 (0.038) | 0.086 (0.033) | 1.27 | Up | 0.0667 (W) |
| PC aa C42:5 | 0.457 (0.175) | 0.528 (0.153) | 1.15 | Up | 0.0669 |
| TG(20:1_30:1) | 0.230 (0.138) | 0.308 (0.210) | 1.34 | Up | 0.0677 (W) |
| Methionine | 14.377 (6.147) | 17.306 (7.438) | 1.2 | Up | 0.0678 (W) |
| CA | 0.069 (0.140) | 0.103 (0.128) | 1.5 | Up | 0.0694 (W) |
| Tyr | 41.170 (16.709) | 49.550 (17.466) | 1.2 | Up | 0.0694 (W) |
| TG(20:5_36:2) | 1.106 (0.696) | 1.282 (0.511) | 1.16 | Up | 0.0703 (W) |
| PC aa C32:3 | 0.859 (0.304) | 0.999 (0.260) | 1.16 | Up | 0.0710 (W) |
| TG(18:1_38:6) | 6.094 (3.639) | 7.006 (2.596) | 1.15 | Up | 0.0719 (W) |
| TG(18:1_33:2) | 9.623 (4.819) | 11.752 (5.362) | 1.22 | Up | 0.0722 |
| TG(14:0_36:4) | 15.310 (9.605) | 18.442 (8.134) | 1.2 | Up | 0.0744 (W) |
| Cer(d18:1/20:0) | 0.290 (0.122) | 0.343 (0.119) | 1.18 | Up | 0.0744 (W) |
| TG(14:0_36:3) | 41.257 (21.584) | 51.431 (22.127) | 1.25 | Up | 0.0744 (W) |
| TG(18:1_33:1) | 22.715 (12.251) | 28.077 (13.622) | 1.24 | Up | 0.0748 |
| Ethanol | 191.440 (132.882) | 135.994 (85.964) | -1.41 | Down | 0.0752 (W) |
| C12 | 0.092 (0.070) | 0.125 (0.067) | 1.35 | Up | 0.0755 (W) |
| DG(16:0_20:4) | 0.070 (0.038) | 0.089 (0.028) | 1.28 | Up | 0.0756 (W) |
| TG(22:6_34:3) | 1.931 (1.557) | 2.382 (1.467) | 1.23 | Up | 0.0778 (W) |
| PC ae C42:1 | 0.365 (0.123) | 0.413 (0.094) | 1.13 | Up | 0.0787 (W) |
| TG(18:2_38:6) | 3.992 (2.356) | 4.630 (1.957) | 1.16 | Up | 0.0787 (W) |
| Hex3Cer(d18:1/16:0) | 1.192 (0.412) | 1.345 (0.324) | 1.13 | Up | 0.079 |
| C9 | 0.031 (0.014) | 0.038 (0.015) | 1.23 | Up | 0.0803 (W) |
| SM C24:0 | 17.980 (6.088) | 20.867 (5.036) | 1.16 | Up | 0.0805 (W) |
| C5:1 | 0.046 (0.030) | 0.059 (0.019) | 1.28 | Up | 0.0808 (W) |
| TG(18:2_32:2) | 28.392 (17.392) | 34.936 (15.547) | 1.23 | Up | 0.0823 (W) |
| Glycerol | 137.865 (46.221) | 154.689 (46.933) | 1.12 | Up | 0.0823 (W) |
| TG(16:1_38:3) | 1.751 (0.825) | 2.368 (1.337) | 1.35 | Up | 0.0832 (W) |
| TG(16:0_40:8) | 2.983 (2.207) | 3.566 (1.998) | 1.2 | Up | 0.0842 (W) |
| p-Cresol-SO4 | 14.578 (9.770) | 20.438 (13.871) | 1.4 | Up | 0.0851 (W) |
| TG(20:0_34:1) | 1.997 (0.803) | 2.301 (0.702) | 1.15 | Up | 0.0852 |
| TG(18:0_36:5) | 2.654 (2.941) | 2.631 (1.190) | -1.01 | Down | 0.0860 (W) |
| Cer(d18:0/20:0) | 0.063 (0.035) | 0.079 (0.029) | 1.24 | Up | 0.0877 (W) |
| PC aa C40:5 | 9.296 (3.650) | 11.393 (4.162) | 1.23 | Up | 0.0880 (W) |
| GCA | 0.375 (0.467) | 0.534 (0.691) | 1.42 | Up | 0.0880 (W) |
| TG(20:5_36:3) | 1.009 (0.643) | 1.184 (0.529) | 1.17 | Up | 0.0880 (W) |
| TG(20:1_34:1) | 10.102 (5.396) | 12.274 (5.591) | 1.22 | Up | 0.0892 |
| C16:2 | 0.022 (0.018) | 0.029 (0.015) | 1.27 | Up | 0.0893 (W) |
| HexCer(d18:2/20:0) | 0.076 (0.033) | 0.089 (0.033) | 1.17 | Up | 0.0909 |
| PC aa C32:2 | 7.591 (3.458) | 9.037 (3.905) | 1.19 | Up | 0.0912 |
| TDCA | 0.225 (0.357) | 0.304 (0.438) | 1.36 | Up | 0.0918 (W) |
| C18:2 | 0.041 (0.020) | 0.047 (0.019) | 1.14 | Up | 0.0928 (W) |
| TG(18:1_34:2) | 659.230 (316.974) | 788.194 (343.335) | 1.2 | Up | 0.0928 |
| Acetoacetate | 47.650 (48.909) | 33.225 (45.303) | -1.43 | Down | 0.0929 (W) |
| TG(16:0_36:5) | 27.337 (19.792) | 30.594 (12.619) | 1.12 | Up | 0.0939 (W) |
| TG(16:1_36:5) | 2.634 (1.728) | 3.082 (1.400) | 1.17 | Up | 0.0960 (W) |
| Hypoxanthine.1 | 6.519 (4.047) | 8.720 (5.196) | 1.34 | Up | 0.0992 (W) |
| Hex3Cer(d18:1/24:1) | 0.260 (0.100) | 0.297 (0.094) | 1.14 | Up | 0.0998 |
| TG(17:1_36:5) | 0.300 (0.169) | 0.342 (0.135) | 1.14 | Up | 0.1024 (W) |
| TG(20:1_34:2) | 7.758 (3.940) | 9.515 (4.199) | 1.23 | Up | 0.1024 (W) |
| Formate | 19.070 (8.756) | 17.447 (14.176) | -1.09 | Down | 0.1024 (W) |
| TG(18:2_34:4) | 3.500 (2.400) | 4.053 (1.819) | 1.16 | Up | 0.1035 (W) |
| CE(18:2) | 1814.620 (586.160) | 2075.111 (501.775) | 1.14 | Up | 0.1046 (W) |
| TG(18:3_34:2) | 32.141 (21.711) | 36.708 (15.180) | 1.14 | Up | 0.1057 (W) |
| SM (OH) C22:1 | 12.912 (4.200) | 14.840 (3.746) | 1.15 | Up | 0.1068 (W) |
| Cer(d18:2/18:0) | 0.069 (0.022) | 0.080 (0.023) | 1.17 | Up | 0.1079 (W) |
| TG(20:0_32:3) | 1.650 (0.857) | 1.988 (0.884) | 1.21 | Up | 0.1091 (W) |
| TG(16:0_36:2) | 592.565 (298.389) | 708.000 (322.320) | 1.19 | Up | 0.1092 |
| TG(18:0_36:4) | 18.306 (15.486) | 20.137 (9.313) | 1.1 | Up | 0.1103 (W) |
| TG(17:2_38:5) | 0.268 (0.118) | 0.324 (0.127) | 1.21 | Up | 0.1103 (W) |
| GCDCA | 0.660 (0.735) | 0.778 (0.653) | 1.18 | Up | 0.1103 (W) |
| C3-DC (C4-OH) | 0.093 (0.037) | 0.105 (0.028) | 1.13 | Up | 0.1119 |
| DG(16:1_18:1) | 1.205 (0.664) | 1.509 (0.565) | 1.25 | Up | 0.1137 (W) |
| SM C16:0 | 131.098 (41.272) | 145.939 (29.926) | 1.11 | Up | 0.1162 (W) |
| Cer(d16:1/24:0) | 0.155 (0.078) | 0.188 (0.088) | 1.21 | Up | 0.1162 (W) |
| SM C16:1 | 18.521 (6.075) | 20.831 (5.005) | 1.12 | Up | 0.1174 (W) |
| TG(18:1_34:1) | 932.115 (461.629) | 1104.389 (486.730) | 1.18 | Up | 0.1177 |
| PC aa C32:1 | 29.262 (21.864) | 36.449 (22.805) | 1.25 | Up | 0.1186 (W) |
| TG(16:0_38:2) | 11.334 (5.981) | 13.509 (6.050) | 1.19 | Up | 0.1196 |
| Gly | 172.761 (82.034) | 192.144 (58.254) | 1.11 | Up | 0.1211 (W) |
| Cer(d18:1/18:0) | 0.217 (0.073) | 0.253 (0.084) | 1.17 | Up | 0.1211 (W) |
| Glu | 56.507 (27.852) | 67.731 (34.610) | 1.2 | Up | 0.122 |
| C18 | 0.031 (0.022) | 0.036 (0.023) | 1.17 | Up | 0.1258 (W) |
| TCDCA | 0.291 (0.480) | 0.379 (0.517) | 1.3 | Up | 0.1261 (W) |
| CE(22:2) | 0.163 (0.078) | 0.211 (0.111) | 1.29 | Up | 0.1272 (W) |
| TG(17:2_34:2) | 0.695 (0.330) | 0.805 (0.289) | 1.16 | Up | 0.1286 |
| TG(22:6_34:2) | 13.583 (10.981) | 15.504 (9.335) | 1.14 | Up | 0.1288 (W) |
| CE(22:6) | 59.128 (31.397) | 68.978 (23.459) | 1.17 | Up | 0.129 |
| TG(18:3_34:3) | 4.334 (3.456) | 4.701 (2.098) | 1.08 | Up | 0.1301 (W) |
| Carnitine | 12.267 (16.654) | 11.678 (8.247) | -1.05 | Down | 0.1314 (W) |
| TG(22:6_34:1) | 17.840 (17.651) | 19.196 (11.034) | 1.08 | Up | 0.1314 (W) |
| TG(18:0_36:2) | 30.506 (16.543) | 37.133 (17.628) | 1.22 | Up | 0.1355 (W) |
| PC ae C38:3 | 4.486 (1.440) | 5.040 (1.198) | 1.12 | Up | 0.1368 (W) |
| TG(20:2_34:3) | 1.348 (0.714) | 1.610 (0.682) | 1.19 | Up | 0.1368 (W) |
| TG(16:0_36:3) | 654.015 (320.314) | 765.333 (331.801) | 1.17 | Up | 0.1412 |
| C14:1 | 0.046 (0.024) | 0.052 (0.020) | 1.12 | Up | 0.1437 (W) |
| TG(22:3_30:2) | 0.044 (0.033) | 0.057 (0.035) | 1.29 | Up | 0.1439 (W) |
| HexCer(d18:1/16:0) | 1.428 (0.583) | 1.565 (0.439) | 1.1 | Up | 0.1452 (W) |
| TG(16:1_36:2) | 55.501 (28.219) | 71.917 (41.081) | 1.3 | Up | 0.1453 (W) |
| DG(18:0_20:4) | 0.047 (0.033) | 0.058 (0.030) | 1.24 | Up | 0.1453 (W) |
| SM (OH) C16:1 | 3.755 (1.361) | 4.175 (1.104) | 1.11 | Up | 0.1467 |
| DG(18:1_20:0) | 0.238 (0.160) | 0.306 (0.155) | 1.29 | Up | 0.1476 (W) |
| Arg | 71.558 (24.143) | 80.292 (17.292) | 1.12 | Up | 0.1482 (W) |
| TG(18:3_34:1) | 50.618 (26.619) | 58.439 (22.707) | 1.15 | Up | 0.1482 (W) |
| PC aa C36:3 | 192.190 (63.929) | 220.083 (53.895) | 1.15 | Up | 0.1496 (W) |
| DG(18:1_20:2) | 0.079 (0.058) | 0.102 (0.050) | 1.28 | Up | 0.1535 (W) |
| Hex2Cer(d18:1/26:0) | 0.017 (0.009) | 0.019 (0.008) | 1.14 | Up | 0.1546 (W) |
| Cer(d18:2/18:1) | 0.004 (0.002) | 0.004 (0.002) | 1.14 | Up | 0.1565 (W) |
| TG(18:1_34:4) | 6.853 (3.586) | 8.178 (3.637) | 1.19 | Up | 0.1571 (W) |
| GLCAS | 0.107 (0.145) | 0.114 (0.084) | 1.06 | Up | 0.1601 (W) |
| PC aa C32:0 | 22.061 (8.331) | 25.636 (7.744) | 1.16 | Up | 0.1602 (W) |
| TG(20:1_32:3) | 0.391 (0.185) | 0.469 (0.190) | 1.2 | Up | 0.1617 (W) |
| HexCer(d16:1/22:0) | 0.205 (0.102) | 0.240 (0.104) | 1.17 | Up | 0.1633 (W) |
| TG(17:2_38:6) | 0.236 (0.122) | 0.273 (0.100) | 1.15 | Up | 0.1647 |
| TG(22:1_32:5) | 0.079 (0.059) | 0.084 (0.039) | 1.07 | Up | 0.1680 (W) |
| CE(20:5) | 29.056 (21.197) | 34.503 (21.377) | 1.19 | Up | 0.1713 (W) |
| TG(17:1_38:5) | 0.208 (0.079) | 0.248 (0.100) | 1.2 | Up | 0.1746 (W) |
| TG(18:1_36:1) | 71.867 (36.106) | 87.747 (42.416) | 1.22 | Up | 0.1746 (W) |
| Methanol | 325.125 (217.747) | 396.767 (210.809) | 1.22 | Up | 0.1756 (W) |
| DG(18:1_22:6) | 0.316 (0.190) | 0.379 (0.137) | 1.2 | Up | 0.1761 (W) |
| TG(16:1_36:4) | 19.749 (11.244) | 23.710 (11.558) | 1.2 | Up | 0.1762 (W) |
| TG(18:0_36:3) | 40.944 (24.694) | 47.786 (21.794) | 1.17 | Up | 0.1762 (W) |
| DG(18:1_22:5) | 0.040 (0.024) | 0.050 (0.018) | 1.22 | Up | 0.1771 (W) |
| TG(18:1_34:3) | 88.857 (44.829) | 107.747 (50.520) | 1.21 | Up | 0.1779 (W) |
| PC ae C40:1 | 0.983 (0.383) | 1.157 (0.385) | 1.18 | Up | 0.1796 (W) |
| TG(17:2_36:3) | 0.562 (0.266) | 0.650 (0.247) | 1.16 | Up | 0.1796 (W) |
| Cer(d18:2/24:0) | 0.363 (0.153) | 0.410 (0.150) | 1.13 | Up | 0.1799 |
| C4 | 0.156 (0.057) | 0.207 (0.151) | 1.32 | Up | 0.1813 (W) |
| Cer(d18:2/14:0) | 0.002 (0.001) | 0.002 (0.002) | -1.24 | Down | 0.1816 (W) |
| PC aa C34:4 | 2.464 (1.215) | 3.081 (1.550) | 1.25 | Up | 0.1830 (W) |
| PC aa C36:6 | 1.031 (0.589) | 1.219 (0.596) | 1.18 | Up | 0.1830 (W) |
| Hex3Cer(d18:1_20:0) | 0.077 (0.029) | 0.086 (0.030) | 1.12 | Up | 0.1831 |
| HexCer(d18:1/14:0) | 0.028 (0.012) | 0.031 (0.013) | 1.12 | Up | 0.1842 (W) |
| PC aa C36:4 | 272.081 (93.543) | 320.833 (98.552) | 1.18 | Up | 0.1881 (W) |
| C10 | 0.391 (0.180) | 0.463 (0.152) | 1.18 | Up | 0.1898 (W) |
| Hex2Cer(d18:1/20:0) | 0.072 (0.026) | 0.080 (0.022) | 1.1 | Up | 0.1943 |
| CE(22:0) | 0.397 (0.318) | 0.504 (0.278) | 1.27 | Up | 0.1962 (W) |
| ADMA | 0.371 (0.119) | 0.412 (0.093) | 1.11 | Up | 0.1970 (W) |
| TG(16:1_36:3) | 55.100 (28.185) | 68.728 (35.750) | 1.25 | Up | 0.1989 (W) |
| TG(18:1_36:6) | 1.710 (1.521) | 1.678 (0.682) | -1.02 | Down | 0.2007 (W) |
| Cer(d16:1/23:0) | 0.111 (0.049) | 0.131 (0.061) | 1.18 | Up | 0.2025 (W) |
| SM C18:0 | 28.565 (9.836) | 31.794 (7.795) | 1.11 | Up | 0.2025 (W) |
| TG(18:2_33:2) | 5.416 (3.253) | 6.419 (3.011) | 1.19 | Up | 0.2025 (W) |
| C14:1-OH | 0.021 (0.014) | 0.026 (0.011) | 1.24 | Up | 0.2035 (W) |
| Ser | 84.681 (34.881) | 93.022 (20.912) | 1.1 | Up | 0.2056 |
| PC ae C34:0 | 1.704 (0.645) | 1.885 (0.589) | 1.11 | Up | 0.206 |
| Orn | 33.986 (16.133) | 37.428 (11.395) | 1.1 | Up | 0.2062 (W) |
| PC ae C38:0 | 1.979 (0.786) | 2.256 (0.711) | 1.14 | Up | 0.2100 (W) |
| TG(18:2_34:1) | 585.285 (284.530) | 690.889 (300.740) | 1.18 | Up | 0.2100 (W) |
| t4-OH-Pro | 11.397 (5.205) | 10.756 (5.400) | -1.06 | Down | 0.2119 (W) |
| DG(18:1_18:1) | 6.769 (3.527) | 7.816 (3.733) | 1.15 | Up | 0.2125 |
| Pyruvic acid | 99.255 (76.025) | 105.789 (56.731) | 1.07 | Up | 0.2152 (W) |
| PC aa C40:6 | 27.002 (13.400) | 30.581 (11.370) | 1.13 | Up | 0.2159 |
| Cer(d18:0/26:1(OH)) | 0.170 (0.101) | 0.207 (0.069) | 1.22 | Up | 0.2191 (W) |
| Hex2Cer(d18:1/18:0) | 0.164 (0.052) | 0.180 (0.040) | 1.1 | Up | 0.2215 (W) |
| TG(18:2_36:1) | 50.671 (29.664) | 58.994 (27.272) | 1.16 | Up | 0.2216 (W) |
| 3-IAA | 1.261 (0.851) | 1.565 (1.041) | 1.24 | Up | 0.2235 (W) |
| SM (OH) C24:1 | 0.924 (0.321) | 1.043 (0.289) | 1.13 | Up | 0.2235 (W) |
| 2-Hydroxybutyric acid | 20.980 (13.916) | 25.242 (20.273) | 1.2 | Up | 0.2275 (W) |
| CE(22:1) | 0.540 (0.323) | 0.645 (0.233) | 1.19 | Up | 0.2291 (W) |
| TMCA | 0.058 (0.070) | 0.067 (0.085) | 1.16 | Up | 0.2294 (W) |
| HexCer(d18:1/26:0) | 0.139 (0.052) | 0.152 (0.044) | 1.1 | Up | 0.2319 |
| HexCer(d18:1/24:0) | 1.662 (0.722) | 1.853 (0.657) | 1.11 | Up | 0.2332 |
| DG(18:2_18:3) | 0.794 (0.552) | 0.857 (0.374) | 1.08 | Up | 0.2397 (W) |
| Hex2Cer(d18:1/26:1) | 0.040 (0.022) | 0.045 (0.023) | 1.14 | Up | 0.2402 (W) |
| PC aa C40:3 | 0.407 (0.129) | 0.449 (0.102) | 1.1 | Up | 0.2418 (W) |
| GLCA | 0.006 (0.008) | 0.007 (0.008) | 1.33 | Up | 0.2424 (W) |
| TG(17:1_36:4) | 1.548 (0.914) | 1.769 (0.807) | 1.14 | Up | 0.2460 (W) |
| TG(18:2_34:2) | 380.498 (223.937) | 440.417 (211.494) | 1.16 | Up | 0.2460 (W) |
| TG(18:1_35:2) | 13.458 (6.472) | 16.162 (7.867) | 1.2 | Up | 0.2503 (W) |
| DG(14:0_20:0) | 0.052 (0.034) | 0.063 (0.027) | 1.2 | Up | 0.2582 (W) |
| Met-SO | 0.287 (0.336) | 0.352 (0.331) | 1.23 | Up | 0.2611 (W) |
| TG(16:0_36:4) | 248.513 (145.085) | 287.033 (135.815) | 1.16 | Up | 0.2656 (W) |
| TG(18:3_36:1) | 5.219 (3.811) | 5.494 (2.328) | 1.05 | Up | 0.2679 (W) |
| PC aa C38:6 | 112.016 (50.191) | 124.031 (43.212) | 1.11 | Up | 0.2696 |
| TG(18:2_34:3) | 45.872 (30.271) | 52.064 (25.390) | 1.13 | Up | 0.2701 (W) |
| TCA | 0.293 (0.604) | 0.478 (0.989) | 1.63 | Up | 0.2701 (W) |
| TG(18:1_35:3) | 3.767 (1.879) | 4.483 (2.103) | 1.19 | Up | 0.2747 (W) |
| TG(17:2_36:4) | 1.118 (0.564) | 1.253 (0.496) | 1.12 | Up | 0.2764 |
| Isopropyl alcohol | 12.018 (10.140) | 8.989 (6.501) | -1.34 | Down | 0.2769 (W) |
| PC ae C30:1 | 0.171 (0.109) | 0.200 (0.126) | 1.17 | Up | 0.2807 |
| TG(18:2_36:4) | 53.705 (59.907) | 52.522 (28.667) | -1.02 | Down | 0.2839 (W) |
| PC ae C34:2 | 16.507 (5.658) | 16.171 (4.752) | -1.02 | Down | 0.2862 (W) |
| SM C18:1 | 12.945 (4.754) | 14.315 (3.995) | 1.11 | Up | 0.2886 (W) |
| DG(18:1_18:4) | 0.056 (0.050) | 0.069 (0.056) | 1.23 | Up | 0.2890 (W) |
| Hex2Cer(d18:1/14:0) | 0.279 (0.125) | 0.307 (0.098) | 1.1 | Up | 0.2903 |
| DG(18:1_18:2) | 17.124 (8.278) | 19.961 (9.248) | 1.17 | Up | 0.2982 (W) |
| Cer(d18:1/23:0) | 0.992 (0.370) | 1.069 (0.288) | 1.08 | Up | 0.3129 |
| Hex3Cer(d18:1_22:0) | 0.215 (0.080) | 0.240 (0.088) | 1.12 | Up | 0.3154 (W) |
| PC aa C34:1 | 305.933 (114.078) | 334.056 (75.923) | 1.09 | Up | 0.3154 (W) |
| SM C26:0 | 0.102 (0.037) | 0.110 (0.032) | 1.08 | Up | 0.3175 |
| Serotonin | 0.521 (0.372) | 0.443 (0.323) | -1.18 | Down | 0.3178 (W) |
| Gln | 433.315 (144.416) | 474.306 (123.084) | 1.09 | Up | 0.3204 (W) |
| PC ae C36:5 | 11.563 (4.691) | 12.625 (4.692) | 1.09 | Up | 0.3276 |
| DG(18:1_18:3) | 1.144 (0.488) | 1.254 (0.487) | 1.1 | Up | 0.3294 |
| DG(18:2_18:4) | 0.070 (0.043) | 0.080 (0.044) | 1.13 | Up | 0.3307 (W) |
| Citric acid | 59.212 (29.784) | 58.842 (53.416) | -1.01 | Down | 0.3333 (W) |
| Cys | 88.805 (65.665) | 89.386 (33.349) | 1.01 | Up | 0.3359 (W) |
| TG(18:2_36:3) | 121.073 (97.658) | 128.064 (65.741) | 1.06 | Up | 0.3385 (W) |
| TG(18:3_35:2) | 0.938 (0.616) | 1.023 (0.464) | 1.09 | Up | 0.3385 (W) |
| PC aa C36:5 | 15.601 (7.722) | 18.956 (10.299) | 1.22 | Up | 0.3411 (W) |
| TG(18:2_35:2) | 7.490 (4.267) | 8.525 (4.203) | 1.14 | Up | 0.3438 (W) |
| PC aa C42:4 | 0.227 (0.080) | 0.254 (0.077) | 1.12 | Up | 0.3464 (W) |
| DG(17:0_17:1) | 0.105 (0.070) | 0.125 (0.050) | 1.19 | Up | 0.3479 (W) |
| PC ae C44:4 | 0.441 (0.179) | 0.409 (0.103) | -1.08 | Down | 0.3486 |
| TG(18:2_35:1) | 9.319 (4.697) | 10.842 (5.217) | 1.16 | Up | 0.3518 (W) |
| PC aa C42:6 | 0.471 (0.183) | 0.527 (0.173) | 1.12 | Up | 0.3518 (W) |
| TG(17:0_36:4) | 3.100 (1.961) | 3.569 (1.819) | 1.15 | Up | 0.3518 (W) |
| TG(18:1_36:4) | 92.085 (66.361) | 99.108 (47.512) | 1.08 | Up | 0.3518 (W) |
| PC ae C34:3 | 10.131 (4.214) | 9.553 (3.993) | -1.06 | Down | 0.3545 (W) |
| TG(18:1_36:2) | 242.677 (127.831) | 286.081 (144.964) | 1.18 | Up | 0.3572 (W) |
| beta-Ala | 2.631 (3.346) | 3.020 (3.451) | 1.15 | Up | 0.3626 (W) |
| TG(20:0_32:4) | 1.817 (1.089) | 2.058 (0.997) | 1.13 | Up | 0.3654 (W) |
| PC ae C38:2 | 1.840 (0.668) | 1.966 (0.510) | 1.07 | Up | 0.366 |
| Hex2Cer(d18:1/22:0) | 0.176 (0.078) | 0.188 (0.052) | 1.07 | Up | 0.3681 (W) |
| Cer(d18:1/16:0) | 0.533 (0.175) | 0.571 (0.133) | 1.07 | Up | 0.3737 (W) |
| PC ae C36:1 | 8.692 (3.161) | 9.277 (2.470) | 1.07 | Up | 0.3754 |
| Cer(d18:1/24:0) | 2.343 (0.909) | 2.513 (0.734) | 1.07 | Up | 0.3783 |
| TG(18:2_36:2) | 136.142 (83.051) | 152.208 (73.956) | 1.12 | Up | 0.3936 (W) |
| TG(17:1_36:3) | 3.482 (1.687) | 4.093 (2.038) | 1.18 | Up | 0.3936 (W) |
| PC aa C38:0 | 2.917 (1.313) | 2.807 (0.993) | -1.04 | Down | 0.3965 (W) |
| HexCer(d18:2/18:0) | 0.039 (0.016) | 0.044 (0.016) | 1.14 | Up | 0.4020 (W) |
| TG(17:1_38:6) | 0.164 (0.073) | 0.180 (0.064) | 1.1 | Up | 0.4052 (W) |
| CDCA | 0.239 (0.233) | 0.270 (0.214) | 1.13 | Up | 0.4060 (W) |
| Cer(d18:1/24:1) | 1.123 (0.394) | 1.229 (0.343) | 1.09 | Up | 0.4081 (W) |
| PC ae C40:5 | 3.423 (1.211) | 3.663 (0.895) | 1.07 | Up | 0.4082 (W) |
| TrpBetaine | 0.448 (0.519) | 0.513 (0.533) | 1.14 | Up | 0.4141 (W) |
| Cer(d18:1/22:0) | 0.947 (0.323) | 1.033 (0.271) | 1.09 | Up | 0.4261 (W) |
| Asn | 49.678 (26.807) | 51.511 (18.007) | 1.04 | Up | 0.4261 (W) |
| TG(18:1_36:3) | 229.242 (128.463) | 260.564 (128.497) | 1.14 | Up | 0.4261 (W) |
| Cer(d18:1/18:1) | 0.025 (0.012) | 0.022 (0.012) | -1.12 | Down | 0.4281 (W) |
| Cer(d18:2/22:0) | 0.231 (0.086) | 0.252 (0.085) | 1.09 | Up | 0.4383 (W) |
| DG(18:2_18:2) | 10.689 (6.177) | 11.916 (6.023) | 1.11 | Up | 0.4383 (W) |
| Ala | 403.870 (207.133) | 418.333 (140.850) | 1.04 | Up | 0.4445 (W) |
| Cer(d18:2/24:1) | 0.175 (0.065) | 0.196 (0.069) | 1.12 | Up | 0.4476 (W) |
| PC ae C36:3 | 9.223 (3.187) | 9.107 (2.273) | -1.01 | Down | 0.4507 (W) |
| HexCer(d18:1/20:0) | 0.373 (0.159) | 0.406 (0.146) | 1.09 | Up | 0.4507 (W) |
| TG(17:0_36:3) | 8.104 (4.054) | 9.446 (4.743) | 1.17 | Up | 0.4507 (W) |
| C16:1 | 0.018 (0.019) | 0.021 (0.019) | 1.16 | Up | 0.4566 (W) |
| DCA | 0.235 (0.228) | 0.257 (0.194) | 1.09 | Up | 0.4584 (W) |
| HexCer(d18:1/23:0) | 1.780 (0.766) | 1.889 (0.631) | 1.06 | Up | 0.4601 (W) |
| Val | 94.677 (48.018) | 102.072 (44.717) | 1.08 | Up | 0.4665 (W) |
| PC ae C38:1 | 0.104 (0.184) | 0.045 (0.074) | -2.29 | Down | 0.4694 (W) |
| C2 | 2.649 (1.621) | 2.658 (1.651) | 1 | Up | 0.4696 (W) |
| Met | 22.346 (10.177) | 23.708 (7.611) | 1.06 | Up | 0.4696 (W) |
| Kynurenine | 1.484 (0.561) | 1.570 (0.379) | 1.06 | Up | 0.4728 (W) |
| Cer(d18:2/23:0) | 0.116 (0.051) | 0.125 (0.051) | 1.07 | Up | 0.4728 (W) |
| PC ae C32:2 | 0.821 (0.276) | 0.889 (0.237) | 1.08 | Up | 0.4825 (W) |
| DG(18:3_18:3) | 0.037 (0.048) | 0.044 (0.044) | 1.18 | Up | 0.4854 (W) |
| 1-Met-His | 2.958 (1.466) | 3.471 (1.852) | 1.17 | Up | 0.4858 (W) |
| lysoPC a C26:1 | 0.110 (0.055) | 0.115 (0.045) | 1.05 | Up | 0.4890 (W) |
| Trp | 39.333 (14.059) | 43.703 (12.095) | 1.11 | Up | 0.4890 (W) |
| PC ae C30:0 | 0.378 (0.147) | 0.423 (0.158) | 1.12 | Up | 0.4890 (W) |
| BABA | 0.028 (0.032) | 0.031 (0.047) | 1.12 | Up | 0.4896 (W) |
| PC aa C42:2 | 0.194 (0.068) | 0.204 (0.048) | 1.05 | Up | 0.4921 |
| Sarcosine | 0.518 (0.465) | 0.622 (0.495) | 1.2 | Up | 0.5016 (W) |
| Cer(d18:0/24:0) | 0.240 (0.118) | 0.263 (0.079) | 1.1 | Up | 0.5020 (W) |
| TG(18:3_36:3) | 16.561 (15.896) | 15.813 (7.781) | -1.05 | Down | 0.5022 (W) |
| Cer(d18:2/16:0) | 0.108 (0.038) | 0.118 (0.037) | 1.09 | Up | 0.5055 (W) |
| HexCer(d18:1/18:0) | 0.254 (0.101) | 0.281 (0.114) | 1.11 | Up | 0.5055 (W) |
| Trigonelline | 0.418 (0.458) | 0.537 (1.131) | 1.28 | Up | 0.5055 (W) |
| TG(18:2_36:5) | 10.707 (14.796) | 9.018 (5.185) | -1.19 | Down | 0.5122 (W) |
| Choline | 3.562 (1.757) | 3.943 (1.970) | 1.11 | Up | 0.5220 (W) |
| FA(20:2) | 0.972 (1.423) | 0.896 (1.393) | -1.09 | Down | 0.5255 (W) |
| D-Glucose | 2649.980 (1005.295) | 2978.611 (1311.496) | 1.12 | Up | 0.5312 (W) |
| DG-O(16:0_20:4) | 0.005 (0.005) | 0.004 (0.004) | -1.19 | Down | 0.5320 (W) |
| TG(18:1_36:5) | 16.913 (15.384) | 16.301 (7.676) | -1.04 | Down | 0.5359 (W) |
| TG(18:3_36:4) | 7.801 (10.705) | 6.624 (3.797) | -1.18 | Down | 0.5359 (W) |
| HArg | 4.631 (2.173) | 4.954 (2.405) | 1.07 | Up | 0.5407 |
| Hex2Cer(d18:1/24:0) | 0.128 (0.049) | 0.134 (0.039) | 1.05 | Up | 0.5421 |
| HexCer(d18:2/24:0) | 0.730 (0.317) | 0.804 (0.333) | 1.1 | Up | 0.5462 (W) |
| PC ae C40:3 | 1.043 (0.348) | 1.106 (0.224) | 1.06 | Up | 0.5496 (W) |
| Lys | 164.540 (59.458) | 172.161 (51.147) | 1.05 | Up | 0.5531 |
| PC aa C36:1 | 53.802 (21.940) | 57.989 (17.772) | 1.08 | Up | 0.5532 (W) |
| Cer(d18:2/20:0) | 0.073 (0.027) | 0.076 (0.022) | 1.05 | Up | 0.555 |
| Creatinine.1 | 49.872 (15.949) | 53.189 (10.813) | 1.07 | Up | 0.5567 (W) |
| 3-Met-His | 5.157 (6.227) | 5.385 (6.596) | 1.04 | Up | 0.5602 (W) |
| AABA | 11.563 (7.016) | 11.197 (7.368) | -1.03 | Down | 0.5637 (W) |
| Cer(d18:0/26:1) | 0.010 (0.010) | 0.012 (0.010) | 1.14 | Up | 0.5799 (W) |
| HexCer(d18:1/22:0) | 3.682 (1.515) | 3.908 (1.509) | 1.06 | Up | 0.5814 (W) |
| PC aa C36:0 | 1.337 (0.715) | 1.283 (0.788) | -1.04 | Down | 0.5848 (W) |
| PC aa C30:2 | 0.140 (0.086) | 0.151 (0.093) | 1.08 | Up | 0.5874 |
| Hypoxanthine | 0.089 (0.036) | 0.090 (0.066) | 1.02 | Up | 0.5924 (W) |
| Thr | 191.042 (84.983) | 194.778 (86.293) | 1.02 | Up | 0.6066 (W) |
| Cer(d18:1/25:0) | 0.375 (0.150) | 0.391 (0.129) | 1.04 | Up | 0.6187 |
| TG(18:2_35:3) | 2.271 (1.397) | 2.460 (1.241) | 1.08 | Up | 0.6212 (W) |
| HexCer(d18:2/23:0) | 0.280 (0.117) | 0.307 (0.132) | 1.1 | Up | 0.6359 (W) |
| PC ae C36:4 | 19.691 (7.251) | 20.407 (5.699) | 1.04 | Up | 0.6363 |
| PC aa C38:1 | 0.755 (0.661) | 0.650 (0.548) | -1.16 | Down | 0.6428 (W) |
| Cer(d18:1/18:0(OH)) | 0.090 (0.056) | 0.077 (0.057) | -1.16 | Down | 0.6441 (W) |
| C5 | 0.109 (0.037) | 0.114 (0.055) | 1.04 | Up | 0.6508 (W) |
| Cit | 17.800 (7.802) | 19.082 (6.643) | 1.07 | Up | 0.6546 (W) |
| Xanthine | 0.971 (0.789) | 1.091 (0.944) | 1.12 | Up | 0.6689 (W) |
| PC aa C42:0 | 0.673 (0.295) | 0.649 (0.192) | -1.04 | Down | 0.6765 |
| H1 | 4649.670 (1691.653) | 4908.861 (1103.415) | 1.06 | Up | 0.6811 (W) |
| C0 | 15.809 (6.197) | 16.739 (7.319) | 1.06 | Up | 0.6887 (W) |
| Lac | 4589.180 (6391.564) | 3486.194 (1140.909) | -1.32 | Down | 0.6888 (W) |
| PC ae C36:0 | 0.735 (0.264) | 0.755 (0.172) | 1.03 | Up | 0.6925 |
| lysoPC a C28:0 | 0.167 (0.140) | 0.160 (0.170) | -1.04 | Down | 0.6938 (W) |
| Cer(d18:1/26:0) | 0.051 (0.020) | 0.053 (0.014) | 1.03 | Up | 0.7066 |
| TG(18:3_36:2) | 18.527 (12.843) | 18.976 (8.478) | 1.02 | Up | 0.7119 (W) |
| FA(18:1) | 120.075 (122.166) | 109.239 (112.146) | -1.1 | Down | 0.7150 (W) |
| GUDCA | 0.059 (0.098) | 0.047 (0.051) | -1.27 | Down | 0.7196 (W) |
| PC ae C42:4 | 1.035 (0.407) | 1.007 (0.271) | -1.03 | Down | 0.7228 |
| Hex3Cer(d18:1/18:0) | 0.163 (0.069) | 0.167 (0.044) | 1.03 | Up | 0.7274 (W) |
| Taurine | 49.340 (29.823) | 52.842 (30.703) | 1.07 | Up | 0.7314 (W) |
| HipAcid | 2.210 (2.735) | 2.691 (3.855) | 1.22 | Up | 0.7314 (W) |
| PC ae C38:4 | 15.329 (5.367) | 16.305 (4.652) | 1.06 | Up | 0.7392 (W) |
| L-Lactic acid | 2276.850 (3101.896) | 1882.097 (819.197) | -1.21 | Down | 0.7603 (W) |
| Ind-SO4 | 3.080 (2.424) | 3.220 (2.255) | 1.05 | Up | 0.7708 (W) |
| Creatinine | 27.855 (11.946) | 29.236 (12.202) | 1.05 | Up | 0.7788 (W) |
| HexCer(d18:2/22:0) | 0.598 (0.264) | 0.630 (0.272) | 1.05 | Up | 0.7908 (W) |
| PC ae C40:2 | 1.714 (0.620) | 1.797 (0.495) | 1.05 | Up | 0.7948 (W) |
| PC ae C44:5 | 1.607 (0.673) | 1.574 (0.434) | -1.02 | Down | 0.7979 |
| PC ae C44:3 | 0.151 (0.060) | 0.148 (0.038) | -1.02 | Down | 0.8 |
| TMAO | 2.788 (2.033) | 3.359 (4.698) | 1.21 | Up | 0.8069 (W) |
| PC aa C40:1 | 0.228 (0.198) | 0.224 (0.185) | -1.02 | Down | 0.8118 (W) |
| HexCer(d18:1/18:1) | 0.068 (0.069) | 0.063 (0.066) | -1.07 | Down | 0.8194 (W) |
| Leu | 84.445 (38.176) | 85.897 (25.368) | 1.02 | Up | 0.8271 (W) |
| 3-IPA | 0.571 (1.085) | 0.436 (0.439) | -1.31 | Down | 0.8309 (W) |
| PC aa C40:2 | 0.267 (0.094) | 0.263 (0.065) | -1.01 | Down | 0.8314 |
| AconAcid | 2.127 (1.296) | 2.306 (1.698) | 1.08 | Up | 0.8352 (W) |
| PC aa C36:2 | 260.775 (80.256) | 275.500 (59.431) | 1.06 | Up | 0.8352 (W) |
| PC ae C40:6 | 4.888 (2.025) | 4.973 (1.471) | 1.02 | Up | 0.8367 |
| Hex2Cer(d18:1/24:1) | 0.301 (0.111) | 0.306 (0.097) | 1.02 | Up | 0.8418 |
| PC aa C34:2 | 593.740 (170.368) | 631.222 (94.460) | 1.06 | Up | 0.8433 (W) |
| DHEAS | 1.484 (1.189) | 1.594 (1.555) | 1.07 | Up | 0.8433 (W) |
| PC aa C42:1 | 0.343 (0.152) | 0.337 (0.106) | -1.02 | Down | 0.8445 |
| Asp | 15.030 (5.120) | 16.042 (6.712) | 1.07 | Up | 0.8474 (W) |
| PC ae C36:2 | 18.193 (5.962) | 18.850 (5.480) | 1.04 | Up | 0.8514 (W) |
| AA | 3.612 (2.451) | 3.937 (3.733) | 1.09 | Up | 0.8540 (W) |
| PC ae C42:5 | 2.061 (0.783) | 2.086 (0.481) | 1.01 | Up | 0.8555 (W) |
| PC ae C32:1 | 3.578 (1.231) | 3.746 (1.028) | 1.05 | Up | 0.8555 (W) |
| Hex2Cer(d18:1/16:0) | 3.261 (1.147) | 3.304 (0.972) | 1.01 | Up | 0.8625 |
| alpha-AAA | 0.891 (0.470) | 0.967 (0.619) | 1.09 | Up | 0.8637 (W) |
| Betaine.1 | 15.827 (5.487) | 15.697 (3.584) | -1.01 | Down | 0.8678 (W) |
| C3 | 0.236 (0.081) | 0.242 (0.063) | 1.03 | Up | 0.8678 (W) |
| CE(20:0) | 1.452 (0.860) | 1.704 (2.365) | 1.17 | Up | 0.8737 (W) |
| Ile | 51.385 (24.255) | 51.425 (15.724) | 1 | Up | 0.8760 (W) |
| PC ae C34:1 | 13.698 (4.947) | 14.285 (3.444) | 1.04 | Up | 0.8842 (W) |
| Urea | 130.165 (149.485) | 113.583 (130.120) | -1.15 | Down | 0.8842 (W) |
| DHA | 3.068 (2.335) | 3.393 (2.867) | 1.11 | Up | 0.8842 (W) |
| PC ae C38:6 | 7.282 (2.871) | 7.489 (2.512) | 1.03 | Up | 0.8842 (W) |
| Succinate | 12.965 (4.736) | 14.186 (8.111) | 1.09 | Up | 0.8883 (W) |
| lysoPC a C26:0 | 0.186 (0.093) | 0.182 (0.069) | -1.02 | Down | 0.9171 (W) |
| PC ae C38:5 | 17.735 (6.144) | 18.652 (5.133) | 1.05 | Up | 0.9213 (W) |
| PC ae C42:3 | 0.897 (0.358) | 0.891 (0.253) | -1.01 | Down | 0.9273 |
| Betaine | 9.421 (6.173) | 8.988 (4.926) | -1.05 | Down | 0.9337 (W) |
| His | 95.382 (45.270) | 92.681 (27.911) | -1.03 | Down | 0.9461 (W) |
| Cer(d18:0/22:0) | 0.124 (0.073) | 0.126 (0.073) | 1.02 | Up | 0.9580 (W) |
| FA(18:2) | 152.798 (149.043) | 138.522 (116.848) | -1.1 | Down | 0.9585 (W) |
| PC ae C40:4 | 2.692 (0.929) | 2.783 (0.631) | 1.03 | Up | 0.9585 (W) |
| SDMA | 0.552 (0.207) | 0.557 (0.130) | 1.01 | Up | 0.9710 (W) |
| HexCer(d18:1/24:1) | 4.032 (1.671) | 4.143 (1.292) | 1.03 | Up | 0.9710 (W) |
| PC ae C42:2 | 0.473 (0.187) | 0.481 (0.125) | 1.02 | Up | 0.9751 (W) |
| Cer(d18:0/24:1) | 0.184 (0.079) | 0.184 (0.063) | 1 | Up | 0.9792 (W) |
| PC ae C44:6 | 1.486 (0.598) | 1.487 (0.462) | 1 | Up | 0.9905 |
| 1-Methylhistidine | 97.597 (39.668) | 97.525 (37.747) | -1 | Down | 0.9935 |
| Cortisol | 0.468 (0.312) | 0.468 (0.315) | 1 | Up | 0.9978 |

**Supplementary Table S8. Univariate analysis of metabolites and lipids, late vs controls**

| Name | Mean (SD) of Controls | Mean (SD) of Cases | Fold Change | 0/1 | p.value.origin |
| --- | --- | --- | --- | --- | --- |
| SM C22:3 | 0.668 (0.475) | 1.066 (0.620) | -1.6 | Up | **0.00183** |
| DG(18:1_20:1) | 0.215 (0.083) | 0.263 (0.090) | -1.22 | Up | **0.01564** |
| Hex3Cer(d18:1_22:0) | 0.215 (0.080) | 0.259 (0.087) | -1.2 | Up | **0.02066** |
| Hex3Cer(d18:1/24:1) | 0.260 (0.099) | 0.312 (0.097) | -1.2 | Up | **0.02194** |
| SM C20:2 | 0.643 (0.264) | 0.780 (0.266) | -1.21 | Up | **0.02357** |
| CE(18:2) | 1814.620 (586.160) | 2072.835 (540.307) | -1.14 | Up | **0.02526** |
| HexCer(d18:1/18:0) | 0.254 (0.100) | 0.302 (0.095) | -1.19 | Up | **0.03011** |
| Cer(d18:1/20:0(OH)) | 1.492 (0.627) | 1.773 (0.455) | -1.19 | Up | **0.03223** |
| PC ae C40:3 | 1.043 (0.348) | 1.187 (0.317) | -1.14 | Up | **0.03263** |
| Cer(d18:1/18:0) | 0.217 (0.073) | 0.249 (0.065) | -1.15 | Up | **0.03677** |
| Cer(d16:1/18:0) | 0.081 (0.033) | 0.096 (0.034) | -1.19 | Up | **0.0463** |
| PC ae C38:2 | 1.840 (0.668) | 2.136 (0.641) | -1.16 | Up | **0.04697** |
| PC aa C28:1 | 3.630 (1.313) | 4.192 (1.187) | -1.15 | Up | **0.04824** |
| SM (OH) C14:1 | 6.745 (2.363) | 7.762 (2.177) | -1.15 | Up | **0.04864** |
| CE(20:0) | 1.452 (0.860) | 1.933 (1.025) | -1.33 | Up | **0.04984** |
| HexCer(d16:1/24:0) | 0.093 (0.043) | 0.111 (0.039) | -1.19 | Up | 0.05071 |
| C12 | 0.092 (0.069) | 0.121 (0.071) | -1.32 | Up | 0.05599 |
| SM C26:0 | 0.102 (0.037) | 0.118 (0.039) | -1.16 | Up | 0.06596 |
| Hex3Cer(d18:1_20:0) | 0.077 (0.029) | 0.088 (0.025) | -1.15 | Up | 0.06686 |
| TMCA | 0.058 (0.070) | 0.071 (0.062) | -1.23 | Up | 0.07112 |
| SM C24:1 | 42.047 (14.450) | 46.935 (11.458) | -1.12 | Up | 0.07116 |
| Hex3Cer(d18:1/16:0) | 1.192 (0.412) | 1.336 (0.366) | -1.12 | Up | 0.07822 |
| HexCer(d18:1/16:0) | 1.428 (0.583) | 1.594 (0.472) | -1.12 | Up | 0.07902 |
| PC aa C42:4 | 0.227 (0.080) | 0.259 (0.079) | -1.14 | Up | 0.07962 |
| TCDCA | 0.291 (0.480) | 0.402 (0.620) | -1.38 | Up | 0.08319 |
| Cer(d18:2/14:0) | 0.002 (0.001) | 0.003 (0.001) | -1.25 | Up | 0.0837 |
| Cer(d16:1/23:0) | 0.112 (0.049) | 0.131 (0.051) | -1.17 | Up | 0.08718 |
| TG(18:1_26:0) | 2.932 (3.662) | 4.522 (6.795) | -1.54 | Up | 0.09032 |
| Hex2Cer(d18:1/18:0) | 0.164 (0.052) | 0.185 (0.044) | -1.13 | Up | 0.09492 |
| SM C16:0 | 131.098 (41.272) | 145.204 (34.416) | -1.11 | Up | 0.09493 |
| HArg | 4.630 (2.176) | 3.936 (1.418) | 1.18 | Down | 0.0957 |
| CE(20:3) | 37.911 (15.089) | 43.713 (16.044) | -1.15 | Up | 0.09971 |
| SM C26:1 | 0.217 (0.078) | 0.245 (0.068) | -1.12 | Up | 0.1016 |
| DG(16:0_20:4) | 0.070 (0.038) | 0.081 (0.036) | -1.16 | Up | 0.10523 |
| PC ae C40:1 | 0.983 (0.383) | 1.118 (0.363) | -1.14 | Up | 0.11116 |
| Hex3Cer(d18:1/26:1) | 0.226 (0.094) | 0.261 (0.103) | -1.16 | Up | 0.11305 |
| GCA | 0.375 (0.467) | 0.494 (0.479) | -1.32 | Up | 0.1145 |
| Cer(d18:0/22:0) | 0.124 (0.073) | 0.154 (0.064) | -1.25 | Up | 0.1161 |
| SM C18:0 | 28.565 (9.836) | 31.531 (7.285) | -1.1 | Up | 0.12128 |
| TCA | 0.293 (0.604) | 0.575 (1.755) | -1.96 | Up | 0.12246 |
| SM (OH) C16:1 | 3.755 (1.361) | 4.139 (1.029) | -1.1 | Up | 0.12246 |
| C10 | 0.390 (0.182) | 0.459 (0.158) | -1.18 | Up | 0.12474 |
| PC aa C40:3 | 0.407 (0.129) | 0.449 (0.115) | -1.1 | Up | 0.13077 |
| ProBetaine | 3.834 (5.678) | 6.422 (9.824) | -1.68 | Up | 0.13196 |
| PC ae C36:2 | 18.193 (5.962) | 19.755 (4.879) | -1.09 | Up | 0.13205 |
| Hex3Cer(d18:1/18:0) | 0.163 (0.069) | 0.182 (0.058) | -1.12 | Up | 0.13452 |
| TG(22:2_32:4) | 0.163 (0.151) | 0.170 (0.082) | -1.04 | Up | 0.13579 |
| TG(22:0_32:4) | 0.145 (0.091) | 0.173 (0.066) | -1.19 | Up | 0.13681 |
| PC aa C34:2 | 593.740 (170.368) | 650.170 (136.448) | -1.1 | Up | 0.13836 |
| Tyr | 41.170 (16.709) | 47.780 (19.668) | -1.16 | Up | 0.13964 |
| HexCer(d18:1/26:1) | 0.091 (0.033) | 0.102 (0.033) | -1.12 | Up | 0.14023 |
| HexCer(d18:1/26:0) | 0.139 (0.052) | 0.155 (0.048) | -1.12 | Up | 0.14094 |
| Cer(d18:1/24:1) | 1.123 (0.394) | 1.223 (0.312) | -1.09 | Up | 0.14214 |
| CE(16:0) | 270.230 (88.907) | 298.540 (76.002) | -1.1 | Up | 0.14223 |
| HipAcid | 2.209 (2.735) | 1.357 (0.983) | 1.63 | Down | 0.14224 |
| Cer(d18:0/24:1) | 0.184 (0.079) | 0.207 (0.063) | -1.12 | Up | 0.14482 |
| CE(20:4) | 310.895 (135.089) | 353.860 (125.797) | -1.14 | Up | 0.14502 |
| HexCer(d18:1/24:0) | 1.662 (0.722) | 1.881 (0.602) | -1.13 | Up | 0.14565 |
| C9 | 0.031 (0.014) | 0.035 (0.016) | -1.16 | Up | 0.14854 |
| Cer(d16:1/20:0) | 0.054 (0.022) | 0.065 (0.025) | -1.2 | Up | 0.15006 |
| Cer(d18:1/16:0) | 0.533 (0.175) | 0.581 (0.143) | -1.09 | Up | 0.15298 |
| TG(17:1_38:7) | 0.034 (0.022) | 0.039 (0.018) | -1.16 | Up | 0.15395 |
| TG(20:4_35:3) | 0.140 (0.075) | 0.157 (0.067) | -1.12 | Up | 0.15437 |
| C4 | 0.156 (0.057) | 0.176 (0.067) | -1.13 | Up | 0.15558 |
| C0 | 15.809 (6.197) | 15.425 (8.098) | 1.02 | Down | 0.15576 |
| Cortisol | 0.468 (0.312) | 0.575 (0.354) | -1.23 | Up | 0.15584 |
| Cer(d18:1/14:0) | 0.069 (0.030) | 0.081 (0.031) | -1.17 | Up | 0.1571 |
| HexCer(d18:1/22:0) | 3.683 (1.515) | 4.038 (1.223) | -1.1 | Up | 0.15719 |
| TG(18:2_28:0) | 6.660 (6.578) | 8.285 (6.855) | -1.24 | Up | 0.1572 |
| TG(22:3_30:2) | 0.044 (0.033) | 0.053 (0.036) | -1.21 | Up | 0.15934 |
| PC aa C30:0 | 5.463 (2.677) | 6.262 (2.665) | -1.15 | Up | 0.16004 |
| TG(18:0_32:0) | 13.389 (8.903) | 15.648 (8.687) | -1.17 | Up | 0.16143 |
| TG(20:2_34:2) | 7.608 (4.165) | 8.865 (4.233) | -1.17 | Up | 0.16292 |
| Malonate | 73.623 (324.584) | 42.005 (245.382) | 1.75 | Down | 0.16417 |
| FA(20:2) | 0.949 (1.433) | 1.248 (1.312) | -1.31 | Up | 0.16526 |
| SM (OH) C22:1 | 12.912 (4.200) | 14.135 (3.867) | -1.09 | Up | 0.16726 |
| p-Cresol-SO4 | 14.579 (9.768) | 19.033 (13.089) | -1.31 | Up | 0.16731 |
| CE(20:1) | 1.181 (0.401) | 1.315 (0.428) | -1.11 | Up | 0.16867 |
| PC aa C32:2 | 7.591 (3.458) | 8.712 (3.760) | -1.15 | Up | 0.16916 |
| TG(16:0_28:2) | 1.732 (1.974) | 2.289 (2.509) | -1.32 | Up | 0.17029 |
| TG(14:0_36:4) | 15.310 (9.605) | 18.169 (10.273) | -1.19 | Up | 0.17175 |
| C18:1 | 0.041 (0.051) | 0.050 (0.044) | -1.23 | Up | 0.17212 |
| FA(18:1) | 120.075 (122.166) | 161.928 (141.082) | -1.35 | Up | 0.17567 |
| FA(20:1) | 1.704 (2.059) | 2.493 (2.698) | -1.46 | Up | 0.1762 |
| PC ae C40:2 | 1.714 (0.620) | 1.833 (0.452) | -1.07 | Up | 0.17633 |
| TG(18:2_36:0) | 5.907 (3.901) | 6.477 (2.894) | -1.1 | Up | 0.17637 |
| HexCer(d18:2/20:0) | 0.076 (0.034) | 0.085 (0.026) | -1.12 | Up | 0.1769 |
| C14:1 | 0.046 (0.024) | 0.051 (0.021) | -1.11 | Up | 0.18245 |
| Cer(d18:0/24:0) | 0.240 (0.118) | 0.277 (0.091) | -1.16 | Up | 0.18725 |
| TG(18:2_32:2) | 28.392 (17.392) | 33.370 (18.110) | -1.18 | Up | 0.18739 |
| TG(18:2_31:0) | 3.931 (2.471) | 4.791 (2.609) | -1.22 | Up | 0.1887 |
| Hex2Cer(d18:1/14:0) | 0.279 (0.125) | 0.315 (0.116) | -1.13 | Up | 0.19011 |
| PC ae C42:1 | 0.365 (0.123) | 0.401 (0.109) | -1.1 | Up | 0.19063 |
| TG(20:1_32:3) | 0.391 (0.185) | 0.462 (0.227) | -1.18 | Up | 0.19556 |
| DG(17:0_18:1) | 0.655 (0.244) | 0.729 (0.271) | -1.11 | Up | 0.20351 |
| Choline | 3.562 (1.757) | 4.143 (2.228) | -1.16 | Up | 0.20386 |
| C14:2 | 0.043 (0.034) | 0.052 (0.036) | -1.23 | Up | 0.20703 |
| TG(18:1_28:1) | 2.721 (2.104) | 3.605 (4.107) | -1.32 | Up | 0.20745 |
| FA(18:2) | 152.798 (149.043) | 178.958 (136.140) | -1.17 | Up | 0.20917 |
| HexCer(d18:2/24:0) | 0.730 (0.317) | 0.795 (0.264) | -1.09 | Up | 0.20918 |
| HexCer(d18:1/23:0) | 1.780 (0.766) | 1.954 (0.627) | -1.1 | Up | 0.21091 |
| DCA | 0.235 (0.228) | 0.166 (0.162) | 1.42 | Down | 0.21496 |
| TG(18:2_30:0) | 35.458 (25.942) | 41.534 (26.476) | -1.17 | Up | 0.21624 |
| TG(16:0_36:5) | 27.337 (19.792) | 30.045 (15.830) | -1.1 | Up | 0.21805 |
| PC aa C36:2 | 260.775 (80.256) | 285.925 (69.819) | -1.1 | Up | 0.21984 |
| TG(20:1_34:2) | 7.758 (3.940) | 9.148 (4.628) | -1.18 | Up | 0.22165 |
| TG(18:0_36:5) | 2.654 (2.941) | 2.603 (1.476) | 1.02 | Down | 0.22348 |
| PC ae C36:0 | 0.735 (0.264) | 0.802 (0.230) | -1.09 | Up | 0.22509 |
| TG(20:1_34:3) | 1.291 (0.680) | 1.444 (0.679) | -1.12 | Up | 0.229 |
| HexCer(d16:1/22:0) | 0.205 (0.102) | 0.227 (0.086) | -1.11 | Up | 0.22901 |
| Cer(d16:1/22:0) | 0.282 (0.110) | 0.337 (0.140) | -1.19 | Up | 0.22902 |
| PC ae C38:3 | 4.486 (1.440) | 4.944 (1.346) | -1.1 | Up | 0.22902 |
| TG(20:0_32:4) | 1.817 (1.089) | 2.183 (1.175) | -1.2 | Up | 0.23087 |
| PC ae C30:0 | 0.378 (0.147) | 0.419 (0.158) | -1.11 | Up | 0.23271 |
| TG(18:0_36:4) | 18.306 (15.486) | 19.469 (10.105) | -1.06 | Up | 0.23463 |
| TG(16:0_30:2) | 6.116 (5.780) | 6.880 (5.095) | -1.12 | Up | 0.23465 |
| PC ae C42:3 | 0.897 (0.358) | 0.989 (0.328) | -1.1 | Up | 0.23643 |
| TG(18:0_30:0) | 4.114 (3.657) | 5.262 (5.379) | -1.28 | Up | 0.23657 |
| C4:1 | 0.034 (0.015) | 0.039 (0.017) | -1.13 | Up | 0.23678 |
| TG(20:4_34:3) | 3.005 (2.016) | 3.259 (1.597) | -1.08 | Up | 0.23846 |
| C10:2 | 0.108 (0.084) | 0.126 (0.084) | -1.17 | Up | 0.23963 |
| PC ae C36:3 | 9.223 (3.187) | 9.959 (2.574) | -1.08 | Up | 0.24035 |
| lysoPC a C28:1 | 0.416 (0.163) | 0.482 (0.215) | -1.16 | Up | 0.2404 |
| SM C24:0 | 17.980 (6.088) | 19.970 (5.918) | -1.11 | Up | 0.24231 |
| TG(18:2_30:1) | 11.922 (8.281) | 14.106 (8.710) | -1.18 | Up | 0.24233 |
| TG(18:2_33:0) | 6.011 (3.315) | 6.882 (3.379) | -1.14 | Up | 0.24234 |
| SM (OH) C22:2 | 8.876 (2.995) | 9.610 (2.371) | -1.08 | Up | 0.24424 |
| CE(22:6) | 59.128 (31.397) | 66.889 (27.806) | -1.13 | Up | 0.24542 |
| TG(22:4_34:2) | 3.417 (2.050) | 3.828 (1.878) | -1.12 | Up | 0.24623 |
| DG(16:0_20:0) | 0.610 (0.280) | 0.654 (0.308) | -1.07 | Up | 0.24631 |
| TG(20:2_32:0) | 4.725 (2.905) | 5.349 (2.709) | -1.13 | Up | 0.25017 |
| TG(18:1_30:2) | 5.162 (3.581) | 6.290 (4.331) | -1.22 | Up | 0.25415 |
| TG(18:0_34:3) | 6.538 (3.845) | 7.434 (3.500) | -1.14 | Up | 0.25416 |
| HexCer(d18:1/20:0) | 0.373 (0.159) | 0.410 (0.128) | -1.1 | Up | 0.25458 |
| Hex2Cer(d18:1/22:0) | 0.176 (0.078) | 0.190 (0.056) | -1.08 | Up | 0.25613 |
| DG(18:2_20:4) | 0.383 (0.175) | 0.422 (0.169) | -1.1 | Up | 0.25815 |
| SM C16:1 | 18.521 (6.075) | 20.240 (4.833) | -1.09 | Up | 0.26014 |
| TG(17:2_34:3) | 0.684 (0.348) | 0.773 (0.366) | -1.13 | Up | 0.26022 |
| TG(14:0_35:2) | 1.347 (0.875) | 1.527 (0.908) | -1.13 | Up | 0.26426 |
| SM (OH) C24:1 | 0.924 (0.321) | 1.014 (0.300) | -1.1 | Up | 0.26427 |
| TG(20:2_36:5) | 0.213 (0.154) | 0.219 (0.098) | -1.03 | Up | 0.2643 |
| TG(18:0_32:2) | 3.756 (2.632) | 4.502 (2.944) | -1.2 | Up | 0.26431 |
| Val | 94.677 (48.018) | 83.790 (38.235) | 1.13 | Down | 0.26538 |
| CE(15:0) | 8.426 (4.036) | 9.457 (4.190) | -1.12 | Up | 0.26583 |
| TG(17:0_36:4) | 3.100 (1.961) | 3.412 (1.585) | -1.1 | Up | 0.26637 |
| Creatinine | 27.855 (11.946) | 30.942 (12.712) | -1.11 | Up | 0.2664 |
| DG(16:0_18:2) | 5.938 (2.895) | 6.963 (3.388) | -1.17 | Up | 0.26846 |
| TG(18:0_34:2) | 35.454 (21.202) | 40.530 (20.909) | -1.14 | Up | 0.26846 |
| GCDCA | 0.660 (0.735) | 0.686 (0.485) | -1.04 | Up | 0.27054 |
| TG(20:1_34:0) | 1.434 (0.751) | 1.626 (0.784) | -1.13 | Up | 0.27261 |
| HexCer(d18:1/18:1) | 0.068 (0.069) | 0.085 (0.066) | -1.25 | Up | 0.27327 |
| TG(18:2_32:0) | 169.937 (97.348) | 200.956 (109.580) | -1.18 | Up | 0.27473 |
| TG(16:0_40:8) | 2.983 (2.207) | 3.193 (1.636) | -1.07 | Up | 0.27474 |
| Hex2Cer(d18:1/20:0) | 0.072 (0.026) | 0.077 (0.019) | -1.06 | Up | 0.27671 |
| TG(18:1_31:0) | 5.633 (3.127) | 6.504 (3.520) | -1.15 | Up | 0.27686 |
| TG(14:0_34:3) | 6.800 (5.018) | 7.901 (5.563) | -1.16 | Up | 0.27687 |
| PC ae C30:2 | 0.102 (0.036) | 0.111 (0.029) | -1.09 | Up | 0.27893 |
| PC ae C34:2 | 16.507 (5.658) | 18.074 (5.067) | -1.09 | Up | 0.27897 |
| TG(20:4_34:2) | 23.263 (14.404) | 25.456 (11.665) | -1.09 | Up | 0.27897 |
| TG(17:0_34:2) | 6.465 (3.483) | 7.365 (3.449) | -1.14 | Up | 0.27899 |
| TG(22:4_32:0) | 1.793 (1.501) | 1.979 (1.302) | -1.1 | Up | 0.27899 |
| TG(14:0_34:0) | 7.915 (6.186) | 9.251 (6.696) | -1.17 | Up | 0.28112 |
| TG(17:2_36:3) | 0.562 (0.266) | 0.628 (0.276) | -1.12 | Up | 0.28114 |
| TG(18:2_34:2) | 380.498 (223.937) | 444.510 (239.322) | -1.17 | Up | 0.28545 |
| TG(16:0_32:0) | 112.463 (84.301) | 123.061 (66.598) | -1.09 | Up | 0.28762 |
| TG(14:0_36:3) | 41.257 (21.584) | 48.211 (23.739) | -1.17 | Up | 0.28763 |
| TG(22:6_34:2) | 13.583 (10.981) | 14.174 (7.961) | -1.04 | Up | 0.28763 |
| HexCer(d18:1/24:1) | 4.032 (1.671) | 4.400 (1.396) | -1.09 | Up | 0.2885 |
| Trp | 39.333 (14.059) | 38.574 (11.410) | 1.02 | Down | 0.28981 |
| TG(14:0_34:1) | 58.513 (45.516) | 64.982 (41.842) | -1.11 | Up | 0.28982 |
| TDCA | 0.225 (0.357) | 0.194 (0.264) | 1.16 | Down | 0.29186 |
| Cer(d18:2/18:0) | 0.069 (0.022) | 0.074 (0.020) | -1.08 | Up | 0.29399 |
| TG(16:0_28:1) | 4.931 (5.790) | 5.899 (7.091) | -1.2 | Up | 0.29867 |
| DG(18:2_20:0) | 0.080 (0.034) | 0.087 (0.028) | -1.09 | Up | 0.30076 |
| TG(14:0_34:2) | 37.518 (27.378) | 42.657 (26.855) | -1.14 | Up | 0.30091 |
| PC ae C34:3 | 10.134 (4.209) | 11.080 (3.925) | -1.09 | Up | 0.30158 |
| Hypoxanthine | 0.089 (0.036) | 0.159 (0.383) | -1.79 | Up | 0.30266 |
| TG(14:0_32:2) | 4.813 (4.555) | 5.559 (4.930) | -1.15 | Up | 0.30316 |
| TG(16:0_34:0) | 66.587 (40.363) | 75.734 (38.577) | -1.14 | Up | 0.30329 |
| CE(22:2) | 0.163 (0.077) | 0.178 (0.079) | -1.09 | Up | 0.30894 |
| PC aa C42:5 | 0.457 (0.175) | 0.495 (0.152) | -1.08 | Up | 0.31041 |
| TG(20:2_34:3) | 1.348 (0.714) | 1.505 (0.666) | -1.12 | Up | 0.31095 |
| Methanol | 325.125 (217.747) | 349.053 (496.200) | -1.07 | Up | 0.31232 |
| TG(16:0_36:4) | 248.513 (145.085) | 288.921 (151.742) | -1.16 | Up | 0.31692 |
| PC aa C38:3 | 54.514 (19.872) | 58.894 (17.396) | -1.08 | Up | 0.31693 |
| C6 (C4:1-DC) | 0.104 (0.085) | 0.125 (0.090) | -1.2 | Up | 0.31828 |
| PC ae C38:0 | 1.979 (0.786) | 2.153 (0.766) | -1.09 | Up | 0.31885 |
| Isobutyric acid | 4.910 (2.537) | 5.327 (2.292) | -1.09 | Up | 0.31913 |
| Met-SO | 0.288 (0.334) | 0.252 (0.506) | 1.14 | Down | 0.32105 |
| PC aa C32:0 | 22.061 (8.331) | 23.964 (6.649) | -1.09 | Up | 0.32157 |
| Acetic acid | 14.670 (6.580) | 20.802 (35.310) | -1.42 | Up | 0.32159 |
| TG(18:2_34:4) | 3.500 (2.400) | 3.731 (1.970) | -1.07 | Up | 0.32159 |
| TG(20:3_36:4) | 1.420 (0.756) | 1.512 (0.600) | -1.06 | Up | 0.32392 |
| Cer(d18:1/22:0) | 0.947 (0.323) | 1.038 (0.308) | -1.1 | Up | 0.32393 |
| TG(22:6_34:3) | 1.931 (1.557) | 2.030 (1.225) | -1.05 | Up | 0.32396 |
| TG(18:3_30:0) | 3.249 (3.132) | 3.834 (3.363) | -1.18 | Up | 0.32632 |
| Dimethyl sulfone | 17.453 (60.262) | 8.752 (32.335) | 1.99 | Down | 0.32856 |
| TG(18:0_36:3) | 40.944 (24.694) | 44.685 (21.069) | -1.09 | Up | 0.3287 |
| TG(18:1_30:0) | 67.103 (49.869) | 75.649 (48.791) | -1.13 | Up | 0.3287 |
| TG(14:0_36:1) | 8.392 (5.199) | 9.824 (6.137) | -1.17 | Up | 0.33109 |
| TG(20:4_32:2) | 1.982 (1.645) | 2.159 (1.369) | -1.09 | Up | 0.33347 |
| Ala | 403.870 (207.133) | 370.985 (147.556) | 1.09 | Down | 0.33348 |
| DG(14:1_18:1) | 0.125 (0.079) | 0.132 (0.058) | -1.06 | Up | 0.33554 |
| TG(18:2_34:3) | 45.872 (30.271) | 50.993 (27.652) | -1.11 | Up | 0.3359 |
| PC ae C34:0 | 1.704 (0.645) | 1.833 (0.553) | -1.08 | Up | 0.33746 |
| PC aa C36:3 | 192.190 (63.929) | 208.581 (56.403) | -1.09 | Up | 0.33827 |
| TG(16:0_38:6) | 8.554 (5.705) | 8.955 (4.059) | -1.05 | Up | 0.34074 |
| CE(15:1) | 0.509 (0.245) | 0.555 (0.220) | -1.09 | Up | 0.34551 |
| TG(16:0_38:3) | 17.157 (8.601) | 19.262 (9.115) | -1.12 | Up | 0.34564 |
| TG(20:1_34:1) | 10.102 (5.396) | 11.238 (5.748) | -1.11 | Up | 0.34565 |
| PC ae C40:5 | 3.423 (1.211) | 3.628 (0.948) | -1.06 | Up | 0.34809 |
| TG(16:0_37:3) | 2.227 (1.119) | 2.533 (1.243) | -1.14 | Up | 0.3481 |
| Cer(d16:1/24:0) | 0.155 (0.078) | 0.177 (0.082) | -1.14 | Up | 0.34811 |
| TG(16:1_30:1) | 3.011 (3.265) | 2.960 (2.523) | 1.02 | Down | 0.34811 |
| DG(16:0_16:1) | 1.110 (0.735) | 1.151 (0.580) | -1.04 | Up | 0.35058 |
| TG(20:4_32:0) | 10.771 (9.932) | 11.474 (7.662) | -1.07 | Up | 0.35058 |
| TG(18:2_33:2) | 5.416 (3.253) | 6.217 (3.332) | -1.15 | Up | 0.3506 |
| TG(20:3_32:0) | 4.895 (3.490) | 5.146 (2.807) | -1.05 | Up | 0.35308 |
| TG(18:2_36:1) | 50.671 (29.664) | 55.120 (25.770) | -1.09 | Up | 0.35309 |
| TG(22:5_32:0) | 3.999 (2.917) | 4.354 (2.465) | -1.09 | Up | 0.35309 |
| L-Lactic acid | 2276.850 (3101.896) | 1635.595 (848.017) | 1.39 | Down | 0.35507 |
| TG(18:3_32:0) | 12.814 (9.024) | 14.327 (8.440) | -1.12 | Up | 0.35553 |
| TG(18:0_32:1) | 10.665 (7.614) | 12.166 (8.272) | -1.14 | Up | 0.35558 |
| Citric acid | 59.212 (29.784) | 66.570 (34.952) | -1.12 | Up | 0.3556 |
| CE(18:0) | 17.749 (6.553) | 19.001 (5.474) | -1.07 | Up | 0.35662 |
| DG(18:2_18:2) | 10.689 (6.177) | 12.082 (6.566) | -1.13 | Up | 0.35807 |
| TG(16:0_33:2) | 6.768 (4.275) | 7.637 (4.243) | -1.13 | Up | 0.35809 |
| TG(18:1_32:0) | 310.768 (188.842) | 350.796 (197.639) | -1.13 | Up | 0.3581 |
| Cer(d18:1/20:0) | 0.290 (0.122) | 0.328 (0.129) | -1.13 | Up | 0.3606 |
| PC ae C32:2 | 0.821 (0.276) | 0.894 (0.207) | -1.09 | Up | 0.36061 |
| TG(16:0_32:1) | 127.844 (110.908) | 135.346 (91.878) | -1.06 | Up | 0.36062 |
| TG(18:1_30:1) | 23.919 (16.771) | 27.047 (18.631) | -1.13 | Up | 0.36062 |
| TG(17:0_34:1) | 8.067 (4.625) | 8.899 (4.315) | -1.1 | Up | 0.36063 |
| TG(18:2_38:6) | 3.992 (2.356) | 4.198 (1.828) | -1.05 | Up | 0.36317 |
| TMAO | 2.787 (2.034) | 2.597 (2.114) | 1.07 | Down | 0.36567 |
| TG(14:0_38:4) | 1.405 (0.885) | 1.535 (0.823) | -1.09 | Up | 0.36569 |
| TG(16:0_32:2) | 51.458 (39.895) | 56.132 (36.454) | -1.09 | Up | 0.3657 |
| TG(16:1_32:0) | 33.602 (34.208) | 33.380 (24.729) | 1.01 | Down | 0.3657 |
| TG(16:1_36:5) | 2.634 (1.728) | 2.830 (1.531) | -1.07 | Up | 0.36823 |
| TG(16:0_34:3) | 90.712 (53.617) | 103.120 (59.513) | -1.14 | Up | 0.36826 |
| TG(18:2_34:0) | 78.459 (40.824) | 89.775 (44.617) | -1.14 | Up | 0.36826 |
| DG(18:3_18:3) | 0.037 (0.048) | 0.027 (0.035) | 1.38 | Down | 0.36957 |
| TG(20:4_32:1) | 6.765 (6.944) | 6.847 (4.991) | -1.01 | Up | 0.37083 |
| Cer(d18:1/25:0) | 0.375 (0.150) | 0.402 (0.112) | -1.07 | Up | 0.3716 |
| HexCer(d18:2/22:0) | 0.598 (0.264) | 0.646 (0.208) | -1.08 | Up | 0.37171 |
| TG(20:0_32:3) | 1.650 (0.857) | 1.909 (1.014) | -1.16 | Up | 0.37598 |
| TG(18:0_36:1) | 7.190 (4.604) | 7.961 (4.519) | -1.11 | Up | 0.37599 |
| Cer(d18:0/26:1(OH)) | 0.170 (0.101) | 0.194 (0.087) | -1.14 | Up | 0.37751 |
| CE(17:0) | 5.682 (2.249) | 6.112 (2.110) | -1.08 | Up | 0.3799 |
| TG(18:1_33:3) | 0.897 (0.439) | 1.008 (0.490) | -1.12 | Up | 0.38119 |
| TG(16:1_28:0) | 2.046 (2.596) | 2.048 (2.330) | -1 | Up | 0.38367 |
| TG(18:3_34:2) | 32.141 (21.711) | 35.045 (18.471) | -1.09 | Up | 0.38381 |
| TG(17:1_34:3) | 0.966 (0.470) | 1.062 (0.513) | -1.1 | Up | 0.38564 |
| TG(17:1_36:4) | 1.548 (0.914) | 1.648 (0.794) | -1.06 | Up | 0.38643 |
| EPA | 0.142 (0.202) | 0.185 (0.268) | -1.3 | Up | 0.38799 |
| TG(18:0_30:1) | 1.904 (1.613) | 2.400 (2.562) | -1.26 | Up | 0.38909 |
| TG(18:1_33:0) | 9.613 (5.472) | 10.745 (5.608) | -1.12 | Up | 0.38909 |
| H1 | 4649.670 (1691.653) | 4723.360 (2082.725) | -1.02 | Up | 0.38911 |
| DG(18:1_18:3) | 1.144 (0.488) | 1.239 (0.497) | -1.08 | Up | 0.39018 |
| HexCer(d18:2/16:0) | 0.073 (0.031) | 0.082 (0.028) | -1.11 | Up | 0.3916 |
| Cer(d18:1/18:1) | 0.025 (0.012) | 0.026 (0.011) | -1.06 | Up | 0.39199 |
| TG(16:0_38:1) | 2.617 (1.344) | 2.864 (1.228) | -1.09 | Up | 0.39351 |
| TG(18:2_32:1) | 103.550 (53.540) | 120.311 (66.483) | -1.16 | Up | 0.39438 |
| PC aa C32:3 | 0.859 (0.304) | 0.964 (0.325) | -1.12 | Up | 0.39439 |
| DG(14:1_20:2) | 0.037 (0.038) | 0.045 (0.042) | -1.22 | Up | 0.39531 |
| PC aa C38:6 | 112.016 (50.191) | 121.223 (46.365) | -1.08 | Up | 0.39671 |
| TG(17:0_36:3) | 8.104 (4.054) | 8.964 (4.279) | -1.11 | Up | 0.39708 |
| DG(18:1_20:3) | 0.128 (0.103) | 0.143 (0.116) | -1.12 | Up | 0.39852 |
| TG(20:1_32:1) | 2.596 (1.490) | 2.871 (1.411) | -1.11 | Up | 0.39973 |
| TG(14:0_36:2) | 40.390 (22.089) | 45.632 (22.503) | -1.13 | Up | 0.39978 |
| TG(16:0_33:1) | 14.155 (9.539) | 15.605 (9.113) | -1.1 | Up | 0.39979 |
| TG(18:2_38:4) | 5.547 (2.898) | 6.077 (2.644) | -1.1 | Up | 0.40247 |
| Creatine | 23.855 (12.739) | 22.040 (11.949) | 1.08 | Down | 0.40517 |
| TG(16:0_34:1) | 508.144 (306.170) | 569.938 (308.271) | -1.12 | Up | 0.4052 |
| Cer(d18:0/26:1) | 0.010 (0.010) | 0.012 (0.011) | -1.19 | Up | 0.40617 |
| TG(22:5_34:2) | 8.182 (4.491) | 8.974 (4.011) | -1.1 | Up | 0.40786 |
| TG(20:2_34:1) | 10.123 (4.973) | 11.042 (4.944) | -1.09 | Up | 0.41004 |
| PC ae C42:2 | 0.473 (0.187) | 0.499 (0.161) | -1.06 | Up | 0.41064 |
| Pyruvic acid | 99.255 (76.025) | 82.895 (65.709) | 1.2 | Down | 0.41066 |
| Hex2Cer(d18:1/26:1) | 0.040 (0.022) | 0.044 (0.021) | -1.11 | Up | 0.41221 |
| C3-OH | 0.034 (0.022) | 0.040 (0.025) | -1.18 | Up | 0.41338 |
| lysoPC a C26:0 | 0.186 (0.093) | 0.204 (0.102) | -1.1 | Up | 0.41338 |
| C18 | 0.031 (0.022) | 0.026 (0.022) | 1.17 | Down | 0.41372 |
| CE(18:1) | 491.980 (203.674) | 526.740 (173.676) | -1.07 | Up | 0.41397 |
| C16 | 0.089 (0.035) | 0.092 (0.031) | -1.03 | Up | 0.41603 |
| Hypoxanthine.1 | 6.519 (4.047) | 5.861 (3.741) | 1.11 | Down | 0.4189 |
| TG(20:4_36:4) | 4.513 (2.881) | 4.796 (2.346) | -1.06 | Up | 0.4189 |
| CE(22:0) | 0.401 (0.314) | 0.462 (0.294) | -1.15 | Up | 0.42031 |
| TG(16:0_38:2) | 11.334 (5.981) | 12.434 (5.960) | -1.1 | Up | 0.42163 |
| TG(17:1_36:3) | 3.482 (1.687) | 3.841 (1.871) | -1.1 | Up | 0.42167 |
| TG(18:3_34:0) | 6.716 (3.893) | 7.362 (3.552) | -1.1 | Up | 0.42168 |
| GLCA | 0.006 (0.008) | 0.005 (0.005) | 1.02 | Down | 0.42173 |
| TG(16:0_38:4) | 18.127 (9.159) | 19.721 (8.572) | -1.09 | Up | 0.42406 |
| TG(18:3_34:3) | 4.334 (3.456) | 4.450 (2.719) | -1.03 | Up | 0.42446 |
| t4-OH-Pro | 11.397 (5.205) | 10.602 (3.508) | 1.07 | Down | 0.42604 |
| DG(16:1_20:0) | 0.223 (0.472) | 0.175 (0.154) | 1.28 | Down | 0.42668 |
| Acetone | 17.363 (21.698) | 22.745 (36.466) | -1.31 | Up | 0.42723 |
| TG(18:0_38:6) | 1.620 (0.930) | 1.690 (0.685) | -1.04 | Up | 0.42724 |
| Serotonin | 0.521 (0.372) | 0.458 (0.333) | 1.14 | Down | 0.42893 |
| Kynurenine | 1.484 (0.561) | 1.388 (0.354) | 1.07 | Down | 0.43003 |
| CE(14:0) | 25.084 (13.228) | 27.862 (14.209) | -1.11 | Up | 0.43005 |
| TG(16:0_34:2) | 405.767 (228.496) | 460.175 (244.410) | -1.13 | Up | 0.43006 |
| DG-O(16:0_18:1) | 0.095 (0.060) | 0.107 (0.065) | -1.13 | Up | 0.43086 |
| lysoPC a C16:0 | 43.059 (24.195) | 39.513 (16.204) | 1.09 | Down | 0.43288 |
| TG(16:0_32:3) | 6.652 (5.401) | 7.522 (5.542) | -1.13 | Up | 0.43288 |
| TG(22:6_34:1) | 17.840 (17.651) | 17.103 (10.618) | 1.04 | Down | 0.4357 |
| TG(14:0_38:5) | 1.080 (0.834) | 1.172 (0.758) | -1.09 | Up | 0.43571 |
| TG(16:1_36:4) | 19.749 (11.244) | 22.012 (11.756) | -1.11 | Up | 0.43571 |
| TG(18:3_33:2) | 0.612 (0.464) | 0.670 (0.434) | -1.09 | Up | 0.43572 |
| Hex2Cer(d18:1/24:0) | 0.128 (0.048) | 0.136 (0.041) | -1.06 | Up | 0.43632 |
| TG(20:0_34:1) | 1.996 (0.806) | 2.133 (0.755) | -1.07 | Up | 0.43649 |
| Carnitine | 12.261 (16.658) | 10.650 (12.591) | 1.15 | Down | 0.43846 |
| TG(16:0_35:1) | 11.010 (6.539) | 12.120 (6.231) | -1.1 | Up | 0.43946 |
| TG(22:5_34:3) | 1.291 (0.741) | 1.413 (0.655) | -1.09 | Up | 0.44001 |
| DG(16:1_18:2) | 1.349 (0.734) | 1.531 (0.885) | -1.14 | Up | 0.44138 |
| TG(18:2_35:2) | 7.490 (4.267) | 8.167 (3.956) | -1.09 | Up | 0.4414 |
| CE(17:1) | 7.567 (3.130) | 8.082 (2.830) | -1.07 | Up | 0.44258 |
| SM C18:1 | 12.945 (4.754) | 13.738 (3.297) | -1.06 | Up | 0.44417 |
| TG(18:2_33:1) | 12.485 (6.228) | 14.290 (7.421) | -1.14 | Up | 0.4442 |
| Lys | 164.540 (59.458) | 162.013 (56.717) | 1.02 | Down | 0.44421 |
| TG(18:1_34:3) | 88.857 (44.829) | 100.071 (51.926) | -1.13 | Up | 0.44425 |
| TG(16:1_32:1) | 28.739 (29.147) | 27.872 (21.023) | 1.03 | Down | 0.44426 |
| TG(20:4_30:0) | 2.587 (3.304) | 2.721 (2.400) | -1.05 | Up | 0.44426 |
| C8 | 0.200 (0.171) | 0.234 (0.193) | -1.17 | Up | 0.44443 |
| PC ae C42:4 | 1.035 (0.407) | 1.103 (0.382) | -1.07 | Up | 0.44512 |
| PC aa C38:1 | 0.755 (0.661) | 0.819 (0.600) | -1.09 | Up | 0.44617 |
| TG(16:0_35:3) | 6.314 (3.221) | 7.053 (3.523) | -1.12 | Up | 0.44713 |
| TG(17:1_34:2) | 6.388 (3.152) | 7.187 (3.637) | -1.13 | Up | 0.44713 |
| TG(18:1_32:2) | 53.219 (26.077) | 61.749 (30.838) | -1.16 | Up | 0.44713 |
| DG(18:1_18:2) | 17.124 (8.278) | 19.619 (11.107) | -1.15 | Up | 0.44999 |
| TG(16:1_34:0) | 15.268 (10.354) | 16.699 (9.836) | -1.09 | Up | 0.44999 |
| Lac | 4586.130 (6393.653) | 3523.715 (1675.457) | 1.3 | Down | 0.45002 |
| TG(17:0_34:3) | 1.302 (0.686) | 1.419 (0.700) | -1.09 | Up | 0.45074 |
| Pro | 133.436 (47.550) | 147.703 (55.029) | -1.11 | Up | 0.45279 |
| C2 | 2.649 (1.621) | 2.966 (1.818) | -1.12 | Up | 0.4529 |
| TG(17:0_32:1) | 2.261 (1.693) | 2.514 (1.725) | -1.11 | Up | 0.45581 |
| TG(18:2_38:5) | 5.769 (2.916) | 6.228 (2.580) | -1.08 | Up | 0.45793 |
| TG(16:0_34:4) | 7.283 (5.229) | 7.825 (4.668) | -1.07 | Up | 0.45871 |
| TG(16:1_32:2) | 6.899 (5.202) | 7.437 (5.045) | -1.08 | Up | 0.45871 |
| TG(20:3_32:2) | 0.975 (0.594) | 1.027 (0.531) | -1.05 | Up | 0.45871 |
| TG(22:6_32:0) | 7.042 (7.677) | 6.462 (3.975) | 1.09 | Down | 0.45871 |
| CE(22:5) | 4.235 (2.081) | 4.605 (1.897) | -1.09 | Up | 0.46163 |
| TG(18:1_36:0) | 8.198 (4.266) | 8.986 (4.079) | -1.1 | Up | 0.46163 |
| TG(18:3_34:1) | 50.618 (26.619) | 55.535 (26.403) | -1.1 | Up | 0.46163 |
| TG(18:2_34:1) | 585.285 (284.530) | 667.830 (336.189) | -1.14 | Up | 0.46164 |
| 3-Hydroxybutyric acid | 117.022 (159.912) | 207.050 (461.829) | -1.77 | Up | 0.46165 |
| CE(14:1) | 1.111 (0.764) | 1.275 (0.913) | -1.15 | Up | 0.46456 |
| TG(18:1_32:3) | 5.395 (2.975) | 6.030 (3.280) | -1.12 | Up | 0.46457 |
| PC ae C42:5 | 2.062 (0.782) | 2.160 (0.607) | -1.05 | Up | 0.46747 |
| HexCer(d18:2/18:0) | 0.039 (0.016) | 0.041 (0.014) | -1.06 | Up | 0.47004 |
| lysoPC a C18:1 | 6.120 (3.686) | 5.562 (2.743) | 1.1 | Down | 0.47341 |
| TG(20:1_32:2) | 1.184 (0.587) | 1.314 (0.588) | -1.11 | Up | 0.47341 |
| TG(16:1_38:3) | 1.751 (0.825) | 1.959 (0.925) | -1.12 | Up | 0.47935 |
| PC ae C40:4 | 2.692 (0.929) | 2.851 (0.791) | -1.06 | Up | 0.47936 |
| TG(20:3_36:5) | 0.323 (0.190) | 0.329 (0.141) | -1.02 | Up | 0.47937 |
| TG(22:6_32:1) | 4.252 (4.741) | 3.950 (2.887) | 1.08 | Down | 0.47939 |
| DHEAS | 1.484 (1.189) | 1.374 (1.245) | 1.08 | Down | 0.48237 |
| TG(18:2_36:4) | 53.705 (59.907) | 51.838 (31.826) | 1.04 | Down | 0.48239 |
| TG(20:3_32:1) | 3.315 (2.338) | 3.424 (1.960) | -1.03 | Up | 0.48239 |
| SDMA | 0.552 (0.207) | 0.585 (0.209) | -1.06 | Up | 0.48472 |
| DHA | 3.071 (2.332) | 3.894 (3.992) | -1.27 | Up | 0.48537 |
| TG(16:0_36:3) | 654.015 (320.314) | 744.945 (375.227) | -1.14 | Up | 0.48539 |
| HexCer(d18:2/23:0) | 0.280 (0.116) | 0.298 (0.112) | -1.06 | Up | 0.48544 |
| Cer(d18:1/23:0) | 0.992 (0.370) | 1.054 (0.300) | -1.06 | Up | 0.48837 |
| CDCA | 0.238 (0.234) | 0.187 (0.172) | 1.27 | Down | 0.49199 |
| TG(18:1_33:2) | 9.623 (4.819) | 10.864 (5.612) | -1.13 | Up | 0.49444 |
| TG(18:2_36:3) | 121.073 (97.658) | 124.895 (67.410) | -1.03 | Up | 0.49446 |
| TG(18:2_36:5) | 10.707 (14.796) | 9.480 (6.799) | 1.13 | Down | 0.49446 |
| TG(20:4_36:5) | 1.014 (0.841) | 1.012 (0.631) | 1 | Down | 0.49447 |
| PC aa C40:2 | 0.267 (0.094) | 0.280 (0.080) | -1.05 | Up | 0.49454 |
| TG(18:3_32:1) | 8.919 (6.251) | 9.849 (6.248) | -1.1 | Up | 0.4975 |
| Hex2Cer(d18:1/24:1) | 0.301 (0.110) | 0.317 (0.102) | -1.05 | Up | 0.49923 |
| TG(17:1_38:6) | 0.164 (0.073) | 0.175 (0.068) | -1.07 | Up | 0.49994 |
| DG(18:2_18:3) | 0.794 (0.552) | 0.802 (0.393) | -1.01 | Up | 0.50054 |
| PC aa C42:2 | 0.194 (0.068) | 0.204 (0.056) | -1.05 | Up | 0.50098 |
| TG(20:3_34:2) | 9.539 (4.667) | 10.362 (4.491) | -1.09 | Up | 0.50669 |
| GUDCA | 0.059 (0.098) | 0.041 (0.040) | 1.43 | Down | 0.50953 |
| TG(17:1_32:1) | 2.180 (1.565) | 2.318 (1.419) | -1.06 | Up | 0.50978 |
| lysoPC a C20:4 | 2.191 (1.641) | 1.932 (1.040) | 1.13 | Down | 0.51284 |
| TG(18:0_36:2) | 30.506 (16.543) | 32.699 (14.321) | -1.07 | Up | 0.51287 |
| Hex2Cer(d18:1/16:0) | 3.261 (1.147) | 3.416 (0.965) | -1.05 | Up | 0.51573 |
| AABA | 11.563 (7.016) | 11.387 (7.380) | 1.02 | Down | 0.51598 |
| DG(18:1_22:5) | 0.040 (0.024) | 0.044 (0.021) | -1.08 | Up | 0.51753 |
| CE(16:1) | 97.307 (60.396) | 103.909 (53.532) | -1.07 | Up | 0.51906 |
| Cer(d18:2/23:0) | 0.116 (0.051) | 0.122 (0.036) | -1.05 | Up | 0.51954 |
| TG(18:0_38:7) | 0.309 (0.187) | 0.333 (0.142) | -1.08 | Up | 0.52725 |
| PC aa C36:4 | 272.081 (93.543) | 292.058 (82.023) | -1.07 | Up | 0.52846 |
| TG(20:5_34:2) | 1.862 (1.510) | 1.892 (1.203) | -1.02 | Up | 0.52849 |
| PC aa C34:3 | 26.636 (10.201) | 29.758 (12.181) | -1.12 | Up | 0.53163 |
| TG(22:5_32:1) | 2.512 (1.979) | 2.568 (1.521) | -1.02 | Up | 0.5348 |
| TG(14:0_35:1) | 1.385 (1.072) | 1.545 (1.321) | -1.12 | Up | 0.53481 |
| TG(16:1_34:2) | 75.213 (43.464) | 84.570 (51.432) | -1.12 | Up | 0.53481 |
| TG(18:2_35:1) | 9.319 (4.697) | 10.197 (4.821) | -1.09 | Up | 0.53481 |
| TG(20:4_36:2) | 12.920 (5.836) | 13.724 (5.757) | -1.06 | Up | 0.53718 |
| DG(18:1_20:4) | 0.438 (0.185) | 0.451 (0.194) | -1.03 | Up | 0.5377 |
| PC aa C40:6 | 27.002 (13.400) | 28.749 (11.925) | -1.06 | Up | 0.53979 |
| TG(20:1_30:1) | 0.230 (0.138) | 0.242 (0.122) | -1.05 | Up | 0.54096 |
| TG(18:2_35:3) | 2.271 (1.397) | 2.429 (1.198) | -1.07 | Up | 0.54115 |
| C14 | 0.089 (0.071) | 0.099 (0.066) | -1.11 | Up | 0.54142 |
| TG(16:0_38:5) | 15.778 (8.602) | 16.880 (7.673) | -1.07 | Up | 0.54727 |
| TG(18:1_34:2) | 659.230 (316.974) | 749.890 (376.884) | -1.14 | Up | 0.54755 |
| Ind-SO4 | 3.077 (2.428) | 3.359 (2.590) | -1.09 | Up | 0.55076 |
| TG(16:1_34:3) | 11.749 (7.133) | 13.072 (8.295) | -1.11 | Up | 0.55397 |
| TG(16:1_36:3) | 55.100 (28.185) | 61.798 (35.640) | -1.12 | Up | 0.55398 |
| PC aa C42:1 | 0.343 (0.152) | 0.361 (0.126) | -1.05 | Up | 0.55898 |
| TG(16:0_36:6) | 1.919 (1.661) | 1.928 (1.237) | -1 | Up | 0.56043 |
| lysoPC a C18:0 | 9.752 (6.452) | 8.905 (3.593) | 1.1 | Down | 0.56044 |
| TG(18:3_36:4) | 7.801 (10.705) | 6.816 (4.871) | 1.14 | Down | 0.56044 |
| TG(16:0_35:2) | 14.653 (7.813) | 15.895 (7.833) | -1.08 | Up | 0.56691 |
| TG(16:0_40:7) | 6.079 (4.381) | 6.026 (2.964) | 1.01 | Down | 0.56693 |
| TG(16:1_38:5) | 1.904 (1.140) | 1.962 (0.950) | -1.03 | Up | 0.56693 |
| DG(18:0_20:4) | 0.047 (0.033) | 0.051 (0.033) | -1.09 | Up | 0.56881 |
| PC ae C38:6 | 7.282 (2.871) | 7.615 (2.305) | -1.05 | Up | 0.56946 |
| TG(18:1_33:1) | 22.715 (12.251) | 24.733 (13.669) | -1.09 | Up | 0.5702 |
| TG(18:1_38:6) | 6.094 (3.639) | 6.159 (2.633) | -1.01 | Up | 0.5702 |
| PC aa C36:1 | 53.802 (21.940) | 56.349 (17.987) | -1.05 | Up | 0.57188 |
| TG(20:4_36:3) | 10.311 (5.216) | 10.929 (4.532) | -1.06 | Up | 0.57311 |
| TG(18:3_38:5) | 0.687 (0.420) | 0.721 (0.367) | -1.05 | Up | 0.57675 |
| CA | 0.069 (0.140) | 0.096 (0.184) | -1.4 | Up | 0.57834 |
| DG(18:1_18:4) | 0.056 (0.050) | 0.063 (0.053) | -1.12 | Up | 0.57941 |
| TG(20:2_32:1) | 3.253 (1.737) | 3.527 (1.671) | -1.08 | Up | 0.58003 |
| D-Glucose | 2649.980 (1005.295) | 2919.765 (1665.056) | -1.1 | Up | 0.58199 |
| CE(22:1) | 0.538 (0.327) | 0.580 (0.284) | -1.08 | Up | 0.58255 |
| C18:2 | 0.041 (0.020) | 0.043 (0.017) | -1.03 | Up | 0.58313 |
| Cit | 17.800 (7.802) | 16.969 (5.474) | 1.05 | Down | 0.58316 |
| DG(16:1_18:0) | 0.239 (0.121) | 0.254 (0.108) | -1.06 | Up | 0.58638 |
| TG(20:4_33:2) | 0.365 (0.227) | 0.381 (0.202) | -1.04 | Up | 0.58665 |
| TG(20:3_36:3) | 3.266 (1.485) | 3.433 (1.256) | -1.05 | Up | 0.58952 |
| 3-Met-His | 5.157 (6.227) | 5.998 (6.337) | -1.16 | Up | 0.58995 |
| PC ae C36:1 | 8.692 (3.161) | 9.057 (2.448) | -1.04 | Up | 0.58995 |
| DG(16:0_18:1) | 8.020 (3.851) | 8.843 (4.357) | -1.1 | Up | 0.58996 |
| TG(20:4_34:1) | 30.713 (17.134) | 32.568 (15.471) | -1.06 | Up | 0.58996 |
| PC aa C36:6 | 1.031 (0.589) | 1.122 (0.631) | -1.09 | Up | 0.59328 |
| CE(18:3) | 76.145 (33.321) | 85.802 (43.156) | -1.13 | Up | 0.59329 |
| TG(18:1_32:1) | 205.231 (117.947) | 226.667 (131.938) | -1.1 | Up | 0.59661 |
| lysoPC a C24:0 | 0.118 (0.049) | 0.123 (0.045) | -1.04 | Up | 0.59992 |
| PC ae C32:1 | 3.578 (1.231) | 3.862 (1.009) | -1.08 | Up | 0.59995 |
| TG(16:0_38:7) | 1.629 (1.317) | 1.670 (0.984) | -1.02 | Up | 0.59995 |
| TG(18:2_36:2) | 136.142 (83.051) | 144.554 (72.463) | -1.06 | Up | 0.59995 |
| HCys | 4.672 (2.967) | 4.761 (1.939) | -1.02 | Up | 0.59996 |
| lysoPC a C17:0 | 0.583 (0.319) | 0.535 (0.210) | 1.09 | Down | 0.59997 |
| C16:1-OH | 0.012 (0.011) | 0.014 (0.012) | -1.14 | Up | 0.60108 |
| Cer(d18:2/24:0) | 0.363 (0.153) | 0.386 (0.133) | -1.07 | Up | 0.60331 |
| Cer(d18:2/18:1) | 0.004 (0.002) | 0.004 (0.002) | -1.02 | Up | 0.60346 |
| TG(20:4_34:0) | 4.480 (2.515) | 4.757 (2.221) | -1.06 | Up | 0.60406 |
| Met | 22.346 (10.177) | 23.009 (8.184) | -1.03 | Up | 0.61 |
| Propylene glycol | 0.248 (0.260) | 0.324 (0.425) | -1.3 | Up | 0.6127 |
| GLCAS | 0.107 (0.145) | 0.109 (0.100) | -1.02 | Up | 0.6134 |
| TG(16:1_33:1) | 3.079 (2.062) | 3.266 (1.998) | -1.06 | Up | 0.61679 |
| TG(20:3_34:1) | 12.903 (6.277) | 13.586 (5.899) | -1.05 | Up | 0.61779 |
| C3 | 0.236 (0.081) | 0.235 (0.082) | 1 | Down | 0.62016 |
| TG(17:2_36:4) | 1.118 (0.564) | 1.194 (0.542) | -1.07 | Up | 0.62016 |
| TG(18:1_35:3) | 3.767 (1.879) | 4.057 (1.948) | -1.08 | Up | 0.62018 |
| TG(17:1_34:1) | 9.584 (5.248) | 10.338 (5.371) | -1.08 | Up | 0.62699 |
| PC aa C42:0 | 0.673 (0.295) | 0.702 (0.233) | -1.04 | Up | 0.6289 |
| TG(20:3_34:0) | 1.697 (0.838) | 1.783 (0.757) | -1.05 | Up | 0.63234 |
| TG(20:3_34:3) | 1.493 (0.777) | 1.573 (0.716) | -1.05 | Up | 0.63286 |
| TG(22:4_32:2) | 0.125 (0.081) | 0.135 (0.079) | -1.08 | Up | 0.63382 |
| TG(18:3_38:6) | 0.455 (0.322) | 0.454 (0.250) | 1 | Down | 0.63383 |
| Cer(d18:0/18:0) | 0.021 (0.019) | 0.023 (0.020) | -1.09 | Up | 0.6341 |
| lysoPC a C16:1 | 0.876 (0.522) | 0.835 (0.479) | 1.05 | Down | 0.63726 |
| Cer(d18:1/26:1) | 0.024 (0.009) | 0.025 (0.008) | -1.04 | Up | 0.63879 |
| Succinate | 12.965 (4.736) | 13.002 (5.655) | -1 | Up | 0.64068 |
| ADMA | 0.371 (0.119) | 0.369 (0.091) | 1.01 | Down | 0.64069 |
| Cer(d18:0/20:0) | 0.063 (0.035) | 0.068 (0.034) | -1.07 | Up | 0.64302 |
| TG(16:1_34:1) | 120.669 (77.725) | 131.049 (82.292) | -1.09 | Up | 0.64414 |
| Cer(d18:2/20:0) | 0.073 (0.027) | 0.076 (0.024) | -1.05 | Up | 0.64748 |
| Thr | 191.042 (84.983) | 196.381 (71.384) | -1.03 | Up | 0.64759 |
| TG(20:5_36:3) | 1.008 (0.645) | 1.042 (0.598) | -1.03 | Up | 0.6476 |
| TG(17:2_34:2) | 0.695 (0.330) | 0.753 (0.354) | -1.08 | Up | 0.65107 |
| TG(17:2_38:5) | 0.268 (0.119) | 0.284 (0.120) | -1.06 | Up | 0.65453 |
| TG(16:1_38:4) | 2.229 (1.118) | 2.340 (1.093) | -1.05 | Up | 0.65556 |
| TG(18:3_36:1) | 5.219 (3.811) | 5.190 (2.473) | 1.01 | Down | 0.65801 |
| PC aa C32:1 | 29.262 (21.864) | 29.328 (17.883) | -1 | Up | 0.6615 |
| TG(18:1_34:4) | 6.853 (3.586) | 7.319 (3.588) | -1.07 | Up | 0.6615 |
| DG(18:1_20:2) | 0.079 (0.058) | 0.087 (0.062) | -1.09 | Up | 0.66352 |
| TG(18:3_36:3) | 16.561 (15.896) | 15.619 (8.826) | 1.06 | Down | 0.66497 |
| Isopropyl alcohol | 12.018 (10.140) | 13.207 (11.241) | -1.1 | Up | 0.66498 |
| PC ae C40:6 | 4.888 (2.025) | 5.057 (1.484) | -1.03 | Up | 0.67026 |
| PC aa C38:0 | 2.917 (1.313) | 3.031 (1.059) | -1.04 | Up | 0.67035 |
| C16:1 | 0.018 (0.019) | 0.020 (0.019) | -1.09 | Up | 0.6814 |
| Gly | 172.761 (82.034) | 158.984 (49.644) | 1.09 | Down | 0.686 |
| TG(18:1_38:7) | 1.111 (0.575) | 1.160 (0.519) | -1.04 | Up | 0.68872 |
| Cer(d18:2/16:0) | 0.108 (0.038) | 0.115 (0.031) | -1.07 | Up | 0.68953 |
| TG(16:0_36:2) | 592.565 (298.389) | 643.370 (329.550) | -1.09 | Up | 0.68962 |
| Gln | 433.315 (144.416) | 441.195 (101.771) | -1.02 | Up | 0.68963 |
| PC aa C36:5 | 15.601 (7.722) | 16.468 (9.635) | -1.06 | Up | 0.69317 |
| PC ae C44:3 | 0.151 (0.060) | 0.156 (0.053) | -1.03 | Up | 0.69396 |
| PC aa C40:1 | 0.228 (0.198) | 0.233 (0.201) | -1.02 | Up | 0.69438 |
| TG(17:1_36:5) | 0.300 (0.169) | 0.307 (0.154) | -1.02 | Up | 0.69673 |
| AA | 3.612 (2.451) | 3.780 (2.408) | -1.05 | Up | 0.69916 |
| Histamine | 0.010 (0.011) | 0.011 (0.013) | -1.14 | Up | 0.70236 |
| TG(18:1_36:3) | 229.242 (128.463) | 241.833 (121.916) | -1.05 | Up | 0.70386 |
| C16:2 | 0.022 (0.018) | 0.024 (0.017) | -1.06 | Up | 0.704 |
| Choline.1 | 7.983 (2.941) | 8.225 (2.875) | -1.03 | Up | 0.71056 |
| GDCA | 0.530 (0.663) | 0.492 (0.494) | 1.08 | Down | 0.71098 |
| Cer(d18:1/24:0) | 2.343 (0.909) | 2.507 (0.877) | -1.07 | Up | 0.71101 |
| 1-Methylhistidine | 97.597 (39.668) | 97.062 (47.796) | 1.01 | Down | 0.71103 |
| TG(18:1_34:1) | 932.115 (461.629) | 1004.820 (482.780) | -1.08 | Up | 0.71461 |
| DG(18:1_22:6) | 0.316 (0.190) | 0.330 (0.157) | -1.05 | Up | 0.71786 |
| DiCA(12:0) | 0.151 (0.154) | 0.139 (0.138) | 1.09 | Down | 0.72148 |
| TG(18:1_38:5) | 9.198 (3.949) | 9.513 (3.938) | -1.03 | Up | 0.72159 |
| PC ae C36:5 | 11.563 (4.691) | 12.115 (3.760) | -1.05 | Up | 0.72178 |
| Cortisone | 0.099 (0.059) | 0.106 (0.070) | -1.06 | Up | 0.73425 |
| TG(18:1_36:5) | 16.913 (15.384) | 15.945 (8.499) | 1.06 | Down | 0.73988 |
| TG(17:2_38:6) | 0.236 (0.122) | 0.245 (0.101) | -1.04 | Up | 0.74156 |
| Formate | 19.070 (8.756) | 44.885 (88.374) | -2.35 | Up | 0.74717 |
| TG(18:1_36:4) | 92.085 (66.361) | 93.470 (46.113) | -1.02 | Up | 0.74717 |
| DG(18:2_18:4) | 0.070 (0.044) | 0.067 (0.043) | 1.04 | Down | 0.75234 |
| Betaine | 9.421 (6.174) | 10.976 (13.749) | -1.17 | Up | 0.75446 |
| Trigonelline | 0.418 (0.458) | 0.501 (0.890) | -1.2 | Up | 0.7618 |
| Arg | 71.558 (24.143) | 71.046 (19.335) | 1.01 | Down | 0.7618 |
| CE(20:5) | 29.056 (21.197) | 30.136 (19.750) | -1.04 | Up | 0.76546 |
| 3-IAA | 1.261 (0.851) | 1.378 (1.051) | -1.09 | Up | 0.76546 |
| Cer(d18:0/18:0(OH)) | 0.306 (0.260) | 0.317 (0.230) | -1.04 | Up | 0.76678 |
| FA(20:3) | 0.807 (1.086) | 0.844 (1.007) | -1.05 | Up | 0.76695 |
| TG(18:1_36:1) | 71.867 (36.106) | 75.912 (33.658) | -1.06 | Up | 0.76914 |
| Cer(d18:1/26:0) | 0.051 (0.020) | 0.052 (0.017) | -1.02 | Up | 0.7711 |
| PC aa C30:2 | 0.140 (0.086) | 0.152 (0.098) | -1.08 | Up | 0.77649 |
| TG(22:5_34:1) | 9.739 (5.232) | 10.058 (4.788) | -1.03 | Up | 0.77694 |
| Xanthine | 0.973 (0.787) | 0.902 (0.689) | 1.08 | Down | 0.77984 |
| TrpBetaine | 0.448 (0.519) | 0.462 (0.649) | -1.03 | Up | 0.78019 |
| PC aa C42:6 | 0.471 (0.183) | 0.488 (0.150) | -1.04 | Up | 0.78019 |
| TLCA | 0.002 (0.004) | 0.002 (0.005) | 1.11 | Down | 0.78401 |
| C3:1 | 0.021 (0.012) | 0.022 (0.013) | -1.04 | Up | 0.78627 |
| 3-IPA | 0.571 (1.085) | 0.451 (0.534) | 1.27 | Down | 0.7911 |
| Ethanol | 191.440 (132.882) | 190.643 (118.897) | 1 | Down | 0.7913 |
| Sarcosine | 0.522 (0.461) | 0.653 (0.820) | -1.25 | Up | 0.79404 |
| TG(20:5_34:0) | 0.467 (0.338) | 0.492 (0.342) | -1.05 | Up | 0.795 |
| Cer(d18:2/22:0) | 0.231 (0.086) | 0.235 (0.071) | -1.02 | Up | 0.79871 |
| C16-OH | 0.020 (0.019) | 0.019 (0.018) | 1.06 | Down | 0.80158 |
| HexCer(d18:1/14:0) | 0.028 (0.013) | 0.029 (0.011) | -1.04 | Up | 0.80228 |
| Cer(d18:2/24:1) | 0.175 (0.065) | 0.186 (0.051) | -1.06 | Up | 0.80613 |
| PC ae C34:1 | 13.698 (4.947) | 14.324 (3.930) | -1.05 | Up | 0.80613 |
| TG(22:1_32:5) | 0.079 (0.059) | 0.070 (0.037) | 1.12 | Down | 0.80613 |
| Ser | 84.681 (34.881) | 86.255 (27.086) | -1.02 | Up | 0.80987 |
| C3-DC (C4-OH) | 0.093 (0.037) | 0.094 (0.033) | -1.02 | Up | 0.81269 |
| TG(16:0_40:6) | 5.671 (3.159) | 5.823 (2.673) | -1.03 | Up | 0.8159 |
| Betaine.1 | 15.827 (5.487) | 15.601 (4.098) | 1.01 | Down | 0.8173 |
| PC ae C38:4 | 15.329 (5.367) | 15.895 (4.207) | -1.04 | Up | 0.81734 |
| His | 95.382 (45.270) | 94.469 (36.545) | 1.01 | Down | 0.82108 |
| TG(18:1_35:2) | 13.458 (6.472) | 14.286 (6.944) | -1.06 | Up | 0.82108 |
| beta-Ala | 2.631 (3.346) | 2.583 (3.061) | 1.02 | Down | 0.82109 |
| TG(20:5_34:1) | 2.448 (1.949) | 2.433 (1.727) | 1.01 | Down | 0.82109 |
| C14:1-OH | 0.021 (0.014) | 0.022 (0.015) | -1.04 | Up | 0.82173 |
| PC ae C44:6 | 1.486 (0.598) | 1.513 (0.479) | -1.02 | Up | 0.82332 |
| PC aa C38:4 | 110.013 (39.328) | 116.216 (34.635) | -1.06 | Up | 0.82481 |
| PC aa C34:1 | 305.933 (114.078) | 316.447 (84.658) | -1.03 | Up | 0.82483 |
| TG(20:5_36:2) | 1.106 (0.696) | 1.128 (0.696) | -1.02 | Up | 0.82483 |
| PC aa C38:5 | 51.166 (18.299) | 53.059 (18.088) | -1.04 | Up | 0.82484 |
| Cer(d18:1/18:0(OH)) | 0.090 (0.056) | 0.083 (0.060) | 1.08 | Down | 0.82942 |
| PC ae C38:5 | 17.735 (6.144) | 18.297 (4.490) | -1.03 | Up | 0.83231 |
| DG(18:1_18:1) | 6.769 (3.527) | 7.337 (4.592) | -1.08 | Up | 0.83234 |
| PC aa C34:4 | 2.464 (1.215) | 2.689 (1.463) | -1.09 | Up | 0.83608 |
| Cys | 88.805 (65.665) | 81.447 (36.502) | 1.09 | Down | 0.83609 |
| PC aa C40:5 | 9.296 (3.650) | 9.616 (3.599) | -1.03 | Up | 0.83609 |
| TG(16:1_36:2) | 55.501 (28.219) | 59.944 (36.270) | -1.08 | Up | 0.83609 |
| BABA | 0.028 (0.032) | 0.028 (0.028) | -1.02 | Up | 0.8391 |
| Asp | 15.030 (5.120) | 15.793 (6.393) | -1.05 | Up | 0.83983 |
| Asn | 49.678 (26.807) | 47.544 (16.583) | 1.04 | Down | 0.83985 |
| Orn | 33.986 (16.133) | 34.744 (14.654) | -1.02 | Up | 0.84361 |
| PC ae C44:4 | 0.441 (0.179) | 0.447 (0.156) | -1.02 | Up | 0.85624 |
| C5-M-DC | 0.040 (0.029) | 0.039 (0.024) | 1.02 | Down | 0.86118 |
| DG(18:1_20:0) | 0.238 (0.159) | 0.249 (0.170) | -1.04 | Up | 0.86264 |
| Methionine | 14.377 (6.147) | 13.973 (5.650) | 1.03 | Down | 0.86626 |
| Creatinine.1 | 49.872 (15.949) | 52.576 (15.648) | -1.05 | Up | 0.86627 |
| PC ae C44:5 | 1.607 (0.673) | 1.631 (0.566) | -1.01 | Up | 0.86808 |
| TG(18:3_35:2) | 0.938 (0.616) | 0.931 (0.473) | 1.01 | Down | 0.87005 |
| DG(16:0_20:3) | 0.068 (0.038) | 0.075 (0.037) | -1.1 | Up | 0.87356 |
| AconAcid | 2.125 (1.300) | 2.362 (2.177) | -1.11 | Up | 0.87762 |
| TG(17:1_38:5) | 0.208 (0.079) | 0.211 (0.081) | -1.01 | Up | 0.88034 |
| DG(16:1_18:1) | 1.198 (0.674) | 1.263 (0.572) | -1.05 | Up | 0.88139 |
| Acetoacetate | 47.650 (48.909) | 75.195 (132.650) | -1.58 | Up | 0.88523 |
| Taurine | 49.340 (29.823) | 49.356 (24.948) | -1 | Up | 0.88903 |
| lysoPC a C18:2 | 10.092 (7.108) | 9.284 (4.550) | 1.09 | Down | 0.88903 |
| C5-OH (C3-DC-M) | 0.069 (0.046) | 0.068 (0.050) | 1.01 | Down | 0.89116 |
| Glycerol | 137.865 (46.221) | 135.780 (45.071) | 1.02 | Down | 0.89284 |
| L-Phenylalanine | 32.790 (13.510) | 32.410 (12.238) | 1.01 | Down | 0.89545 |
| PC ae C38:1 | 0.105 (0.183) | 0.082 (0.129) | 1.29 | Down | 0.9031 |
| DiCA(14:0) | 0.056 (0.059) | 0.055 (0.057) | 1 | Down | 0.9043 |
| 1-Met-His | 2.958 (1.466) | 3.117 (1.476) | -1.05 | Up | 0.90807 |
| Hex2Cer(d18:1/26:0) | 0.017 (0.009) | 0.017 (0.009) | -1.02 | Up | 0.91158 |
| GABA | 0.192 (0.074) | 0.192 (0.057) | 1 | Down | 0.91188 |
| alpha-AAA | 0.891 (0.470) | 0.880 (0.417) | 1.01 | Down | 0.91284 |
| TG(20:2_34:4) | 0.119 (0.079) | 0.114 (0.060) | 1.05 | Down | 0.91569 |
| TG(17:2_36:2) | 0.564 (0.260) | 0.580 (0.219) | -1.03 | Up | 0.9157 |
| C6:1 | 0.024 (0.020) | 0.024 (0.020) | 1.01 | Down | 0.9166 |
| PC ae C30:1 | 0.171 (0.109) | 0.173 (0.105) | -1.01 | Up | 0.91795 |
| DG(22:1_22:2) | 0.021 (0.021) | 0.020 (0.020) | 1 | Down | 0.92208 |
| DG-O(16:0_20:4) | 0.005 (0.005) | 0.004 (0.005) | 1.05 | Down | 0.92659 |
| TG(18:3_36:2) | 18.527 (12.843) | 18.054 (8.564) | 1.03 | Down | 0.92715 |
| lysoPC a C20:3 | 0.856 (0.604) | 0.801 (0.427) | 1.07 | Down | 0.92716 |
| C5:1 | 0.046 (0.030) | 0.047 (0.031) | -1.02 | Up | 0.93043 |
| Glu | 56.421 (28.019) | 56.947 (27.231) | -1.01 | Up | 0.93237 |
| TG(16:1_36:1) | 11.502 (5.683) | 12.318 (6.170) | -1.07 | Up | 0.93863 |
| 2-Hydroxybutyric acid | 20.980 (13.916) | 20.685 (14.173) | 1.01 | Down | 0.93864 |
| PC aa C36:0 | 1.335 (0.718) | 1.360 (0.695) | -1.02 | Up | 0.94246 |
| PC aa C40:4 | 3.929 (1.461) | 4.104 (1.383) | -1.04 | Up | 0.94629 |
| TG(18:1_36:6) | 1.710 (1.521) | 1.525 (0.878) | 1.12 | Down | 0.94629 |
| C7-DC | 0.034 (0.028) | 0.035 (0.030) | -1.02 | Up | 0.94639 |
| DG(17:0_17:1) | 0.105 (0.070) | 0.112 (0.056) | -1.06 | Up | 0.94988 |
| Urea | 130.165 (149.485) | 148.268 (219.892) | -1.14 | Up | 0.95013 |
| TG(17:2_38:7) | 0.168 (0.138) | 0.159 (0.096) | 1.06 | Down | 0.95762 |
| C5-DC (C6-OH) | 0.039 (0.036) | 0.039 (0.036) | 1 | Down | 0.95939 |
| C14:2-OH | 0.021 (0.013) | 0.021 (0.014) | 1.02 | Down | 0.96118 |
| C5:1-DC | 0.030 (0.027) | 0.028 (0.025) | 1.04 | Down | 0.96815 |
| DG(14:0_20:0) | 0.052 (0.034) | 0.054 (0.030) | -1.03 | Up | 0.96913 |
| lysoPC a C28:0 | 0.167 (0.140) | 0.176 (0.150) | -1.05 | Up | 0.97959 |
| C5 | 0.109 (0.037) | 0.111 (0.038) | -1.02 | Up | 0.9808 |
| Ile | 51.385 (24.255) | 52.583 (23.115) | -1.02 | Up | 0.98081 |
| PC ae C36:4 | 19.691 (7.251) | 20.152 (5.107) | -1.02 | Up | 0.98081 |
| lysoPC a C26:1 | 0.110 (0.055) | 0.110 (0.046) | 1 | Down | 0.98464 |
| Leu | 84.445 (38.176) | 85.752 (36.163) | -1.02 | Up | 0.98848 |
| TG(18:1_36:2) | 242.677 (127.831) | 247.758 (126.784) | -1.02 | Up | 1 |

**Supplemental table S9. Univariate analysis of metabolites / lipids, Early vs Late Preeclampsia**

| Name | Mean (SD) of EarlyPre | Mean (SD) of LatePre | Fold Change | LatePre vs EarlyPre | p-value |
| --- | --- | --- | --- | --- | --- |
| lysoPC a C16:0 | 56.931 (21.160) | 39.569 (16.074) | -1.44 | Up | **< 0.0001 (W)** |
| EPA | 0.066 (0.217) | 0.185 (0.268) | 2.82 | Down | **0.0001 (W)** |
| lysoPC a C18:1 | 7.820 (3.200) | 5.568 (2.732) | -1.4 | Up | **0.0002 (W)** |
| DG(18:1_20:3) | 0.245 (0.087) | 0.143 (0.116) | -1.71 | Up | **0.0003 (W)** |
| lysoPC a C16:1 | 1.276 (0.728) | 0.836 (0.478) | -1.53 | Up | **0.0003 (W)** |
| lysoPC a C20:4 | 2.851 (1.267) | 1.934 (1.036) | -1.47 | Up | **0.0003 (W)** |
| Histamine | 0.003 (0.004) | 0.011 (0.013) | 3.84 | Down | **0.0003 (W)** |
| lysoPC a C17:0 | 0.732 (0.255) | 0.535 (0.209) | -1.37 | Up | **0.0004** |
| C5-DC (C6-OH) | 0.070 (0.026) | 0.039 (0.036) | -1.78 | Up | **0.0005 (W)** |
| lysoPC a C18:0 | 12.266 (4.714) | 8.915 (3.570) | -1.38 | Up | **0.0011 (W)** |
| TG(17:2_38:7) | 0.223 (0.095) | 0.159 (0.095) | -1.4 | Up | **0.0011 (W)** |
| C16-OH | 0.033 (0.016) | 0.019 (0.018) | -1.75 | Up | **0.0013 (W)** |
| C5-OH (C3-DC-M) | 0.101 (0.035) | 0.068 (0.050) | -1.47 | Up | **0.0025 (W)** |
| lysoPC a C20:3 | 1.134 (0.573) | 0.802 (0.425) | -1.41 | Up | **0.0027 (W)** |
| TG(20:4_32:1) | 10.477 (6.741) | 6.850 (4.986) | -1.53 | Up | **0.0042 (W)** |
| Cer(d18:1/26:1) | 0.031 (0.010) | 0.025 (0.008) | -1.26 | Up | **0.0042** |
| C5-M-DC | 0.056 (0.021) | 0.039 (0.024) | -1.43 | Up | **0.0050 (W)** |
| FA(20:1) | 1.180 (2.055) | 2.493 (2.698) | 2.11 | Down | **0.0057 (W)** |
| Cer(d18:2/14:0) | 0.002 (0.002) | 0.003 (0.001) | 1.56 | Down | **0.0059 (W)** |
| C6:1 | 0.038 (0.016) | 0.024 (0.020) | -1.62 | Up | **0.0063 (W)** |
| C5:1-DC | 0.046 (0.019) | 0.029 (0.025) | -1.59 | Up | **0.0064 (W)** |
| TG(22:4_32:0) | 2.908 (1.705) | 1.980 (1.301) | -1.47 | Up | **0.0064 (W)** |
| TLCA | 0.001 (0.003) | 0.002 (0.005) | 2.27 | Down | **0.0069 (W)** |
| TG(20:4_33:2) | 0.511 (0.210) | 0.381 (0.202) | -1.34 | Up | **0.0075 (W)** |
| Cer(d18:0/18:0) | 0.036 (0.023) | 0.023 (0.020) | -1.61 | Up | **0.0079 (W)** |
| Gly | 192.144 (58.254) | 159.234 (48.889) | -1.21 | Up | **0.0080 (W)** |
| DiCA(14:0) | 0.030 (0.025) | 0.057 (0.056) | 1.89 | Down | **0.0082 (W)** |
| TG(20:4_34:1) | 44.147 (18.859) | 32.614 (15.376) | -1.35 | Up | **0.0087 (W)** |
| TG(20:4_32:0) | 16.337 (9.504) | 11.485 (7.645) | -1.42 | Up | **0.0089 (W)** |
| FA(20:3) | 0.442 (0.951) | 0.851 (1.002) | 1.93 | Down | **0.0090 (W)** |
| TG(20:4_34:2) | 32.825 (12.317) | 25.484 (11.604) | -1.29 | Up | **0.0092** |
| TG(20:4_32:2) | 3.006 (1.548) | 2.161 (1.366) | -1.39 | Up | **0.0096 (W)** |
| TG(22:4_32:2) | 0.179 (0.084) | 0.135 (0.079) | -1.33 | Up | **0.0122 (W)** |
| TG(20:4_36:2) | 17.286 (6.309) | 13.746 (5.705) | -1.26 | Up | **0.0122** |
| TG(20:4_34:0) | 6.319 (2.717) | 4.763 (2.209) | -1.33 | Up | **0.0127 (W)** |
| Creatine | 31.553 (16.376) | 22.040 (11.949) | -1.43 | Up | **0.0129 (W)** |
| TG(16:0_38:5) | 21.508 (8.197) | 16.898 (7.633) | -1.27 | Up | **0.0131 (W)** |
| Hypoxanthine.1 | 8.720 (5.196) | 5.863 (3.739) | -1.49 | Up | **0.0135 (W)** |
| DG(22:1_22:2) | 0.032 (0.022) | 0.021 (0.020) | -1.57 | Up | **0.0135 (W)** |
| TG(20:3_32:1) | 5.155 (3.232) | 3.427 (1.955) | -1.5 | Up | **0.0139 (W)** |
| lysoPC a C18:2 | 12.222 (5.575) | 9.289 (4.539) | -1.32 | Up | **0.0145 (W)** |
| TG(16:0_40:6) | 7.555 (3.035) | 5.829 (2.662) | -1.3 | Up | **0.0151 (W)** |
| DG(16:0_20:0) | 0.804 (0.260) | 0.654 (0.308) | -1.23 | Up | **0.0156 (W)** |
| Cortisone | 0.075 (0.047) | 0.106 (0.069) | 1.41 | Down | **0.0161 (W)** |
| DG(18:1_20:4) | 0.558 (0.138) | 0.453 (0.191) | -1.23 | Up | **0.0162 (W)** |
| TG(20:4_30:0) | 3.913 (2.860) | 2.722 (2.399) | -1.44 | Up | **0.0177 (W)** |
| Pyruvic acid | 105.789 (56.731) | 82.895 (65.709) | -1.28 | Up | **0.0189 (W)** |
| TG(20:3_34:0) | 2.374 (1.031) | 1.785 (0.752) | -1.33 | Up | **0.0195 (W)** |
| TG(22:4_34:2) | 4.890 (2.017) | 3.832 (1.871) | -1.28 | Up | **0.0201 (W)** |
| DiCA(12:0) | 0.083 (0.054) | 0.142 (0.137) | 1.7 | Down | **0.0210 (W)** |
| DCA | 0.258 (0.193) | 0.166 (0.161) | -1.55 | Up | **0.0221 (W)** |
| TG(20:4_34:3) | 4.104 (1.544) | 3.262 (1.591) | -1.26 | Up | **0.0223** |
| TG(20:4_36:3) | 13.322 (4.407) | 10.944 (4.496) | -1.22 | Up | **0.0229** |
| TG(20:3_34:2) | 13.036 (5.050) | 10.374 (4.464) | -1.26 | Up | **0.0230 (W)** |
| TG(16:0_38:6) | 11.123 (4.084) | 8.967 (4.032) | -1.24 | Up | **0.0235** |
| Met-SO | 0.352 (0.331) | 0.250 (0.507) | -1.41 | Up | **0.0237 (W)** |
| TG(16:1_30:1) | 5.110 (4.742) | 2.962 (2.521) | -1.73 | Up | **0.0246 (W)** |
| TG(18:1_38:5) | 11.968 (4.675) | 9.522 (3.919) | -1.26 | Up | **0.0260 (W)** |
| TG(14:0_38:5) | 1.523 (0.779) | 1.173 (0.757) | -1.3 | Up | **0.0263 (W)** |
| TG(16:1_28:0) | 3.142 (2.759) | 2.049 (2.329) | -1.53 | Up | **0.0274 (W)** |
| C7-DC | 0.054 (0.019) | 0.035 (0.030) | -1.53 | Up | **0.0282 (W)** |
| TG(20:2_34:4) | 0.143 (0.055) | 0.114 (0.059) | -1.26 | Up | **0.0289 (W)** |
| TG(16:1_38:5) | 2.677 (1.328) | 1.964 (0.946) | -1.36 | Up | **0.0289 (W)** |
| Carnitine | 11.678 (8.247) | 10.650 (12.591) | -1.1 | Up | **0.0297 (W)** |
| TG(16:1_32:2) | 10.835 (8.329) | 7.442 (5.038) | -1.46 | Up | **0.0297 (W)** |
| L-Phenylalanine | 39.056 (13.895) | 32.410 (12.238) | -1.21 | Up | **0.0297** |
| Methionine | 17.306 (7.438) | 13.973 (5.650) | -1.24 | Up | **0.03** |
| Methanol | 396.767 (210.809) | 349.053 (496.200) | -1.14 | Up | **0.0301 (W)** |
| TG(20:3_32:2) | 1.407 (0.750) | 1.028 (0.530) | -1.37 | Up | **0.0305 (W)** |
| HArg | 4.954 (2.405) | 3.936 (1.418) | -1.26 | Up | **0.0308** |
| TG(20:3_34:3) | 1.982 (0.768) | 1.574 (0.714) | -1.26 | Up | **0.0313 (W)** |
| GABA | 0.223 (0.069) | 0.192 (0.057) | -1.16 | Up | **0.0317 (W)** |
| TG(16:0_32:3) | 9.297 (4.565) | 7.529 (5.532) | -1.23 | Up | **0.0321 (W)** |
| TG(18:2_31:0) | 5.821 (2.258) | 4.794 (2.604) | -1.21 | Up | **0.0347 (W)** |
| TG(22:5_34:2) | 11.087 (4.549) | 8.982 (3.994) | -1.23 | Up | **0.035** |
| TG(20:3_32:0) | 7.131 (4.203) | 5.149 (2.802) | -1.39 | Up | **0.0361 (W)** |
| TG(22:5_32:1) | 3.657 (2.272) | 2.570 (1.518) | -1.42 | Up | **0.0365 (W)** |
| PC ae C34:2 | 16.171 (4.752) | 18.097 (4.989) | 1.12 | Down | **0.0370 (W)** |
| C14 | 0.131 (0.064) | 0.096 (0.069) | -1.35 | Up | **0.0370 (W)** |
| PC aa C38:5 | 62.761 (20.589) | 53.152 (17.830) | -1.18 | Up | **0.0379 (W)** |
| TG(20:5_34:1) | 3.023 (1.493) | 2.433 (1.726) | -1.24 | Up | **0.0384 (W)** |
| C18 | 0.036 (0.023) | 0.027 (0.022) | -1.36 | Up | **0.0385 (W)** |
| TG(18:1_31:0) | 8.113 (3.647) | 6.505 (3.519) | -1.25 | Up | **0.0389 (W)** |
| TG(22:5_34:3) | 1.762 (0.701) | 1.414 (0.653) | -1.25 | Up | **0.0389 (W)** |
| TG(17:0_34:3) | 1.736 (0.734) | 1.420 (0.697) | -1.22 | Up | **0.0404 (W)** |
| C16:1-OH | 0.019 (0.013) | 0.014 (0.012) | -1.41 | Up | **0.0405 (W)** |
| DG(16:1_20:0) | 0.390 (0.494) | 0.174 (0.155) | -2.25 | Up | **0.0423 (W)** |
| TG(16:0_34:4) | 9.509 (4.035) | 7.833 (4.655) | -1.21 | Up | **0.0430 (W)** |
| PC ae C34:3 | 9.553 (3.993) | 11.079 (3.928) | 1.16 | Down | **0.0436 (W)** |
| TG(16:1_32:0) | 47.931 (36.036) | 33.391 (24.714) | -1.44 | Up | **0.0436 (W)** |
| TG(16:0_28:1) | 7.682 (7.019) | 5.899 (7.091) | -1.3 | Up | **0.0441 (W)** |
| TG(20:5_34:0) | 0.620 (0.321) | 0.492 (0.342) | -1.26 | Up | **0.0441 (W)** |
| TG(22:5_34:1) | 13.123 (6.007) | 10.064 (4.777) | -1.3 | Up | **0.0441 (W)** |
| lysoPC a C24:0 | 0.143 (0.046) | 0.123 (0.045) | -1.16 | Up | **0.0446 (W)** |
| DG(18:2_20:4) | 0.498 (0.153) | 0.422 (0.169) | -1.18 | Up | **0.0447** |
| TG(18:1_38:7) | 1.393 (0.478) | 1.161 (0.518) | -1.2 | Up | **0.0468** |
| TG(16:1_38:4) | 3.156 (1.633) | 2.342 (1.089) | -1.35 | Up | **0.0469 (W)** |
| TG(16:0_32:2) | 74.089 (41.257) | 56.190 (36.366) | -1.32 | Up | **0.0475 (W)** |
| DG(16:0_16:1) | 1.572 (0.878) | 1.152 (0.578) | -1.36 | Up | **0.0475 (W)** |
| TG(16:1_32:1) | 45.072 (40.258) | 27.881 (21.011) | -1.62 | Up | **0.0487 (W)** |
| TG(16:1_34:2) | 104.500 (54.169) | 84.651 (51.301) | -1.23 | Up | 0.0511 (W) |
| TG(18:0_38:6) | 2.137 (1.053) | 1.693 (0.678) | -1.26 | Up | 0.0511 (W) |
| TG(16:1_33:1) | 4.595 (2.979) | 3.266 (1.997) | -1.41 | Up | 0.0517 (W) |
| TG(22:1_32:5) | 0.084 (0.039) | 0.070 (0.037) | -1.2 | Up | 0.0523 (W) |
| TG(20:4_36:5) | 1.273 (0.677) | 1.012 (0.630) | -1.26 | Up | 0.0536 (W) |
| PC aa C40:5 | 11.393 (4.162) | 9.627 (3.571) | -1.18 | Up | 0.0543 (W) |
| TG(14:0_38:4) | 1.949 (0.916) | 1.536 (0.821) | -1.27 | Up | 0.0556 (W) |
| TG(18:2_38:5) | 7.288 (2.657) | 6.235 (2.563) | -1.17 | Up | 0.0563 (W) |
| TG(18:1_28:1) | 4.119 (2.547) | 3.607 (4.105) | -1.14 | Up | 0.0563 (W) |
| TG(22:5_32:0) | 5.706 (3.168) | 4.356 (2.461) | -1.31 | Up | 0.0583 (W) |
| Cer(d16:1/20:0) | 0.075 (0.023) | 0.065 (0.025) | -1.15 | Up | 0.0589 (W) |
| Trp | 43.703 (12.095) | 38.615 (11.278) | -1.13 | Up | 0.0617 |
| Ethanol | 135.994 (85.964) | 190.643 (118.897) | 1.4 | Down | 0.0618 (W) |
| Glycerol | 154.689 (46.933) | 135.780 (45.071) | -1.14 | Up | 0.0618 (W) |
| TG(17:2_36:2) | 0.706 (0.278) | 0.580 (0.218) | -1.22 | Up | 0.0618 (W) |
| TG(20:3_34:1) | 17.590 (7.987) | 13.596 (5.874) | -1.29 | Up | 0.0626 (W) |
| TG(18:0_38:7) | 0.402 (0.149) | 0.333 (0.142) | -1.21 | Up | 0.0626 (W) |
| TG(16:1_34:3) | 16.703 (9.815) | 13.081 (8.280) | -1.28 | Up | 0.0633 (W) |
| TG(18:1_30:1) | 34.941 (22.270) | 27.071 (18.596) | -1.29 | Up | 0.0633 (W) |
| Choline.1 | 9.516 (3.092) | 8.236 (2.846) | -1.16 | Up | 0.0641 |
| DG(16:1_18:1) | 1.509 (0.565) | 1.263 (0.572) | -1.19 | Up | 0.0645 |
| TG(16:0_32:1) | 183.089 (123.621) | 135.444 (91.734) | -1.35 | Up | 0.0648 (W) |
| PC ae C36:3 | 9.107 (2.273) | 9.970 (2.532) | 1.09 | Down | 0.0655 (W) |
| CE(22:5) | 5.549 (2.138) | 4.609 (1.888) | -1.2 | Up | 0.0671 (W) |
| TG(20:5_34:2) | 2.356 (1.180) | 1.892 (1.203) | -1.25 | Up | 0.0671 (W) |
| Citric acid | 58.842 (53.416) | 66.570 (34.952) | 1.13 | Down | 0.0679 (W) |
| TG(14:0_32:2) | 7.138 (4.815) | 5.560 (4.930) | -1.28 | Up | 0.0679 (W) |
| TG(14:0_34:3) | 9.690 (4.969) | 7.907 (5.554) | -1.23 | Up | 0.0679 (W) |
| DG(16:0_20:3) | 0.086 (0.033) | 0.075 (0.037) | -1.15 | Up | 0.0686 (W) |
| CE(20:0) | 1.715 (2.358) | 1.936 (1.019) | 1.13 | Down | 0.0689 (W) |
| FA(20:2) | 0.871 (1.404) | 1.248 (1.312) | 1.43 | Down | 0.0700 (W) |
| TG(18:3_38:5) | 0.817 (0.283) | 0.721 (0.367) | -1.13 | Up | 0.0703 (W) |
| PC aa C40:4 | 4.885 (1.768) | 4.110 (1.366) | -1.19 | Up | 0.0711 (W) |
| TG(14:0_34:2) | 52.856 (25.979) | 42.703 (26.782) | -1.24 | Up | 0.0711 (W) |
| H1 | 4908.861 (1103.415) | 4728.985 (2070.812) | -1.04 | Up | 0.0727 (W) |
| TG(16:0_34:3) | 120.086 (50.158) | 103.227 (59.331) | -1.16 | Up | 0.0735 (W) |
| HipAcid | 2.691 (3.855) | 1.357 (0.983) | -1.98 | Up | 0.0744 (W) |
| Kynurenine | 1.570 (0.379) | 1.390 (0.350) | -1.13 | Up | 0.0752 (W) |
| CDCA | 0.270 (0.214) | 0.187 (0.172) | -1.44 | Up | 0.0752 (W) |
| PC aa C38:4 | 132.944 (45.914) | 116.397 (34.058) | -1.14 | Up | 0.0766 |
| Cer(d18:1/18:1) | 0.022 (0.012) | 0.026 (0.011) | 1.19 | Down | 0.0774 (W) |
| ADMA | 0.412 (0.093) | 0.370 (0.089) | -1.11 | Up | 0.0778 (W) |
| DG(18:3_18:3) | 0.043 (0.045) | 0.027 (0.035) | -1.63 | Up | 0.0780 (W) |
| TG(17:0_32:1) | 3.198 (1.926) | 2.515 (1.723) | -1.27 | Up | 0.0796 (W) |
| TG(18:1_32:3) | 6.854 (2.919) | 6.037 (3.267) | -1.14 | Up | 0.0796 (W) |
| TG(16:0_32:0) | 152.497 (77.512) | 123.153 (66.430) | -1.24 | Up | 0.0797 |
| TG(20:4_36:4) | 5.740 (2.265) | 4.801 (2.336) | -1.2 | Up | 0.08 |
| Ala | 418.333 (140.850) | 371.390 (146.591) | -1.13 | Up | 0.0805 (W) |
| C14:2-OH | 0.026 (0.010) | 0.021 (0.014) | -1.28 | Up | 0.0858 (W) |
| CE(18:3) | 99.811 (49.326) | 85.856 (43.054) | -1.16 | Up | 0.0860 (W) |
| TG(16:0_34:2) | 536.028 (226.482) | 460.530 (243.754) | -1.16 | Up | 0.0861 (W) |
| TG(16:0_38:4) | 23.531 (9.231) | 19.740 (8.528) | -1.19 | Up | 0.0880 (W) |
| TG(16:1_34:0) | 21.963 (13.384) | 16.711 (9.815) | -1.31 | Up | 0.0880 (W) |
| C3:1 | 0.027 (0.009) | 0.023 (0.012) | -1.21 | Up | 0.0882 (W) |
| Arg | 80.292 (17.292) | 71.085 (19.202) | -1.13 | Up | 0.0889 (W) |
| TG(16:0_33:2) | 9.014 (3.893) | 7.643 (4.232) | -1.18 | Up | 0.0899 (W) |
| TG(16:1_36:1) | 15.503 (8.149) | 12.328 (6.150) | -1.26 | Up | 0.0909 (W) |
| TG(18:3_32:1) | 11.530 (5.205) | 9.860 (6.231) | -1.17 | Up | 0.0909 (W) |
| CE(16:0) | 333.722 (73.295) | 298.925 (74.583) | -1.12 | Up | 0.0929 (W) |
| TG(18:0_32:1) | 15.297 (10.098) | 12.176 (8.258) | -1.26 | Up | 0.0929 (W) |
| TG(16:1_34:1) | 168.006 (99.934) | 131.139 (82.151) | -1.28 | Up | 0.0939 (W) |
| TG(16:0_38:7) | 1.968 (0.877) | 1.670 (0.984) | -1.18 | Up | 0.0960 (W) |
| TG(22:6_32:1) | 4.938 (3.098) | 3.951 (2.885) | -1.25 | Up | 0.0960 (W) |
| Cer(d18:0/18:0(OH)) | 0.434 (0.269) | 0.317 (0.231) | -1.37 | Up | 0.0962 (W) |
| TG(20:5_36:2) | 1.282 (0.511) | 1.128 (0.696) | -1.14 | Up | 0.0970 (W) |
| TG(18:3_30:0) | 4.342 (2.483) | 3.837 (3.360) | -1.13 | Up | 0.0981 (W) |
| TG(14:0_35:1) | 1.842 (1.075) | 1.546 (1.320) | -1.19 | Up | 0.0992 (W) |
| TG(20:3_36:3) | 4.061 (1.461) | 3.436 (1.247) | -1.18 | Up | 0.0992 (W) |
| TG(16:0_35:1) | 14.617 (7.135) | 12.128 (6.215) | -1.21 | Up | 0.1002 (W) |
| CE(14:1) | 1.585 (1.145) | 1.276 (0.912) | -1.24 | Up | 0.1013 (W) |
| TG(17:1_32:1) | 3.065 (1.914) | 2.320 (1.416) | -1.32 | Up | 0.1013 (W) |
| CE(17:1) | 9.193 (3.005) | 8.089 (2.811) | -1.14 | Up | 0.1023 |
| TG(14:0_34:1) | 80.400 (45.961) | 65.045 (41.745) | -1.24 | Up | 0.1024 (W) |
| DG-O(16:0_18:1) | 0.136 (0.053) | 0.107 (0.065) | -1.27 | Up | 0.1040 (W) |
| GDCA | 0.734 (0.678) | 0.492 (0.494) | -1.49 | Up | 0.1057 (W) |
| TG(16:0_30:2) | 8.660 (5.428) | 6.884 (5.090) | -1.26 | Up | 0.1057 (W) |
| Propylene glycol | 0.432 (0.410) | 0.324 (0.425) | -1.33 | Up | 0.1065 (W) |
| TG(18:3_38:6) | 0.540 (0.231) | 0.454 (0.250) | -1.19 | Up | 0.1068 (W) |
| Cer(d18:0/22:0) | 0.126 (0.073) | 0.154 (0.064) | 1.23 | Down | 0.1077 (W) |
| TG(17:1_38:5) | 0.248 (0.100) | 0.211 (0.081) | -1.18 | Up | 0.1079 (W) |
| TG(18:0_32:2) | 5.200 (2.586) | 4.506 (2.939) | -1.15 | Up | 0.1091 (W) |
| TG(20:4_35:3) | 0.182 (0.069) | 0.157 (0.067) | -1.16 | Up | 0.1091 |
| Isopropyl alcohol | 8.989 (6.501) | 13.207 (11.241) | 1.47 | Down | 0.1114 (W) |
| CE(17:0) | 6.873 (1.994) | 6.114 (2.106) | -1.12 | Up | 0.112 |
| Val | 102.072 (44.717) | 83.790 (38.235) | -1.22 | Up | 0.1138 (W) |
| TG(14:0_34:0) | 10.999 (6.575) | 9.258 (6.686) | -1.19 | Up | 0.1150 (W) |
| TG(18:0_34:2) | 46.036 (19.329) | 40.559 (20.854) | -1.14 | Up | 0.1150 (W) |
| PC ae C36:2 | 18.850 (5.480) | 19.785 (4.765) | 1.05 | Down | 0.1162 (W) |
| TG(16:0_28:2) | 2.607 (1.951) | 2.290 (2.509) | -1.14 | Up | 0.1174 (W) |
| TG(18:2_38:4) | 7.055 (2.895) | 6.083 (2.631) | -1.16 | Up | 0.1174 (W) |
| CE(20:4) | 404.806 (154.000) | 354.105 (125.133) | -1.14 | Up | 0.118 |
| TG(20:1_32:2) | 1.549 (0.684) | 1.315 (0.585) | -1.18 | Up | 0.1186 (W) |
| TG(18:2_30:0) | 47.669 (21.490) | 41.582 (26.401) | -1.15 | Up | 0.1186 (W) |
| FA(18:1) | 109.322 (112.068) | 161.988 (141.015) | 1.48 | Down | 0.1202 (W) |
| 3-Hydroxybutyric acid | 85.039 (174.525) | 207.050 (461.829) | 2.43 | Down | 0.1224 (W) |
| Cer(d18:0/24:1) | 0.184 (0.063) | 0.207 (0.063) | 1.12 | Down | 0.1233 |
| Acetic acid | 10.764 (4.799) | 20.802 (35.310) | 1.93 | Down | 0.1262 (W) |
| Ser | 93.022 (20.912) | 86.420 (26.585) | -1.08 | Up | 0.1262 (W) |
| TG(18:2_32:1) | 137.044 (57.156) | 120.450 (66.237) | -1.14 | Up | 0.1274 (W) |
| TG(16:0_36:6) | 2.087 (0.846) | 1.929 (1.235) | -1.08 | Up | 0.1275 (W) |
| TG(20:2_32:1) | 4.491 (2.259) | 3.529 (1.665) | -1.27 | Up | 0.1288 (W) |
| TG(18:1_36:6) | 1.678 (0.682) | 1.526 (0.876) | -1.1 | Up | 0.1301 (W) |
| TG(20:1_30:1) | 0.308 (0.210) | 0.242 (0.122) | -1.27 | Up | 0.1313 (W) |
| CE(16:1) | 129.414 (70.618) | 103.994 (53.370) | -1.24 | Up | 0.1328 (W) |
| lysoPC a C28:1 | 0.567 (0.243) | 0.483 (0.214) | -1.17 | Up | 0.1328 (W) |
| TG(18:0_36:1) | 9.805 (5.356) | 7.964 (4.513) | -1.23 | Up | 0.1328 (W) |
| Glu | 67.731 (34.610) | 56.947 (27.231) | -1.19 | Up | 0.1335 |
| Acetoacetate | 33.225 (45.303) | 75.195 (132.650) | 2.26 | Down | 0.1341 (W) |
| TG(17:1_34:3) | 1.251 (0.577) | 1.062 (0.513) | -1.18 | Up | 0.1343 |
| C5:1 | 0.059 (0.019) | 0.047 (0.031) | -1.26 | Up | 0.1346 (W) |
| TG(18:2_30:1) | 16.581 (8.588) | 14.120 (8.687) | -1.17 | Up | 0.1355 (W) |
| L-Lactic acid | 1882.097 (819.197) | 1635.595 (848.017) | -1.15 | Up | 0.1361 (W) |
| CE(18:1) | 585.917 (166.949) | 527.190 (172.390) | -1.11 | Up | 0.1365 |
| C3-DC (C4-OH) | 0.105 (0.028) | 0.094 (0.033) | -1.11 | Up | 0.1375 |
| TG(18:1_30:0) | 91.994 (52.686) | 75.722 (48.678) | -1.21 | Up | 0.1382 (W) |
| DG(14:1_20:2) | 0.056 (0.035) | 0.045 (0.042) | -1.26 | Up | 0.1399 (W) |
| TG(17:1_36:5) | 0.342 (0.135) | 0.307 (0.154) | -1.11 | Up | 0.1410 (W) |
| TG(17:0_34:2) | 8.383 (3.431) | 7.371 (3.436) | -1.14 | Up | 0.1438 (W) |
| TG(18:1_32:1) | 279.019 (154.101) | 226.910 (131.525) | -1.23 | Up | 0.1452 (W) |
| TG(16:0_33:1) | 18.772 (9.814) | 15.611 (9.102) | -1.2 | Up | 0.1453 (W) |
| DG(17:0_18:1) | 0.820 (0.268) | 0.729 (0.271) | -1.12 | Up | 0.1461 |
| HexCer(d18:1/18:0) | 0.281 (0.114) | 0.302 (0.096) | 1.07 | Down | 0.1467 (W) |
| DG(16:1_18:2) | 1.729 (0.751) | 1.532 (0.884) | -1.13 | Up | 0.1481 (W) |
| TG(18:3_32:0) | 16.149 (7.031) | 14.337 (8.425) | -1.13 | Up | 0.1482 (W) |
| PC ae C42:3 | 0.891 (0.253) | 0.989 (0.327) | 1.11 | Down | 0.1496 |
| HexCer(d18:1/18:1) | 0.063 (0.066) | 0.085 (0.066) | 1.34 | Down | 0.1548 (W) |
| TG(16:0_38:1) | 3.445 (1.551) | 2.865 (1.224) | -1.2 | Up | 0.1556 (W) |
| TG(17:1_34:1) | 12.374 (5.951) | 10.344 (5.361) | -1.2 | Up | 0.1556 (W) |
| 2-Hydroxybutyric acid | 25.242 (20.273) | 20.685 (14.173) | -1.22 | Up | 0.1571 (W) |
| TG(20:5_36:3) | 1.184 (0.529) | 1.042 (0.598) | -1.14 | Up | 0.1571 (W) |
| TG(16:0_35:2) | 18.679 (8.317) | 15.907 (7.809) | -1.17 | Up | 0.1602 (W) |
| TG(18:3_33:2) | 0.733 (0.317) | 0.671 (0.433) | -1.09 | Up | 0.1602 (W) |
| TG(18:0_30:0) | 6.048 (4.312) | 5.263 (5.378) | -1.15 | Up | 0.1617 (W) |
| CE(18:0) | 20.719 (5.156) | 19.020 (5.415) | -1.09 | Up | 0.1664 |
| C18:1 | 0.066 (0.043) | 0.050 (0.044) | -1.32 | Up | 0.1672 (W) |
| TG(17:1_34:2) | 8.271 (3.560) | 7.193 (3.626) | -1.15 | Up | 0.1681 (W) |
| TG(16:0_34:0) | 88.594 (41.805) | 75.774 (38.501) | -1.17 | Up | 0.1682 |
| C10:2 | 0.165 (0.071) | 0.124 (0.085) | -1.32 | Up | 0.1684 (W) |
| TG(18:1_33:0) | 12.525 (6.078) | 10.752 (5.595) | -1.16 | Up | 0.1713 (W) |
| Cortisol | 0.468 (0.315) | 0.575 (0.354) | 1.23 | Down | 0.1713 |
| TG(18:0_30:1) | 2.804 (2.358) | 2.401 (2.561) | -1.17 | Up | 0.1713 (W) |
| PC aa C36:4 | 320.833 (98.552) | 292.565 (80.287) | -1.1 | Up | 0.1728 |
| TG(16:0_34:1) | 658.778 (324.073) | 570.265 (307.679) | -1.16 | Up | 0.1729 (W) |
| PC aa C36:2 | 275.500 (59.431) | 286.330 (68.254) | 1.04 | Down | 0.1762 (W) |
| CE(14:0) | 31.986 (16.140) | 27.890 (14.156) | -1.15 | Up | 0.1762 (W) |
| C4:1 | 0.044 (0.015) | 0.039 (0.017) | -1.13 | Up | 0.1791 |
| DG(16:0_18:1) | 9.946 (4.048) | 8.846 (4.353) | -1.12 | Up | 0.1830 (W) |
| FA(18:2) | 138.522 (116.848) | 178.975 (136.118) | 1.29 | Down | 0.1830 (W) |
| DG(14:1_18:1) | 0.151 (0.067) | 0.132 (0.058) | -1.15 | Up | 0.1839 |
| TG(20:3_36:4) | 1.744 (0.625) | 1.513 (0.597) | -1.15 | Up | 0.1847 (W) |
| TG(17:1_38:7) | 0.044 (0.018) | 0.039 (0.018) | -1.14 | Up | 0.1862 (W) |
| PC aa C38:1 | 0.651 (0.547) | 0.820 (0.598) | 1.26 | Down | 0.1893 (W) |
| TG(18:0_34:3) | 8.357 (3.406) | 7.441 (3.484) | -1.12 | Up | 0.1899 (W) |
| PC ae C40:3 | 1.106 (0.224) | 1.188 (0.313) | 1.07 | Down | 0.1925 |
| PC aa C38:0 | 2.807 (0.993) | 3.034 (1.053) | 1.08 | Down | 0.1934 (W) |
| TG(14:0_35:2) | 1.768 (0.841) | 1.527 (0.908) | -1.16 | Up | 0.1934 (W) |
| DG(18:2_18:4) | 0.080 (0.044) | 0.067 (0.043) | -1.18 | Up | 0.1944 (W) |
| TG(18:1_36:0) | 10.519 (4.950) | 8.991 (4.068) | -1.17 | Up | 0.1952 (W) |
| PC aa C32:1 | 36.449 (22.805) | 29.350 (17.848) | -1.24 | Up | 0.1952 (W) |
| TG(20:1_32:1) | 3.575 (2.009) | 2.873 (1.407) | -1.24 | Up | 0.1970 (W) |
| TG(17:0_34:1) | 10.189 (4.798) | 8.905 (4.305) | -1.14 | Up | 0.1989 (W) |
| PC ae C38:2 | 1.966 (0.510) | 2.138 (0.635) | 1.09 | Down | 0.1994 |
| TG(20:2_32:0) | 6.168 (2.798) | 5.351 (2.704) | -1.15 | Up | 0.2001 |
| DG(18:2_20:0) | 0.096 (0.034) | 0.087 (0.028) | -1.11 | Up | 0.2003 |
| PC aa C36:5 | 18.956 (10.299) | 16.476 (9.621) | -1.15 | Up | 0.2007 (W) |
| TG(14:0_36:1) | 11.180 (5.788) | 9.832 (6.124) | -1.14 | Up | 0.2007 (W) |
| PC ae C42:4 | 1.007 (0.271) | 1.104 (0.380) | 1.1 | Down | 0.2024 |
| SM (OH) C22:2 | 10.461 (2.852) | 9.621 (2.329) | -1.09 | Up | 0.2043 (W) |
| C16:2 | 0.029 (0.015) | 0.024 (0.017) | -1.21 | Up | 0.2043 (W) |
| Orn | 37.428 (11.395) | 34.772 (14.592) | -1.08 | Up | 0.2043 (W) |
| Hex3Cer(d18:1/18:0) | 0.167 (0.044) | 0.182 (0.057) | 1.09 | Down | 0.2045 |
| PC ae C44:4 | 0.409 (0.103) | 0.448 (0.155) | 1.09 | Down | 0.2048 |
| HCys | 5.900 (3.044) | 4.769 (1.921) | -1.24 | Up | 0.2062 (W) |
| C3-OH | 0.046 (0.018) | 0.040 (0.025) | -1.13 | Up | 0.2072 (W) |
| Cer(d18:2/22:0) | 0.252 (0.085) | 0.235 (0.071) | -1.07 | Up | 0.2157 (W) |
| Cer(d18:2/18:0) | 0.080 (0.023) | 0.074 (0.020) | -1.08 | Up | 0.2191 |
| Hypoxanthine | 0.090 (0.066) | 0.159 (0.383) | 1.76 | Down | 0.2194 (W) |
| Hex2Cer(d18:1/26:0) | 0.019 (0.008) | 0.017 (0.009) | -1.12 | Up | 0.2203 (W) |
| PC aa C34:2 | 631.222 (94.460) | 651.505 (130.452) | 1.03 | Down | 0.2215 (W) |
| TG(16:0_35:3) | 7.818 (3.107) | 7.059 (3.511) | -1.11 | Up | 0.2215 (W) |
| TG(18:1_32:0) | 398.714 (200.129) | 350.983 (197.313) | -1.14 | Up | 0.2255 (W) |
| TG(18:3_34:0) | 7.999 (2.961) | 7.367 (3.541) | -1.09 | Up | 0.2255 (W) |
| CE(20:5) | 34.503 (21.377) | 30.137 (19.749) | -1.14 | Up | 0.2295 (W) |
| HexCer(d18:1/24:1) | 4.143 (1.292) | 4.401 (1.393) | 1.06 | Down | 0.2295 (W) |
| TG(18:0_32:0) | 17.948 (7.715) | 15.656 (8.673) | -1.15 | Up | 0.2295 |
| PC ae C40:2 | 1.797 (0.495) | 1.835 (0.444) | 1.02 | Down | 0.2315 (W) |
| TG(17:2_38:6) | 0.273 (0.100) | 0.245 (0.101) | -1.11 | Up | 0.2327 |
| TG(16:0_40:7) | 7.084 (3.177) | 6.030 (2.956) | -1.17 | Up | 0.2336 (W) |
| PC aa C38:3 | 64.083 (16.969) | 58.998 (17.061) | -1.09 | Up | 0.2336 (W) |
| TG(16:1_36:2) | 71.917 (41.081) | 59.976 (36.218) | -1.2 | Up | 0.2336 (W) |
| Acetone | 14.758 (18.086) | 22.745 (36.466) | 1.54 | Down | 0.2356 (W) |
| HexCer(d18:2/16:0) | 0.086 (0.025) | 0.082 (0.028) | -1.05 | Up | 0.2376 (W) |
| Hex3Cer(d18:1_22:0) | 0.240 (0.088) | 0.259 (0.087) | 1.08 | Down | 0.2377 (W) |
| TG(18:1_38:6) | 7.006 (2.596) | 6.165 (2.620) | -1.14 | Up | 0.2398 (W) |
| TG(16:1_38:3) | 2.368 (1.337) | 1.961 (0.921) | -1.21 | Up | 0.2418 (W) |
| TG(17:2_34:3) | 0.880 (0.368) | 0.774 (0.364) | -1.14 | Up | 0.2439 (W) |
| TG(20:2_34:1) | 12.410 (5.175) | 11.052 (4.922) | -1.12 | Up | 0.2449 |
| C2 | 2.658 (1.651) | 2.968 (1.815) | 1.12 | Down | 0.2460 (W) |
| CA | 0.103 (0.128) | 0.096 (0.184) | -1.07 | Up | 0.2476 (W) |
| Isobutyric acid | 5.978 (2.481) | 5.327 (2.292) | -1.12 | Up | 0.2481 (W) |
| TG(18:2_32:0) | 217.003 (96.656) | 201.061 (109.390) | -1.08 | Up | 0.2482 (W) |
| Cer(d18:2/18:1) | 0.004 (0.002) | 0.004 (0.002) | -1.12 | Up | 0.2487 |
| DG(18:1_20:0) | 0.306 (0.155) | 0.248 (0.171) | -1.23 | Up | 0.2488 (W) |
| TDCA | 0.304 (0.438) | 0.194 (0.264) | -1.57 | Up | 0.2503 (W) |
| TG(20:3_36:5) | 0.367 (0.134) | 0.330 (0.140) | -1.11 | Up | 0.2503 (W) |
| C18:2 | 0.047 (0.019) | 0.043 (0.017) | -1.11 | Up | 0.2602 |
| C16 | 0.099 (0.024) | 0.092 (0.031) | -1.08 | Up | 0.265 |
| TG(17:2_38:5) | 0.324 (0.127) | 0.284 (0.120) | -1.14 | Up | 0.2656 (W) |
| TG(18:1_33:1) | 28.077 (13.622) | 24.742 (13.652) | -1.13 | Up | 0.2679 (W) |
| TG(18:2_28:0) | 9.071 (5.623) | 8.288 (6.852) | -1.09 | Up | 0.2679 (W) |
| TG(18:1_30:2) | 6.884 (3.996) | 6.294 (4.324) | -1.09 | Up | 0.2701 (W) |
| Cer(d18:0/20:0) | 0.079 (0.029) | 0.068 (0.034) | -1.16 | Up | 0.2720 (W) |
| CE(20:3) | 50.425 (18.898) | 43.761 (15.920) | -1.15 | Up | 0.2769 (W) |
| Cys | 89.386 (33.349) | 81.567 (36.248) | -1.1 | Up | 0.2770 (W) |
| DG(18:1_22:5) | 0.050 (0.018) | 0.044 (0.020) | -1.13 | Up | 0.2812 (W) |
| PC aa C42:0 | 0.649 (0.192) | 0.702 (0.231) | 1.08 | Down | 0.2828 |
| Formate | 17.447 (14.176) | 44.885 (88.374) | 2.57 | Down | 0.2839 (W) |
| TG(18:2_34:0) | 95.900 (40.277) | 89.837 (44.495) | -1.07 | Up | 0.2839 (W) |
| TrpBetaine | 0.513 (0.533) | 0.462 (0.649) | -1.11 | Up | 0.2863 (W) |
| Cer(d16:1/22:0) | 0.365 (0.141) | 0.337 (0.139) | -1.08 | Up | 0.2863 (W) |
| TG(18:1_33:3) | 1.103 (0.436) | 1.009 (0.489) | -1.09 | Up | 0.2886 (W) |
| TG(17:2_34:2) | 0.805 (0.289) | 0.753 (0.353) | -1.07 | Up | 0.2886 (W) |
| PC aa C34:4 | 3.081 (1.550) | 2.691 (1.458) | -1.14 | Up | 0.2910 (W) |
| TG(20:1_34:0) | 1.773 (0.752) | 1.627 (0.781) | -1.09 | Up | 0.2934 (W) |
| PC ae C30:2 | 0.118 (0.033) | 0.111 (0.028) | -1.07 | Up | 0.2957 (W) |
| HexCer(d18:1/22:0) | 3.908 (1.509) | 4.038 (1.223) | 1.03 | Down | 0.2957 (W) |
| TG(22:6_32:0) | 7.552 (4.341) | 6.465 (3.971) | -1.17 | Up | 0.2958 (W) |
| TG(18:2_33:0) | 7.591 (3.269) | 6.889 (3.365) | -1.1 | Up | 0.3006 (W) |
| PC aa C40:2 | 0.263 (0.065) | 0.280 (0.080) | 1.07 | Down | 0.3042 |
| PC ae C36:0 | 0.755 (0.172) | 0.803 (0.228) | 1.06 | Down | 0.305 |
| TG(18:0_36:2) | 37.133 (17.628) | 32.724 (14.266) | -1.13 | Up | 0.3104 (W) |
| TG(18:1_32:2) | 70.186 (33.543) | 61.832 (30.674) | -1.14 | Up | 0.3104 (W) |
| ProBetaine | 8.110 (11.546) | 6.422 (9.824) | -1.26 | Up | 0.3129 (W) |
| PC ae C30:1 | 0.200 (0.126) | 0.173 (0.105) | -1.15 | Up | 0.3151 |
| TG(14:0_36:2) | 52.117 (24.882) | 45.683 (22.400) | -1.14 | Up | 0.3154 (W) |
| TG(22:6_34:3) | 2.382 (1.467) | 2.031 (1.224) | -1.17 | Up | 0.3154 (W) |
| DG(18:1_22:6) | 0.379 (0.137) | 0.330 (0.157) | -1.15 | Up | 0.3179 (W) |
| t4-OH-Pro | 10.756 (5.400) | 10.609 (3.487) | -1.01 | Up | 0.3179 (W) |
| C6 (C4:1-DC) | 0.158 (0.068) | 0.124 (0.091) | -1.28 | Up | 0.3196 (W) |
| TG(20:0_34:1) | 2.301 (0.702) | 2.133 (0.755) | -1.08 | Up | 0.3198 |
| Malonate | 4.450 (4.464) | 42.005 (245.382) | 9.44 | Down | 0.3202 (W) |
| TG(18:1_26:0) | 4.479 (4.351) | 4.522 (6.795) | 1.01 | Down | 0.3255 (W) |
| TG(16:0_38:3) | 20.837 (8.543) | 19.278 (9.081) | -1.08 | Up | 0.3281 (W) |
| TG(18:1_36:1) | 87.747 (42.416) | 75.973 (33.525) | -1.15 | Up | 0.3281 (W) |
| TG(18:2_33:1) | 15.758 (7.045) | 14.303 (7.397) | -1.1 | Up | 0.3307 (W) |
| TG(20:2_36:5) | 0.241 (0.100) | 0.219 (0.098) | -1.1 | Up | 0.3315 |
| TG(18:2_36:0) | 7.125 (3.048) | 6.481 (2.885) | -1.1 | Up | 0.3333 (W) |
| C14:1-OH | 0.026 (0.011) | 0.022 (0.015) | -1.2 | Up | 0.3356 (W) |
| Cit | 19.082 (6.643) | 16.989 (5.416) | -1.12 | Up | 0.3438 (W) |
| SM C26:0 | 0.110 (0.032) | 0.118 (0.039) | 1.07 | Down | 0.3438 |
| C8 | 0.300 (0.137) | 0.233 (0.193) | -1.29 | Up | 0.3447 (W) |
| CE(15:1) | 0.625 (0.207) | 0.556 (0.218) | -1.13 | Up | 0.3464 (W) |
| HexCer(d18:2/22:0) | 0.630 (0.272) | 0.645 (0.208) | 1.02 | Down | 0.3464 (W) |
| 3-IAA | 1.565 (1.041) | 1.379 (1.050) | -1.14 | Up | 0.3491 (W) |
| C0 | 16.739 (7.319) | 15.434 (8.082) | -1.08 | Up | 0.3518 (W) |
| PC aa C42:5 | 0.528 (0.153) | 0.496 (0.149) | -1.07 | Up | 0.3541 |
| PC aa C34:1 | 334.056 (75.923) | 317.050 (82.538) | -1.05 | Up | 0.3547 |
| CE(20:1) | 1.601 (1.347) | 1.314 (0.430) | -1.22 | Up | 0.3571 (W) |
| TG(16:0_37:3) | 2.723 (1.121) | 2.536 (1.238) | -1.07 | Up | 0.3572 (W) |
| TG(16:0_36:2) | 708.000 (322.320) | 643.880 (328.583) | -1.1 | Up | 0.3599 (W) |
| TG(18:1_36:2) | 286.081 (144.964) | 247.922 (126.474) | -1.15 | Up | 0.3599 (W) |
| CE(15:0) | 10.284 (3.568) | 9.458 (4.190) | -1.09 | Up | 0.3604 |
| PC aa C36:6 | 1.219 (0.596) | 1.122 (0.630) | -1.09 | Up | 0.3654 (W) |
| TG(18:3_34:3) | 4.701 (2.098) | 4.453 (2.714) | -1.06 | Up | 0.3654 (W) |
| PC aa C42:1 | 0.337 (0.106) | 0.361 (0.125) | 1.07 | Down | 0.3658 |
| DG(16:1_18:0) | 0.292 (0.113) | 0.254 (0.109) | -1.15 | Up | 0.3681 (W) |
| TG(22:0_32:4) | 0.186 (0.058) | 0.173 (0.066) | -1.08 | Up | 0.3689 |
| Lys | 172.161 (51.147) | 162.112 (56.450) | -1.06 | Up | 0.3737 (W) |
| PC aa C36:3 | 220.083 (53.895) | 209.005 (54.918) | -1.05 | Up | 0.3786 |
| TG(18:1_34:4) | 8.178 (3.637) | 7.326 (3.575) | -1.12 | Up | 0.3794 (W) |
| BABA | 0.031 (0.047) | 0.028 (0.028) | -1.11 | Up | 0.3849 (W) |
| TG(22:6_34:1) | 19.196 (11.034) | 17.107 (10.612) | -1.12 | Up | 0.3850 (W) |
| DHEAS | 1.594 (1.555) | 1.373 (1.245) | -1.16 | Up | 0.3879 (W) |
| DG(17:0_17:1) | 0.125 (0.050) | 0.112 (0.056) | -1.12 | Up | 0.3904 (W) |
| DG(14:0_20:0) | 0.063 (0.026) | 0.054 (0.029) | -1.16 | Up | 0.3928 (W) |
| TG(20:1_34:3) | 1.592 (0.685) | 1.445 (0.678) | -1.1 | Up | 0.3936 (W) |
| DG(18:1_20:2) | 0.102 (0.050) | 0.087 (0.062) | -1.17 | Up | 0.3950 (W) |
| TMCA | 0.067 (0.085) | 0.071 (0.062) | 1.06 | Down | 0.4023 (W) |
| lysoPC a C26:0 | 0.182 (0.069) | 0.204 (0.102) | 1.12 | Down | 0.4023 (W) |
| TG(18:3_34:2) | 36.708 (15.180) | 35.070 (18.425) | -1.05 | Up | 0.4082 (W) |
| HexCer(d18:1/20:0) | 0.406 (0.146) | 0.410 (0.127) | 1.01 | Down | 0.4082 (W) |
| TG(18:3_35:2) | 1.023 (0.464) | 0.932 (0.472) | -1.1 | Up | 0.4111 (W) |
| TG(16:0_38:2) | 13.509 (6.050) | 12.442 (5.944) | -1.09 | Up | 0.4171 (W) |
| TG(20:1_34:1) | 12.274 (5.591) | 11.245 (5.733) | -1.09 | Up | 0.4171 (W) |
| TG(18:1_35:2) | 16.162 (7.867) | 14.298 (6.921) | -1.13 | Up | 0.4231 (W) |
| Thr | 194.778 (86.293) | 196.590 (70.841) | 1.01 | Down | 0.4261 (W) |
| DG(18:0_20:4) | 0.058 (0.030) | 0.051 (0.033) | -1.13 | Up | 0.4273 (W) |
| GLCAS | 0.114 (0.084) | 0.110 (0.100) | -1.04 | Up | 0.4291 (W) |
| SM (OH) C22:1 | 14.840 (3.746) | 14.155 (3.796) | -1.05 | Up | 0.4319 |
| SM C22:3 | 0.960 (0.679) | 1.065 (0.622) | 1.11 | Down | 0.4410 (W) |
| HexCer(d18:1/14:0) | 0.031 (0.013) | 0.029 (0.011) | -1.07 | Up | 0.4423 |
| Dimethyl sulfone | 3.839 (3.998) | 8.752 (32.335) | 2.28 | Down | 0.4443 (W) |
| TG(18:2_34:4) | 4.053 (1.819) | 3.734 (1.964) | -1.09 | Up | 0.4445 (W) |
| DG(18:1_18:1) | 7.816 (3.733) | 7.340 (4.587) | -1.06 | Up | 0.4476 (W) |
| PC aa C42:4 | 0.254 (0.077) | 0.259 (0.078) | 1.02 | Down | 0.4507 (W) |
| TG(16:1_36:3) | 68.728 (35.750) | 61.835 (35.577) | -1.11 | Up | 0.4529 (W) |
| TG(16:1_36:5) | 3.082 (1.400) | 2.832 (1.528) | -1.09 | Up | 0.4538 (W) |
| TG(18:3_34:1) | 58.439 (22.707) | 55.575 (26.322) | -1.05 | Up | 0.4538 (W) |
| PC ae C44:3 | 0.148 (0.038) | 0.156 (0.052) | 1.05 | Down | 0.455 |
| DG(16:0_18:2) | 7.318 (2.787) | 6.969 (3.375) | -1.05 | Up | 0.4570 (W) |
| TG(18:1_34:1) | 1104.389 (486.730) | 1005.640 (481.124) | -1.1 | Up | 0.4570 (W) |
| TG(20:2_34:2) | 9.171 (3.655) | 8.875 (4.214) | -1.03 | Up | 0.4696 (W) |
| TG(18:2_38:6) | 4.630 (1.957) | 4.201 (1.823) | -1.1 | Up | 0.4728 (W) |
| C14:2 | 0.060 (0.031) | 0.052 (0.037) | -1.16 | Up | 0.4754 (W) |
| beta-Ala | 3.020 (3.451) | 2.583 (3.060) | -1.17 | Up | 0.4761 (W) |
| PC aa C32:3 | 0.999 (0.260) | 0.966 (0.320) | -1.03 | Up | 0.4792 (W) |
| PC aa C34:3 | 31.419 (10.712) | 29.801 (12.079) | -1.05 | Up | 0.4825 (W) |
| TG(18:1_33:2) | 11.752 (5.362) | 10.871 (5.599) | -1.08 | Up | 0.4825 (W) |
| Cer(d18:0/24:0) | 0.263 (0.079) | 0.277 (0.091) | 1.05 | Down | 0.4852 |
| Cer(d18:1/24:1) | 1.229 (0.343) | 1.224 (0.309) | -1 | Up | 0.4856 (W) |
| SM C24:0 | 20.867 (5.036) | 20.000 (5.823) | -1.04 | Up | 0.4922 |
| PC aa C40:6 | 30.581 (11.370) | 28.765 (11.886) | -1.06 | Up | 0.4996 |
| PC ae C38:1 | 0.045 (0.074) | 0.080 (0.129) | 1.78 | Down | 0.4998 (W) |
| Pro | 155.256 (45.322) | 147.862 (54.630) | -1.05 | Up | 0.5055 (W) |
| Hex2Cer(d18:1/18:0) | 0.180 (0.040) | 0.185 (0.044) | 1.03 | Down | 0.5088 (W) |
| Cer(d18:1/20:0) | 0.343 (0.119) | 0.329 (0.128) | -1.04 | Up | 0.5088 (W) |
| TG(18:2_32:2) | 34.936 (15.547) | 33.406 (18.045) | -1.05 | Up | 0.5155 (W) |
| Hex3Cer(d18:1/24:1) | 0.297 (0.094) | 0.311 (0.098) | 1.05 | Down | 0.518 |
| DG(18:1_20:1) | 0.257 (0.076) | 0.263 (0.090) | 1.03 | Down | 0.5189 (W) |
| C3 | 0.242 (0.063) | 0.235 (0.082) | -1.03 | Up | 0.5189 (W) |
| TG(14:0_36:3) | 51.431 (22.127) | 48.274 (23.612) | -1.07 | Up | 0.5189 (W) |
| Creatinine | 29.236 (12.202) | 30.942 (12.712) | 1.06 | Down | 0.5291 (W) |
| Xanthine | 1.091 (0.944) | 0.901 (0.691) | -1.21 | Up | 0.5319 (W) |
| TG(18:3_36:1) | 5.494 (2.328) | 5.194 (2.464) | -1.06 | Up | 0.5325 (W) |
| Cer(d18:1/23:0) | 1.069 (0.288) | 1.055 (0.297) | -1.01 | Up | 0.5393 (W) |
| PC aa C30:0 | 6.874 (3.197) | 6.269 (2.650) | -1.1 | Up | 0.5394 (W) |
| TG(18:1_34:2) | 788.194 (343.335) | 750.445 (375.806) | -1.05 | Up | 0.5394 (W) |
| lysoPC a C26:1 | 0.115 (0.045) | 0.110 (0.046) | -1.05 | Up | 0.5427 (W) |
| TG(18:0_36:3) | 47.786 (21.794) | 44.721 (20.995) | -1.07 | Up | 0.5428 (W) |
| Asn | 51.511 (18.007) | 47.590 (16.456) | -1.08 | Up | 0.5462 (W) |
| HexCer(d18:2/20:0) | 0.089 (0.033) | 0.085 (0.026) | -1.05 | Up | 0.5522 |
| Cer(d18:2/24:0) | 0.410 (0.150) | 0.387 (0.132) | -1.06 | Up | 0.5532 (W) |
| Gln | 474.306 (123.084) | 441.740 (99.607) | -1.07 | Up | 0.5567 (W) |
| 1-Met-His | 3.471 (1.852) | 3.119 (1.471) | -1.11 | Up | 0.5602 (W) |
| SDMA | 0.557 (0.130) | 0.585 (0.208) | 1.05 | Down | 0.5602 (W) |
| PC ae C42:5 | 2.086 (0.481) | 2.160 (0.607) | 1.04 | Down | 0.5645 |
| TMAO | 3.359 (4.698) | 2.597 (2.114) | -1.29 | Up | 0.5672 (W) |
| PC aa C32:0 | 25.636 (7.744) | 23.990 (6.558) | -1.07 | Up | 0.5707 (W) |
| TG(20:0_32:3) | 1.988 (0.884) | 1.910 (1.011) | -1.04 | Up | 0.5707 (W) |
| DG-O(16:0_20:4) | 0.004 (0.004) | 0.004 (0.005) | 1.13 | Down | 0.5711 (W) |
| CE(22:2) | 0.211 (0.111) | 0.177 (0.079) | -1.19 | Up | 0.5741 (W) |
| CE(22:1) | 0.644 (0.234) | 0.580 (0.284) | -1.11 | Up | 0.5777 (W) |
| TG(16:0_40:8) | 3.566 (1.998) | 3.195 (1.633) | -1.12 | Up | 0.5778 (W) |
| TG(18:1_35:3) | 4.483 (2.103) | 4.061 (1.940) | -1.1 | Up | 0.5814 (W) |
| Cer(d18:1/20:0(OH)) | 1.794 (0.724) | 1.773 (0.454) | -1.01 | Up | 0.5849 (W) |
| Hex3Cer(d18:1/26:1) | 0.274 (0.096) | 0.261 (0.103) | -1.05 | Up | 0.5859 |
| Cer(d18:1/16:0) | 0.571 (0.133) | 0.581 (0.143) | 1.02 | Down | 0.5921 (W) |
| SM C16:1 | 20.831 (5.005) | 20.257 (4.767) | -1.03 | Up | 0.5921 (W) |
| PC aa C42:6 | 0.527 (0.173) | 0.489 (0.149) | -1.08 | Up | 0.5921 (W) |
| PC ae C42:2 | 0.481 (0.125) | 0.499 (0.160) | 1.04 | Down | 0.5928 |
| Sarcosine | 0.622 (0.495) | 0.650 (0.822) | 1.04 | Down | 0.6064 (W) |
| CE(18:2) | 2075.111 (501.775) | 2074.585 (534.010) | -1 | Up | 0.6066 (W) |
| Hex2Cer(d18:1/16:0) | 3.304 (0.972) | 3.418 (0.959) | 1.03 | Down | 0.6091 |
| Hex2Cer(d18:1/26:1) | 0.045 (0.023) | 0.044 (0.021) | -1.02 | Up | 0.6095 (W) |
| TG(18:0_36:5) | 2.631 (1.190) | 2.605 (1.474) | -1.01 | Up | 0.6102 (W) |
| TG(20:1_34:2) | 9.515 (4.199) | 9.153 (4.618) | -1.04 | Up | 0.6138 (W) |
| SM C24:1 | 49.094 (11.097) | 46.988 (11.252) | -1.04 | Up | 0.6138 (W) |
| TG(18:1_34:3) | 107.747 (50.520) | 100.153 (51.772) | -1.08 | Up | 0.6139 (W) |
| lysoPC a C28:0 | 0.161 (0.169) | 0.176 (0.150) | 1.09 | Down | 0.6141 (W) |
| DHA | 3.393 (2.867) | 3.893 (3.993) | 1.15 | Down | 0.6175 (W) |
| PC ae C44:5 | 1.574 (0.434) | 1.632 (0.561) | 1.04 | Down | 0.6185 |
| GLCA | 0.007 (0.008) | 0.006 (0.005) | -1.35 | Up | 0.6201 (W) |
| Hex2Cer(d18:1/24:1) | 0.306 (0.097) | 0.317 (0.102) | 1.04 | Down | 0.6202 |
| PC ae C42:1 | 0.413 (0.094) | 0.401 (0.108) | -1.03 | Up | 0.6242 |
| DG(18:1_18:4) | 0.069 (0.056) | 0.063 (0.053) | -1.1 | Up | 0.6277 (W) |
| PC ae C38:6 | 7.489 (2.512) | 7.621 (2.285) | 1.02 | Down | 0.6285 (W) |
| TG(18:0_36:4) | 20.137 (9.313) | 19.483 (10.077) | -1.03 | Up | 0.6359 (W) |
| Cer(d18:1/18:0) | 0.253 (0.084) | 0.249 (0.065) | -1.02 | Up | 0.6359 (W) |
| DG(18:2_18:3) | 0.857 (0.374) | 0.802 (0.393) | -1.07 | Up | 0.6360 (W) |
| PC aa C40:1 | 0.224 (0.185) | 0.233 (0.201) | 1.04 | Down | 0.6382 (W) |
| CE(22:0) | 0.504 (0.278) | 0.459 (0.298) | -1.1 | Up | 0.6433 (W) |
| TG(20:0_32:4) | 2.058 (0.997) | 2.185 (1.173) | 1.06 | Down | 0.6434 (W) |
| PC ae C38:0 | 2.256 (0.711) | 2.155 (0.761) | -1.05 | Up | 0.6434 (W) |
| TG(18:2_36:1) | 58.994 (27.272) | 55.164 (25.680) | -1.07 | Up | 0.6434 (W) |
| TG(17:2_36:4) | 1.253 (0.496) | 1.195 (0.539) | -1.05 | Up | 0.6509 (W) |
| p-Cresol-SO4 | 20.438 (13.871) | 19.033 (13.090) | -1.07 | Up | 0.6509 |
| HexCer(d18:1/23:0) | 1.889 (0.631) | 1.954 (0.626) | 1.03 | Down | 0.652 |
| TG(17:1_36:4) | 1.769 (0.807) | 1.649 (0.790) | -1.07 | Up | 0.6546 (W) |
| PC aa C36:0 | 1.281 (0.791) | 1.360 (0.695) | 1.06 | Down | 0.6583 (W) |
| TCA | 0.478 (0.989) | 0.575 (1.755) | 1.2 | Down | 0.6584 (W) |
| TG(22:6_34:2) | 15.504 (9.335) | 14.179 (7.953) | -1.09 | Up | 0.6584 (W) |
| Betaine | 8.987 (4.928) | 10.976 (13.749) | 1.22 | Down | 0.6659 (W) |
| PC ae C40:4 | 2.783 (0.631) | 2.853 (0.783) | 1.03 | Down | 0.6673 |
| TG(16:1_36:4) | 23.710 (11.558) | 22.031 (11.721) | -1.08 | Up | 0.6735 (W) |
| TG(18:2_34:1) | 690.889 (300.740) | 668.300 (335.279) | -1.03 | Up | 0.6735 (W) |
| TG(14:0_36:4) | 18.442 (8.134) | 18.189 (10.238) | -1.01 | Up | 0.6773 (W) |
| Cer(d16:1/24:0) | 0.188 (0.088) | 0.177 (0.081) | -1.06 | Up | 0.6773 (W) |
| TG(16:0_36:5) | 30.594 (12.619) | 30.062 (15.799) | -1.02 | Up | 0.6773 (W) |
| Choline | 3.944 (1.968) | 4.143 (2.228) | 1.05 | Down | 0.6848 (W) |
| Cer(d16:1/23:0) | 0.131 (0.061) | 0.131 (0.051) | -1 | Up | 0.6849 (W) |
| TG(18:1_36:4) | 99.108 (47.512) | 93.543 (45.969) | -1.06 | Up | 0.6849 (W) |
| C4 | 0.207 (0.151) | 0.176 (0.067) | -1.17 | Up | 0.6887 (W) |
| TG(18:1_36:3) | 260.564 (128.497) | 241.987 (121.620) | -1.08 | Up | 0.6888 (W) |
| TG(22:2_32:4) | 0.177 (0.082) | 0.170 (0.082) | -1.04 | Up | 0.6887 (W) |
| GCDCA | 0.778 (0.653) | 0.686 (0.485) | -1.13 | Up | 0.6888 (W) |
| HexCer(d18:2/24:0) | 0.804 (0.333) | 0.796 (0.262) | -1.01 | Up | 0.6888 (W) |
| SM (OH) C24:1 | 1.043 (0.289) | 1.016 (0.295) | -1.03 | Up | 0.6904 |
| PC aa C28:1 | 4.315 (1.401) | 4.197 (1.169) | -1.03 | Up | 0.691 |
| Hex2Cer(d18:1/20:0) | 0.080 (0.022) | 0.077 (0.019) | -1.03 | Up | 0.6925 (W) |
| PC ae C38:4 | 16.305 (4.652) | 15.910 (4.153) | -1.02 | Up | 0.6968 |
| Cer(d18:1/25:0) | 0.391 (0.129) | 0.402 (0.112) | 1.03 | Down | 0.7006 |
| PC ae C34:0 | 1.885 (0.589) | 1.835 (0.546) | -1.03 | Up | 0.7025 |
| TG(18:3_36:2) | 18.976 (8.478) | 18.064 (8.543) | -1.05 | Up | 0.7041 (W) |
| Lac | 3486.194 (1140.909) | 3523.715 (1675.457) | 1.01 | Down | 0.7058 (W) |
| PC ae C32:1 | 3.746 (1.028) | 3.865 (1.000) | 1.03 | Down | 0.7080 (W) |
| Succinate | 14.186 (8.111) | 13.002 (5.655) | -1.09 | Up | 0.7119 (W) |
| PC ae C36:1 | 9.277 (2.470) | 9.071 (2.401) | -1.02 | Up | 0.7135 |
| Hex2Cer(d18:1/22:0) | 0.188 (0.052) | 0.190 (0.056) | 1.01 | Down | 0.7196 (W) |
| Ind-SO4 | 3.220 (2.255) | 3.359 (2.590) | 1.04 | Down | 0.7197 (W) |
| PC aa C32:2 | 9.037 (3.905) | 8.722 (3.738) | -1.04 | Up | 0.7206 |
| TG(20:1_32:3) | 0.469 (0.190) | 0.463 (0.226) | -1.01 | Up | 0.7235 (W) |
| SM C18:0 | 31.794 (7.795) | 31.566 (7.144) | -1.01 | Up | 0.7274 (W) |
| CE(22:6) | 68.978 (23.459) | 66.915 (27.747) | -1.03 | Up | 0.7288 |
| HexCer(d18:1/26:1) | 0.105 (0.026) | 0.102 (0.033) | -1.02 | Up | 0.7297 |
| C9 | 0.038 (0.015) | 0.036 (0.016) | -1.06 | Up | 0.7350 (W) |
| SM (OH) C14:1 | 7.943 (2.307) | 7.771 (2.149) | -1.02 | Up | 0.7363 |
| DG(18:1_18:2) | 19.961 (9.248) | 19.631 (11.087) | -1.02 | Up | 0.7392 (W) |
| Cer(d18:0/26:1(OH)) | 0.207 (0.069) | 0.194 (0.086) | -1.07 | Up | 0.7431 (W) |
| Tyr | 49.550 (17.466) | 47.839 (19.533) | -1.04 | Up | 0.7431 (W) |
| TG(16:0_36:3) | 765.333 (331.801) | 745.490 (374.174) | -1.03 | Up | 0.7431 (W) |
| Hex2Cer(d18:1/14:0) | 0.307 (0.098) | 0.315 (0.116) | 1.03 | Down | 0.7449 |
| Hex3Cer(d18:1_20:0) | 0.086 (0.030) | 0.088 (0.025) | 1.02 | Down | 0.7483 |
| 3-IPA | 0.436 (0.439) | 0.451 (0.534) | 1.03 | Down | 0.7505 (W) |
| SM (OH) C16:1 | 4.175 (1.104) | 4.145 (1.009) | -1.01 | Up | 0.7550 (W) |
| TG(18:2_36:4) | 52.522 (28.667) | 51.874 (31.768) | -1.01 | Up | 0.7550 (W) |
| Cer(d18:0/26:1) | 0.012 (0.010) | 0.012 (0.011) | 1.04 | Down | 0.7568 (W) |
| HexCer(d18:1/26:0) | 0.152 (0.044) | 0.155 (0.048) | 1.02 | Down | 0.7584 |
| TG(18:2_33:2) | 6.419 (3.011) | 6.221 (3.324) | -1.03 | Up | 0.7589 (W) |
| C16:1 | 0.021 (0.019) | 0.020 (0.019) | -1.06 | Up | 0.7590 (W) |
| TG(18:3_36:3) | 15.813 (7.781) | 15.628 (8.811) | -1.01 | Up | 0.7668 (W) |
| TG(20:2_34:3) | 1.610 (0.682) | 1.506 (0.664) | -1.07 | Up | 0.7668 (W) |
| Trigonelline | 0.537 (1.131) | 0.501 (0.890) | -1.07 | Up | 0.7669 (W) |
| D-Glucose | 2978.611 (1311.496) | 2919.765 (1665.056) | -1.02 | Up | 0.7682 (W) |
| TG(18:2_36:2) | 152.208 (73.956) | 144.654 (72.270) | -1.05 | Up | 0.7708 (W) |
| HexCer(d16:1/22:0) | 0.240 (0.104) | 0.227 (0.086) | -1.06 | Up | 0.7708 (W) |
| TG(18:2_36:3) | 128.064 (65.741) | 124.975 (67.266) | -1.02 | Up | 0.7708 (W) |
| 1-Methylhistidine | 97.525 (37.747) | 97.062 (47.796) | -1 | Up | 0.7708 (W) |
| Cer(d18:1/18:0(OH)) | 0.077 (0.056) | 0.083 (0.059) | 1.08 | Down | 0.7759 (W) |
| HexCer(d18:1/16:0) | 1.565 (0.439) | 1.594 (0.472) | 1.02 | Down | 0.7826 |
| 3-Met-His | 5.385 (6.596) | 5.998 (6.338) | 1.11 | Down | 0.7840 (W) |
| TG(18:2_35:1) | 10.842 (5.217) | 10.206 (4.802) | -1.06 | Up | 0.7868 (W) |
| Cer(d18:2/20:0) | 0.076 (0.022) | 0.076 (0.024) | 1 | Down | 0.7906 (W) |
| Met | 23.708 (7.611) | 23.034 (8.117) | -1.03 | Up | 0.7908 (W) |
| PC aa C38:6 | 124.031 (43.212) | 121.302 (46.169) | -1.02 | Up | 0.7916 |
| PC ae C40:6 | 4.973 (1.471) | 5.061 (1.472) | 1.02 | Down | 0.7942 |
| Cer(d18:1/24:0) | 2.513 (0.734) | 2.510 (0.870) | -1 | Up | 0.7948 (W) |
| Leu | 85.897 (25.368) | 85.806 (36.042) | -1 | Up | 0.7948 (W) |
| TG(17:2_36:3) | 0.650 (0.247) | 0.628 (0.275) | -1.03 | Up | 0.7948 (W) |
| TG(18:2_34:3) | 52.064 (25.390) | 51.023 (27.598) | -1.02 | Up | 0.7988 (W) |
| PC ae C44:6 | 1.487 (0.462) | 1.514 (0.476) | 1.02 | Down | 0.8041 |
| Cer(d18:2/24:1) | 0.196 (0.069) | 0.186 (0.051) | -1.05 | Up | 0.8068 (W) |
| HexCer(d16:1/24:0) | 0.113 (0.050) | 0.111 (0.039) | -1.02 | Up | 0.8084 |
| C5 | 0.114 (0.055) | 0.111 (0.038) | -1.03 | Up | 0.8109 (W) |
| His | 92.681 (27.911) | 94.544 (36.358) | 1.02 | Down | 0.8109 (W) |
| Serotonin | 0.443 (0.324) | 0.458 (0.333) | 1.03 | Down | 0.8149 (W) |
| AconAcid | 2.305 (1.699) | 2.362 (2.177) | 1.02 | Down | 0.8149 (W) |
| Urea | 113.583 (130.120) | 148.268 (219.892) | 1.31 | Down | 0.8149 (W) |
| Taurine | 52.842 (30.703) | 49.367 (24.929) | -1.07 | Up | 0.8149 (W) |
| Cer(d18:1/14:0) | 0.079 (0.021) | 0.081 (0.031) | 1.02 | Down | 0.8187 |
| PC ae C36:5 | 12.625 (4.692) | 12.118 (3.749) | -1.04 | Up | 0.8189 (W) |
| Creatinine.1 | 53.189 (10.813) | 52.623 (15.499) | -1.01 | Up | 0.8392 (W) |
| HexCer(d18:1/24:0) | 1.853 (0.657) | 1.881 (0.600) | 1.02 | Down | 0.8468 |
| Cer(d18:2/23:0) | 0.125 (0.051) | 0.122 (0.036) | -1.02 | Up | 0.8474 (W) |
| TCDCA | 0.379 (0.517) | 0.402 (0.620) | 1.06 | Down | 0.8515 (W) |
| Hex2Cer(d18:1/24:0) | 0.134 (0.039) | 0.136 (0.041) | 1.01 | Down | 0.8539 |
| TG(17:1_36:3) | 4.093 (2.038) | 3.844 (1.865) | -1.06 | Up | 0.8555 (W) |
| HexCer(d18:2/18:0) | 0.044 (0.016) | 0.041 (0.014) | -1.08 | Up | 0.8595 (W) |
| SM C18:1 | 14.315 (3.995) | 13.754 (3.234) | -1.04 | Up | 0.8596 (W) |
| SM C20:2 | 0.792 (0.341) | 0.780 (0.266) | -1.02 | Up | 0.8627 |
| Asp | 16.042 (6.712) | 15.793 (6.393) | -1.02 | Up | 0.8637 (W) |
| SM C26:1 | 0.259 (0.079) | 0.245 (0.068) | -1.06 | Up | 0.8637 (W) |
| TG(18:1_36:5) | 16.301 (7.676) | 15.955 (8.480) | -1.02 | Up | 0.8637 (W) |
| DG(18:2_18:2) | 11.916 (6.023) | 12.089 (6.554) | 1.01 | Down | 0.8678 (W) |
| Cer(d18:2/16:0) | 0.118 (0.037) | 0.115 (0.031) | -1.02 | Up | 0.8719 (W) |
| PC ae C36:4 | 20.407 (5.699) | 20.165 (5.056) | -1.01 | Up | 0.8842 (W) |
| DG(16:0_20:4) | 0.089 (0.028) | 0.081 (0.037) | -1.1 | Up | 0.8882 (W) |
| GUDCA | 0.047 (0.051) | 0.041 (0.040) | -1.13 | Up | 0.8924 (W) |
| DG(18:1_18:3) | 1.254 (0.486) | 1.239 (0.496) | -1.01 | Up | 0.8955 |
| PC ae C38:5 | 18.652 (5.133) | 18.309 (4.442) | -1.02 | Up | 0.8965 (W) |
| TG(18:2_35:3) | 2.460 (1.241) | 2.431 (1.194) | -1.01 | Up | 0.8965 (W) |
| TG(18:3_36:4) | 6.624 (3.797) | 6.819 (4.866) | 1.03 | Down | 0.8965 (W) |
| C14:1 | 0.052 (0.020) | 0.051 (0.021) | -1.01 | Up | 0.9006 (W) |
| AABA | 11.197 (7.368) | 11.390 (7.375) | 1.02 | Down | 0.9048 (W) |
| C12 | 0.125 (0.067) | 0.121 (0.071) | -1.03 | Up | 0.9076 (W) |
| HexCer(d18:2/23:0) | 0.307 (0.132) | 0.298 (0.112) | -1.03 | Up | 0.9089 (W) |
| SM C16:0 | 145.939 (29.926) | 145.296 (34.048) | -1 | Up | 0.9171 (W) |
| C10 | 0.462 (0.153) | 0.459 (0.158) | -1.01 | Up | 0.9211 |
| GCA | 0.534 (0.691) | 0.494 (0.479) | -1.08 | Up | 0.9213 (W) |
| Ile | 51.425 (15.724) | 52.600 (23.077) | 1.02 | Down | 0.9254 (W) |
| AA | 3.963 (3.706) | 3.795 (2.386) | -1.04 | Up | 0.9288 (W) |
| PC ae C30:0 | 0.423 (0.158) | 0.419 (0.158) | -1.01 | Up | 0.9295 (W) |
| PC aa C30:2 | 0.151 (0.093) | 0.151 (0.098) | 1 | Down | 0.9337 (W) |
| Cer(d18:1/26:0) | 0.053 (0.014) | 0.052 (0.017) | -1.01 | Up | 0.9368 |
| alpha-AAA | 0.967 (0.619) | 0.880 (0.417) | -1.1 | Up | 0.9378 (W) |
| TG(18:2_35:2) | 8.525 (4.203) | 8.174 (3.942) | -1.04 | Up | 0.9378 (W) |
| Cer(d16:1/18:0) | 0.096 (0.024) | 0.096 (0.034) | -1.01 | Up | 0.9391 |
| PC ae C32:2 | 0.889 (0.237) | 0.896 (0.202) | 1.01 | Down | 0.9419 (W) |
| PC ae C40:5 | 3.663 (0.895) | 3.630 (0.940) | -1.01 | Up | 0.9419 (W) |
| PC ae C34:1 | 14.285 (3.444) | 14.343 (3.866) | 1 | Down | 0.9461 (W) |
| Hex3Cer(d18:1/16:0) | 1.345 (0.324) | 1.336 (0.366) | -1.01 | Up | 0.9502 (W) |
| PC aa C36:1 | 57.989 (17.772) | 56.422 (17.772) | -1.03 | Up | 0.9502 (W) |
| TG(17:1_38:6) | 0.180 (0.064) | 0.175 (0.068) | -1.03 | Up | 0.9502 (W) |
| PC ae C38:3 | 5.040 (1.198) | 4.951 (1.319) | -1.02 | Up | 0.9585 (W) |
| PC ae C40:1 | 1.157 (0.385) | 1.119 (0.359) | -1.03 | Up | 0.9668 (W) |
| TG(22:3_30:2) | 0.057 (0.036) | 0.053 (0.036) | -1.07 | Up | 0.9703 (W) |
| Betaine.1 | 15.697 (3.584) | 15.616 (4.044) | -1.01 | Up | 0.9751 (W) |
| TG(17:0_36:4) | 3.569 (1.819) | 3.415 (1.579) | -1.05 | Up | 0.9792 (W) |
| PC aa C42:2 | 0.204 (0.048) | 0.204 (0.056) | 1 | Down | 0.9809 |
| TG(18:2_34:2) | 440.417 (211.494) | 444.770 (238.848) | 1.01 | Down | 0.9875 (W) |
| PC aa C40:3 | 0.449 (0.102) | 0.449 (0.114) | 1 | Down | 0.9895 |
| Cer(d18:1/22:0) | 1.033 (0.271) | 1.039 (0.305) | 1.01 | Down | 0.9917 (W) |
| TG(16:0_36:4) | 287.033 (135.815) | 289.091 (151.426) | 1.01 | Down | 0.9958 (W) |
| TG(17:0_36:3) | 9.446 (4.743) | 8.973 (4.260) | -1.05 | Up | 1.0000 (W) |
| TG(18:2_36:5) | 9.018 (5.185) | 9.483 (6.795) | 1.05 |  | 1.0000 (W) |
