# Supplementary material for: Metabolomic prediction of severe maternal and newborn complications in preeclampsia
Source: Metabolomics. 2024 May 18;20(3):56. doi: 10.1007/s11306-024-02123-0 (PMC11102370; doi:10.1007/s11306-024-02123-0)
Supplement: Supplementary file 3 — Supplementary file3 (DOCX 27 KB) [file 11306_2024_2123_MOESM3_ESM.docx]

**Supplemental Methods Section**

***^1^H NMR based metabolomic analysis***

For NMR data collection samples were prepared using a modified version of the method as described by Mercier et al [1]. Specimens were thawed for metabolomic analysis as described by Graham et al [2, 3]. A total of 300 µl of serum were filtered through pre-washed (x7) 3.5 KDa filters (Amicon Micron YM-3; Sigma-Aldrich, St. Louis, MO) via centrifugation at 13,000 *g,* at 4°C for 30 min. A total of 35 µl of D_2_O and 30 µl of 11.77 mM sodium 2,2-dimethyl-2-2silapentane-5-sulfonate (DSS (D6) in 50-mmol NaH_2_PO_4_ buffer (pH7) were added to 285 µl of the filtrate. Using a liquid handler system (Bruker Biospin, USA) 200 µl of the mixture was transferred to a 3 mm NMR tube for analysis. All ^1^H NMR spectra were acquired as previously described by Ravanbakhsh et al and Graham et al., (2016) [3, 4]. Data collection took place on a Bruker Avance III HD 600 MHz spectrometer (Bruker-Biospin, MA, USA) equipped with a 5 mm TCI cryo-probe at 300 K. Two hundred and fifty-six transients were acquired for each sample and chemical shifts (δ) are reported in parts per million (ppm). The singlet at 0.00 ppm produced by the methyl groups of the internal standard 4,4-dimethyl-4-silapentane-1-sulfonic acid (DSS-d6) was used for spectral referencing and quantification. All collected spectra were analyzed using a custom library of 64 metabolites using Bayesil.

***Chemicals and Reagents ( what were these used for the NMR or MSMS analyses)***

LC-MS grade Acetonitrile, Methanol, Isopropyl alcohol, Formic acid (≥99.0 % purity) were obtained from Fisher Scientific (Hanover Park, IL, USA). LC-MS grade Ethanol, Pyridine and Phenylisothiocyanate were purchased from Sigma Aldrich (St Louis, MO, USA). Milli-Q Water was used for the aqua mobile phase (EMD Millipore, Billerica, MA, USA).

***DI-UPLC-MS/MS Analysis***

Serum samples were prepared as per the manufacturer’s instructions (Biocrates Life Sciences, AG, Innsbruck, Austria). Serum samples and calibration standards were thawed on ice. Serum samples were subsequently mixed for 10s and centrifuged at 10,000 *g* at 4^°^C for 10 minutes. Calibration standards and quality controls (QC) were dissolved in 100 uL of H_2_0 and mixed at 1200 rpm for 15 minutes. 10 uL of serum, calibration standards, QCs, and phosphate buffer solution were added to the 96 well plate. The plate was dried under nitrogen for 30 minutes. All samples and standards were included in a premix of phenylisothiocyanate (PITC) at room temperature for 60 minutes for derivatization purposes and subsequently dried under nitrogen for 60 minutes. Samples were extracted in 5 mM ammonium acetate in methanol for 30 minutes using an orbital shaker and the extracts collected by centrifuging the preparation plate at 500 *g* for 2 minutes. Sample extracts were diluted with H_2_O (1:1) for the liquid chromatography (LC) phase of the analysis. For the flow injection analysis (FIA), 10 uL of sample extract was mixed with 490 uL of the kit solvent in a separate plate, LC and FIA plates were sealed, mixed for 10 minutes at 600 rpm at room temperature and placed into the thermostatically controlled autosampler for analysis.

***DI-UPLC-MS/MS***

Serum extracts were analyzed using an Exion liquid chromatography unit coupled with a QTRAP 6500+ mass spectrometer (AB SCIEX LLC; Redwood city, CA, USA). For UPLC ( ? what does the abbreviation stand for) analysis, serum sample extracts were separated using the MxP Quant 500 C18 column with attached guard and precolumn mixer (Biocrates Life Sciences, AG, Innsbruck, Austria). The mobile phase consisted of A: H_2_0 and formic acid (0.2%); B: MeCN and 4 formic acid (0.2%) delivered at a flow rate of 0.8mL/min with a gradient of B: 0-100% over 4.50 minutes. Eluent %B was increased to 1.00mL/min flow rate and maintained at 100% for 30 seconds followed by a rapid return to the initial conditions for 70 seconds to equilibrate the column. Both positive and negative mode gradient was 5.80 minutes long. The negative mode acquisition gradient differed from positive mode with a difference in %B composition between 2.00-4.50 minutes. The injection volume was 5 uL for positive data acquisition and 15 uL for the negative run. Wash solvent composition consisted of H_2_O: MeOH: MeCN: IPA (v/v).

***FIA-MS/MS Analysis***

Q500 Kit offers direct flow injections (FIA) for lipid analysis. An isocratic method was performed using the kit provided solvent (290 mL MeOH: 1 ampule of FIA additives). The isocratic mobile phase (B: 100% MeOH) was delivered at low flowrate of 0.03mL/min. The injection volume was 20 uL for both positive and negative mode acquisitions. All data were extracted using the MetIDQ software following Biocrates instructions (Biocrates, Innsbruck, Austria).

**Quality Control**

Three different concentration (low, mid, and high) ranges of QC samples were provided by the manufacturer (Biocrates, Innsbruck, Austria).

***Metabolite set enrichment and network analysis***

Metabolite set enrichment analysis (MSEA), which determines if a group of functionally related metabolites in different biochemical pathways are altered, was used to identify biologically meaningful patterns in metabolite concentrations. These patterns were directly related to the pathogenesis of PreE based assessment by MetaboAnalyst (v 5.0) [5]. Homo sapiens (human) pathway library was chosen and all the compounds in the selected pathways were used when referencing the specific metabolome. MSEA eliminates the preselection of compounds in which significance is based on arbitrary cutoff thresholds. This allows us to identify smaller, but persistent biochemical changes that would escape recognition with the use of more standard approaches. The fold enrichment and p-values were used to generate a bar graph to visually represent the results of the MSEA in a visual form. Metabolite network pathways based on metabolites with a raw p-value <.05 and their corresponding KEGG (http://www.genome.jp/kegg/) identification numbers were evaluated using Metscape, a cytoscape plug in [6].

References:

1. Mercier, P., et al., *Towards automatic metabolomic profiling of high-resolution one-dimensional proton NMR spectra.* J Biomol NMR, 2011. **49**(3-4): p. 307-23.

2. Graham, S.F., et al., *Metabolomic profiling of brain from infants who died from Sudden Infant Death Syndrome reveals novel predictive biomarkers.* J Perinatol, 2017. **37**(1): p. 91-97.

3. Graham, S.F., et al., *Metabolic signatures of Huntington's disease (HD): (1)H NMR analysis of the polar metabolome in post-mortem human brain.* Biochim Biophys Acta, 2016. **1862**(9): p. 1675-84.

4. Ravanbakhsh, S., et al., *Accurate, fully-automated NMR spectral profiling for metabolomics.* PLoS One, 2015. **10**(5): p. e0124219.

5. Pang, Z., et al., *MetaboAnalyst 5.0: narrowing the gap between raw spectra and functional insights.* Nucleic Acids Res, 2021. **49**(W1): p. W388-W396.

6. Karnovsky, A., et al., *Metscape 2 bioinformatics tool for the analysis and visualization of metabolomics and gene expression data.* Bioinformatics, 2012. **28**(3): p. 373-80.
